# Discovery of a Small Non-AUG-Initiated ORF in Poleroviruses and Luteoviruses That Is Required for Long-Distance Movement

**DOI:** 10.1371/journal.ppat.1004868

**Published:** 2015-05-06

**Authors:** Ekaterina Smirnova, Andrew E. Firth, W. Allen Miller, Danièle Scheidecker, Véronique Brault, Catherine Reinbold, Aurélie M. Rakotondrafara, Betty Y.-W. Chung, Véronique Ziegler-Graff

**Affiliations:** 1 Institut de Biologie Moléculaire des Plantes CNRS-UPR 2357, Université de Strasbourg, Strasbourg, France; 2 Department of Pathology, University of Cambridge, Cambridge, United Kingdom; 3 Department of Plant Pathology and Microbiology, Iowa State University, Ames, Iowa, United States of America; 4 UMR 1131 SVQV INRA-UDS, Colmar, France; 5 Department of Plant Pathology, University of Wisconsin, Madison, Wisconsin, United States of America; 6 Department of Plant Sciences, University of Cambridge, Cambridge, United Kingdom; University of California Riverside, UNITED STATES

## Abstract

Viruses in the family *Luteoviridae* have positive-sense RNA genomes of around 5.2 to 6.3 kb, and they are limited to the phloem in infected plants. The *Luteovirus* and *Polerovirus* genera include all but one virus in the *Luteoviridae*. They share a common gene block, which encodes the coat protein (ORF3), a movement protein (ORF4), and a carboxy-terminal extension to the coat protein (ORF5). These three proteins all have been reported to participate in the phloem-specific movement of the virus in plants. All three are translated from one subgenomic RNA, sgRNA1. Here, we report the discovery of a novel short ORF, termed ORF3a, encoded near the 5’ end of sgRNA1. Initially, this ORF was predicted by statistical analysis of sequence variation in large sets of aligned viral sequences. ORF3a is positioned upstream of ORF3 and its translation initiates at a non-AUG codon. Functional analysis of the ORF3a protein, P3a, was conducted with *Turnip yellows virus* (TuYV), a polerovirus, for which translation of ORF3a begins at an ACG codon. ORF3a was translated from a transcript corresponding to sgRNA1 in vitro, and immunodetection assays confirmed expression of P3a in infected protoplasts and in agroinoculated plants. Mutations that prevent expression of P3a, or which overexpress P3a, did not affect TuYV replication in protoplasts or inoculated *Arabidopsis thaliana* leaves, but prevented virus systemic infection (long-distance movement) in plants. Expression of P3a from a separate viral or plasmid vector complemented movement of a TuYV mutant lacking ORF3a. Subcellular localization studies with fluorescent protein fusions revealed that P3a is targeted to the Golgi apparatus and plasmodesmata, supporting an essential role for P3a in viral movement.

## Author Summary

In order to maximize coding capacity, RNA viruses often encode overlapping genes and use unusual translational control mechanisms. Plant viruses express proteins required for movement of the virus through the plant, often from non-canonically translated open reading frames (ORFs). Viruses in the economically important Luteoviridae family are confined to the phloem (vascular) tissue, probably due to their specialized phloem-specific movement proteins. These proteins are translated from one viral mRNA, sgRNA1, via initiation at more than one AUG codon to express overlapping genes, and by ribosomal read-through of a stop codon. Here, we describe yet another gene translated from sgRNA1, ORF3a. Translation of ORF3a initiates at a non-standard (not AUG) start codon. We found that ORF3a is not required for viral genome replication, but is required for long-distance movement of the virus in the plant. The movement function could be restored *in trans* by providing the ORF3a product, P3a, from another viral or plasmid vector. P3a localizes in the Golgi apparatus and adjacent to the plasmodesmata, supporting a role in intercellular movement. In summary, we used a powerful bioinformatic tool to discover a cryptic gene whose product is required for movement of a phloem-specific plant virus, revealing multiple levels of translational control that regulate expression of four proteins from a single mRNA.

## Introduction

RNA viruses are models of efficiency in compressing maximum information, such as coding and regulatory signals, into minimum sequence space. To do this, RNA viruses often employ noncanonical translation mechanisms [1, 2]. For example, many viruses encode genes in overlapping open reading frames (ORFs), some of which can be very short. Also, the arrangement of the ORFs themselves can regulate their expression. To decipher a virus life cycle, it is imperative to identify all the coding regions and to understand their function and how they are regulated. However, small functional ORFs, often lacking conventional initiation sites, can be very difficult to detect. Thus, specialized bioinformatic tools are often required to detect key viral genes. Here we use such tools to identify an essential ORF conserved in the two main genera in the *Luteoviridae* family, and provide evidence of its role in infection.

**Fig 1:**
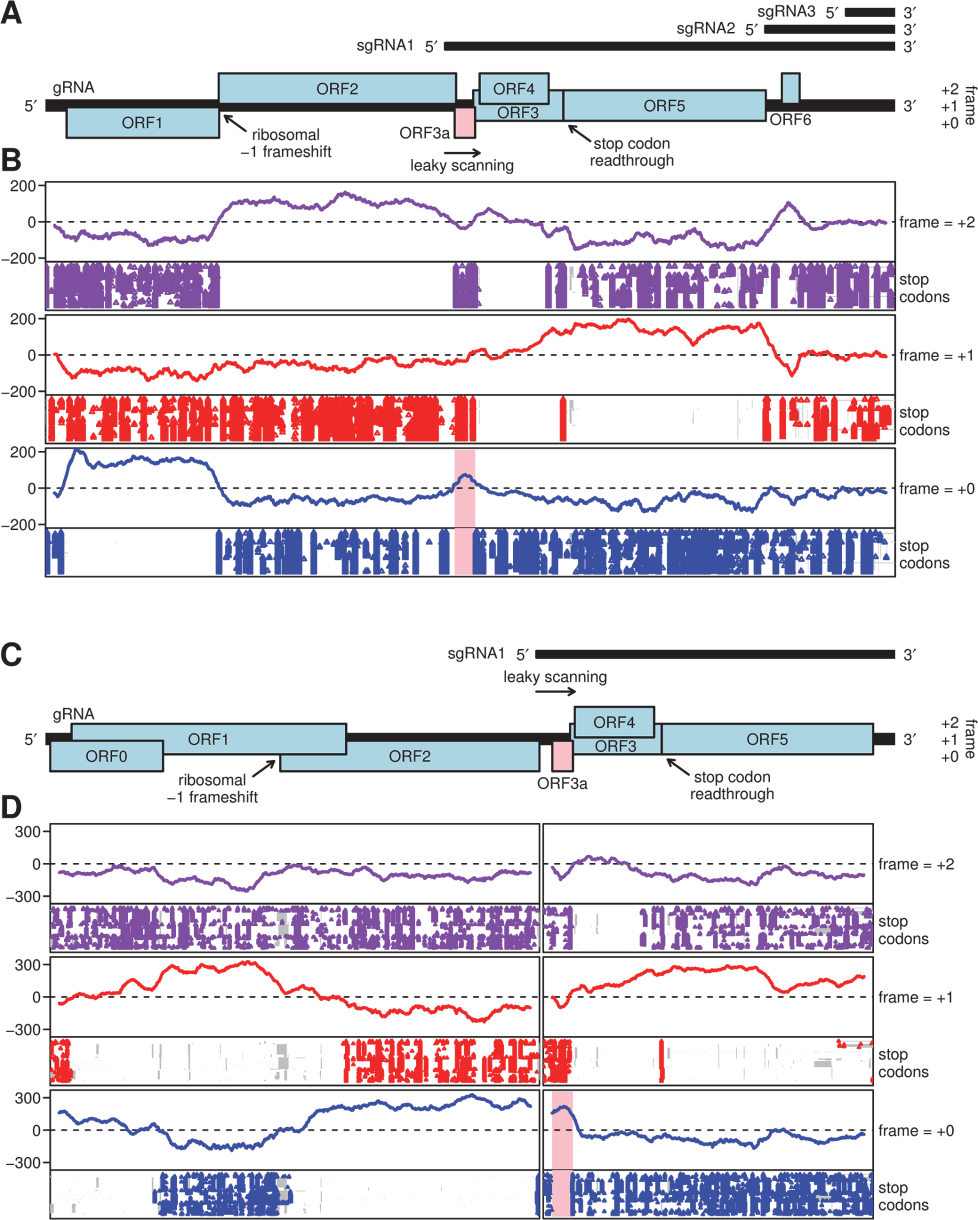
Coding potential analyses of barley yellow dwarf luteoviruses and poleroviruses. **A.** Map of the ~5.7 kb *Barley yellow dwarf virus* (BYDV) genome, including the newly identified ORF3a (pink). Subgenomic RNA start sites are from Kelly et al. [30]. ORF1 and (via ribosomal frameshifting) ORF2 are translated from the genomic RNA. ORFs 3a, 3, 4 and 5 are expressed from sgRNA1, with translation of ORF3a predicted to be dependent on non-AUG initiation, translation of ORFs 3 and 4 being dependent on leaky scanning, and ORF5 translated by readthrough of the ORF3 stop codon. ORF6 may be translated from sgRNA2. **B.** MLOGD analysis, using a 40-codon sliding window, of the coding potential (lines) and positions of stop codons in each aligned sequence (points) in each of the three forward reading frames. The analysis is based on 76 aligned BYDV sequences, including serotypes PAV, PAS, MAV, GAV and Ker-II. Positive MLOGD scores indicate that the sequence is likely to be coding in that reading frame [37]. A conserved absence of stop codons provides independent support for a coding assignment. The pale pink rectangle in the panel corresponding to the +0 frame (blue) indicates the newly discovered ORF3a. To map the analysis onto the coordinates (and reading frames) of a specific sequence, all alignment columns with gaps in a chosen reference sequence, NC_004750.1 (BYDV-PAV), were removed. Remaining alignment gaps in non-reference sequences are indicated with grey rectangles in the stop codon plots. **C.** Map of the ~5.6 kb *Turnip yellows virus* (TuYV) genome, including the newly identified ORF3a (pink). ORFs 0, 1 and 2 are translated from the genomic RNA with expression of ORF1 being dependent on leaky scanning and translation of ORF2 via a -1 ribosomal frameshift. ORFs 3a, 3, 4 and 5 are translated from sgRNA1, with expression of ORF3a being dependent on non-AUG initiation, translation of ORFs 3 and 4 via leaky scanning, and of ORF5 via stop codon readthrough. **D.** MLOGD analysis was performed and displayed as in panel B. The analysis is based on 97 polerovirus sequences aligned separately within the 5' and 3' gene blocks. NC_003743.1 (TuYV) was used as the reference sequence.

Viruses in the economically important *Luteoviridae* family are paragons of translational control. They employ leaky scanning to initiate translation at separate start codons, ribosomal frameshifting, and stop codon readthrough to express various genes, some of which overlap (Fig 1). The *Luteoviridae* family comprises over 33 viruses distributed among three genera, including the wide-spread *Barley yellow dwarf virus* (BYDV) in genus *Luteovirus*, and *Potato leafroll virus* (PLRV), *Turnip yellows virus* (TuYV) and *Cereal yellow dwarf virus* (CYDV) in genus *Polerovirus*, and *Pea enation mosaic virus 1* (PEMV1), the sole member of genus *Enamovirus* [3]. All *Luteoviridae* species are transmitted in a persistent and circulative manner by aphids, but they do not replicate in the aphid, and all but PEMV are confined to the phloem in the plant [4]. Genus *Luteovirus* differs from the others in that the RNA-dependent RNA polymerase (RdRp) and the translational and replication control signals throughout the genome resemble those of the *Tombusviridae* family [5]. In addition, like the *Tombusviridae*, the genome of the *Luteovirus* genus members has no 5’ modification [6], whereas the genomes of poleroviruses and the enamovirus have a genome-linked protein (VPg) covalently attached to the 5’ end [7, 8].

Viruses in all three genera have a 5.2 to 6.3 kb positive-sense RNA genome from which a subgenomic mRNA (sgRNA1) is generated in infected cells [9]. sgRNA1 consists of the 3’-terminal half of the genome in genus *Luteovirus*, or the 3’-terminal third of the genome in the *Polerovirus* and *Enamovirus* genera (Fig 1A and 1C). sgRNA1 serves as the mRNA for translation of ORFs 3, 4, and 5. ORF3 encodes the coat protein (CP), ORF4 codes for P4 and is embedded within the CP ORF but in a different reading frame. ORF5 is translated by in-frame readthrough of the CP ORF stop codon, a process stimulated by sequences adjacent to, and far downstream of, the leaky stop codon [10, 11]. Thus, the translation product of ORF5 (RTD; readthrough domain) does not exist on its own, but is present only as a C-terminal extension of the CP in the CP-RTD protein. The icosahedral (T = 3) virion contains 180 copies of the CP, of which, in the case of BYDV, an estimated 10 to 25% are present in the form of CP-RTD [12]. The RTD is required for aphid transmission [13, 14] and in some viruses also for virus movement in certain plant hosts [13–15]. P4 is likely a cell-to-cell movement protein [16–18]. The sole official enamovirus, PEMV1, lacks ORF4, has a truncated RTD and depends on a helper virus, PEMV2 (genus *Umbravirus*), for efficient movement in the plant [19].

Translation of ORF4 depends on a leaky scanning mechanism whereby some scanning 40S ribosomal subunits fail to initiate at the ORF3 AUG initiation codon and instead continue scanning to the downstream ORF4 AUG initiation codon [20]. This mechanism is facilitated by the generally poor context of the ORF3 initiation codon. In mammals, potential initiation codons with an A at -3, or a G at -3 and a G at +4, (where the A of the AUG is nucleotide +1) may be regarded as being in a ‘strong’ context for initiation [21], while other contexts facilitate leaky scanning. Similar context rules appear to apply in plants [22–24]. Leaky scanning may also be facilitated by the use of non-AUG initiation codons. The near-cognate codons CUG, GUG, ACG, AUU, AUA, UUG and AUC are, under certain circumstances, able to support a significant level of initiation (typically 2–15% of the level of initiation at an AUG codon in a similar context), with CUG being the most efficient non-AUG initiation codon in many systems [1, 25]. Initiation at non-AUG codons normally requires a strong initiation context, but may also be enhanced in less predictable ways by RNA structure within the message [26]. In several plant viruses, a combination of non-AUG and poor-context AUG initiation codons allows production of three or even four functional proteins from a single transcript [27–29].

The 5’ end of sgRNA1 of luteoviruses and poleroviruses, where known, varies between 188 and 302 nt upstream of the ORF3 AUG [30–32], usually including sequence that encodes the 3’ end of ORF2. This is an unusually long leader sequence for a viral sgRNA because, as mentioned above, RNA viruses tend to minimize sequence length wherever possible. A long 5’ untranslated region (UTR) often implies the presence of a translational enhancer such as an internal ribosome entry site [33], but the cap-independent translation element for genus *Luteovirus*, called a BYDV-like translation enhancer (BTE), is located in the 3’ UTR [34, 35], and only a small stem-loop at the 5’ end of sgRNA1 is needed for cap-independent translation [36]. Instead of being a long 5’ UTR, we report here that the 5’ end of sgRNA1 of poleroviruses and luteoviruses (but not of the enamovirus) encodes a small ORF, termed ORF3a, that initiates at a non-AUG codon. The encoded protein P3a of TuYV is not required for replication in protoplasts but is required for systemic infection in plants.

## Results

### Computational analysis reveals a conserved ORF within the sgRNA1 leader

The coding potential of ORF3a was detected initially by applying the gene-finding program MLOGD to luteovirus and polerovirus sequence alignments. MLOGD uses nucleotide and amino acid substitution matrices to model sequence evolution in dual-coding, single-coding and non-coding regions [37]. It can be used to predict novel coding sequences via an approximate likelihood-ratio test. Although originally developed to analyze overlapping genes, MLOGD can also be used to analyze the coding potential in each of the three reading frames relative to a 'null' model in which the sequence is presumed to be non-coding in that reading frame.

Fig 1B illustrates the application of MLOGD to an alignment of 76 *Barley yellow dwarf virus* (BYDV) sequences (including serotypes PAV, PAS, MAV, GAV and the highly divergent Ker-II) with full or near-full genome coverage, using a 40-codon sliding window separately in each reading frame. A positive coding signature was observed in the correct reading frame throughout the ORF1, ORF2 and ORF5 regions. As expected, due to the lower number of substitutions in dual-coding regions, the coding signature was weaker in the ORF3/ORF4 overlap region, but still mainly positive. Furthermore, a positive coding signature was observed in the ORF6 region, supporting earlier evidence that it may encode a functional product [38–40]. Unexpectedly, a short region of positive coding potential was observed upstream of ORF3, in a region hitherto presumed to be part of the sgRNA1 non-coding leader (pink band, Fig 1B). Moreover, the region of positive coding potential coincided with a conserved absence of stop codons in the corresponding reading frame (Fig 1B). We named this open reading frame ORF3a. We observed that ORF3a is conserved in the three clades identified in the genus *Luteovirus*, (i) the BYDVs, (ii) *Bean leafroll virus*, *Soybean dwarf virus* and relatives, and (iii) *Rose spring dwarf-associated virus* (S1 Datafile).

We next analyzed 97 full- or nearly full-length genome sequences of viruses in genus *Polerovirus*. Recombination, particularly between the 5' replication and 3' capsid/movement gene blocks, is a common feature of polerovirus and luteovirus evolution [5, 41]. In view of this, the 5' ORF0-ORF1-ORF2 and 3' ORF3-ORF4-ORF5 gene blocks were extracted from polerovirus sequences and aligned separately. 178 nucleotides of 5' flanking sequence were included in the ORF3-ORF4-ORF5 alignment in order to include the potential ORF3a region and some upstream flanking sequence in the analysis. MLOGD revealed a positive coding signature in the correct reading frame throughout most of the ORF0, ORF1, ORF2, ORF3, ORF4 and ORF5 regions (Fig 1D). Again, a short but clear region of positive coding potential was observed just upstream of ORF3 and, once again, this coincided with a conserved absence of stop codons in the corresponding reading frame (pink band, Fig 1D). Note that this analysis does not provide information on any additional ORFs that may be restricted to just one or a few polerovirus species, such as the Rap1 ORF reported only in the PLRV genome [42].

### Translation of ORF3a is predicted to depend on non-AUG initiation

**Fig 2:**
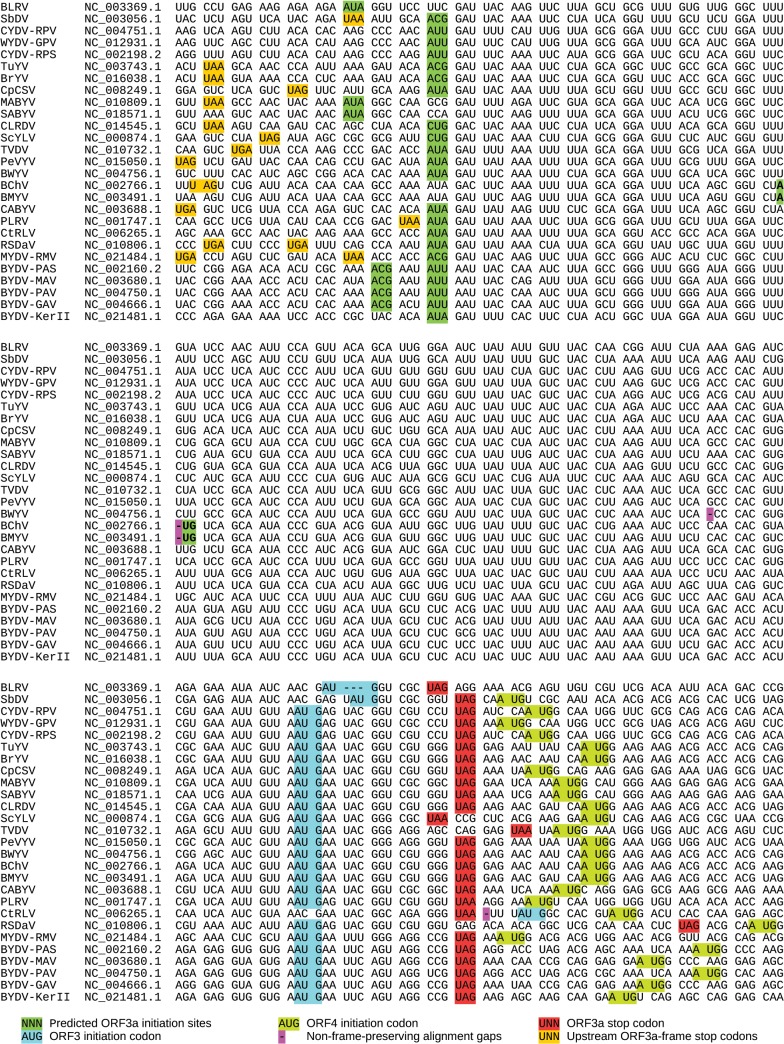
Sequence analysis of ORF3a and flanking regions. Pseudo-alignment of representative luteovirus and polerovirus NCBI species RefSeqs (GenBank accession numbers indicated at left) for ORF3a and flanking regions. Spaces separate codons in the ORF3a reading frame. The alignment within ORF3a is based on a P3a amino acid alignment (S1 Fig). The 5' and 3' flanking sequences have not been aligned since pan-genus alignments in these regions are ambiguous. Virus name abbreviations: BLRV—*Bean leafroll virus*; SbDV—*Soybean dwarf virus*; CYDV-RPV—*Cereal yellow dwarf virus-RPV*; WYDV-GPV—*Wheat yellow dwarf virus-GPV*; CYDV-RPS—*Cereal yellow dwarf virus-RPS*; TuYV—*Turnip yellows virus*; BrYV—*Brassica yellows virus*; CpCSV—*Chickpea chlorotic stunt virus*; MABYV—*Melon aphid-borne yellows virus*; SABYV—*Suakwa aphid-borne yellows virus*; CLRDV—*Cotton leafroll dwarf virus*; ScYLV—*Sugarcane yellow leaf virus*; TVDV—*Tobacco vein distorting virus*; PeVYV—*Pepper vein yellows virus*; BWYV—*Beet western yellows virus*; BChV—*Beet chlorosis virus*; BMYV—*Beet mild yellowing virus*; CABYV—*Cucurbit aphid-borne yellows virus*; PLRV—*Potato leafroll virus*; CtRLV—*Carrot red leaf virus*; RSDaV—*Rose spring dwarf-associated virus*; MYDV-RMV—*Maize yellow dwarf virus-RMV*; BYDV-PAS—*Barley yellow dwarf virus-PAS*; BYDV-MAV—*Barley yellow dwarf virus-MAV*; BYDV-PAV—*Barley yellow dwarf virus-PAV*; BYDV-GAV—*Barley yellow dwarf virus-GAV*; BYDV-KerII—*Barley yellow dwarf virus Ker-II*.

Although the presence of ORF3a is conserved throughout the *Luteovirus* and *Polerovirus* genera, in nearly all sequences it lacks a suitable AUG initiation codon. Thus we searched for potential non-AUG initiation codons. With few exceptions (see below), all available sequences with coverage of the ORF3a region contain a near-cognate non-AUG potential initiation codon, in a strong initiation context, near the 5' end of the maximal open reading frame (which is determined by the next upstream in-frame stop codon). Representative sequences (NCBI species RefSeqs) are shown in Fig 2; additional sequences are shown in S1 Datafile. The great majority of sequences contain an AUU, ACG, AUA or CUG ORF3a-frame codon (green shading, Fig 2), flanked by a favorable translation initiation context, i.e. an A at position -3 and frequently also a G at +4. Initiation at one of these codons would give rise to a 45–48 amino acid P3a product. The presence of ORF3a-frame stop codons (amber shading, Fig 2) in many sequences shortly upstream of this site further suggests that this is the site of initiation. As only a fraction of scanning 40S ribosomal subunits initiate translation at any given non-AUG initiation codon, it is possible that in some species multiple non-AUG initiation sites are utilized (e.g. closely spaced ACG and AUU codons, both with an A at -3, in BYDV sequences NC_002160, NC_003680, NC_004750 and NC_004666; Fig 2). ORF3a normally terminates shortly downstream of the ORF3 initiation codon, overlapping ORF3 in the +2 reading frame (Fig 2). ORF4 overlaps ORF3 in the +1 reading frame and, in almost all viruses, the ORF4 initiation codon is shortly downstream of the ORF3a termination codon (Fig 2).

Four of the 27 NCBI RefSeqs differ from this general pattern. Uniquely, in one luteovirus (NC_006265, *Carrot red leaf virus*) the ORF3a stop codon is upstream of the ORF3 AUG codon. This is due to replacement of the canonical ORF3 AUG codon with ACG (Fig 2). The single nucleotide deletion that disrupts the ORF3a reading frame in NC_004756 (*Beet western yellows virus*; pink '-' in Fig 2) is not present (i.e. is replaced with a nucleotide) in all other available *Beet western yellows virus* sequences in NCBI (>30 sequences) suggesting that the RefSeq may have a sequencing error or represent a defective genome. In NC_003491 (*Beet mild yellowing virus*, BMYV) and NC_002766 (*Beet chlorosis virus*), ORF3a is shorter and initiates with an AUG codon in a weak context instead of a non-AUG initiation codon in a strong context (Fig 2). However, in other sequences of these two species the AUG is replaced with AUUG giving rise to the full-length canonical ORF3a (see S1 Datafile). Whether these RefSeqs represent defective sequences or functional variants remains to be determined. However, an infectious clone of BMYV contains an intact ORF3a with AUUG rather than the AUG sequence present in the RefSeq [43].

We suggest that these exceptions are due to sequencing errors or sequences of nonviable viral RNAs. It should be noted that NCBI RefSeqs are often derived from older sequences (typically the first full-length sequence obtained for a species) and are sometimes prone to sequencing errors that are not supported by later sequencing (e.g. see S1 Datafile). While insertion/deletion errors that occur in known coding ORFs are generally corrected, errors elsewhere often escape notice. The long standing confusion regarding the genome organization of sobemoviruses, which arose as a result of insertion/deletion errors in a number of early sequences, is a case in point [44]. When we analyzed all 459 sequences available in GenBank with coverage of the ORF3/3a region, only 10 were found to be defective or potentially defective with respect to ORF3a as a result of insertions, deletions or premature termination codons, while only one sequence lacked a strong initiation context at the canonical ORF3a initiation site (S1 Datafile).

The protein product of ORF3a, P3a, has a predicted molecular mass normally in the range 4.8 to 5.3 kDa. Its amino acid sequence is generally highly conserved between divergent virus species (S1 Fig). Moreover, all sequences, except the two which are N-terminally truncated (AUG initiation; see above), contain a predicted transmembrane region towards the N-terminus of P3a (S1 Fig).

### ORF3a is translated *in vitro*

In order to experimentally evaluate the expression of ORF3a, we selected the polerovirus *Turnip yellows virus* (TuYV), and performed *in vitro* translation experiments in wheat germ extracts using T7-derived transcripts starting at nt 3259 which corresponds to the 5’ end of TuYV sgRNA1. This places ORF3a in its natural context in the TuYV sgRNA1, beginning upstream of ORFs 3 and 4. Based on alignments, translation of ORF3a is suspected to start with an ACG (Fig 2, NC_003743.1, nt 3365) and to stop with a UAG (nt 3502), producing a theoretical protein of 45 amino acids (MW 5.1 kDa). This ACG displays a favorable translation initiation context (A at -3 and G at +4) at a position that is conserved among most poleroviruses and luteoviruses (Fig 2; S1 Datafile).

**Fig 3:**
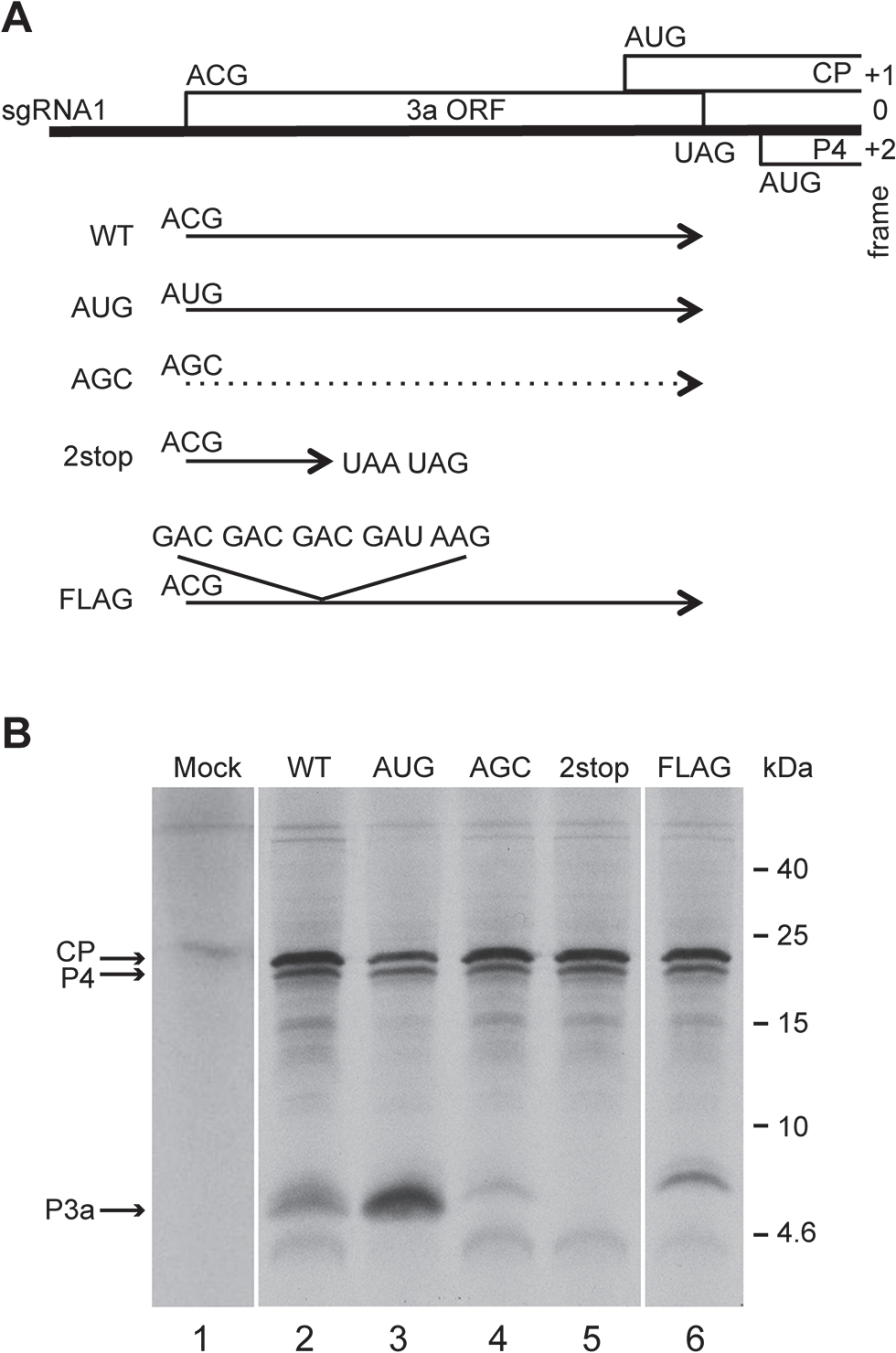
Schematic representation of the TuYV-3a mutants and *in vitro* translation of their corresponding subgenomic RNA1. **A.** Mutations introduced into ORF3a: TuYV-3aAUG, TuYV-3aAGC, TuYV-3a2stop and TuYV-3aFLAG. The substitutions or insertions are indicated for each mutant. The translational potential of the different constructs regarding ORF3a is shown by a plain arrow starting at the ACG or modified codon at the same position. The dotted arrow indicates an expected absence of translation from the AGC codon. **B.** Tricine-SDS-PAGE analysis of proteins translated in wheat germ extracts from the indicated sgRNA1 mutants transcribed *in vitro*. Mock: no RNA added. After drying the gel, the radioactively labeled proteins were detected by phosphorimager. Positions of CP, P4 and putative P3a are indicated on the left. Sizes of molecular weight markers are indicated on the right.

To monitor ORF3a expression and function, several mutants were constructed (Figs 3A and S2). As a positive control, the putative ORF3a start codon (ACG) was mutated to AUG (mutant “AUG”). As a negative control, the ACG was mutated to AGC, a codon that should not function as a translation initiation site [1, 45]. Capped *in vitro* transcripts of the corresponding subgenomic RNAs (WT, AUG and AGC) were translated in wheat germ extracts and a band migrating at about 6.8 kDa was generated from the WT and AUG constructs (Fig 3B). Although migrating more slowly than the predicted size of 5.1 kDa, evidence below supports the notion that this is the product of ORF3a, initiating at the predicted ACG codon. For example, as predicted, translation of this protein increased when the ACG was changed to AUG (Fig 3B, lanes 2 and 3). Moreover, no band of similar size was observed from the construct in which ACG was mutated to AGC (Fig 3B, lane 4), but two minor bands (migrating at 4.6 and 7.3 kDa), also present with the WT construct, were observed. These bands could result from alternative translation initiation events, with the 7.3 kDa-migrating product potentially arising via initiation at an in-frame AUU codon four triplets upstream of the ACG (S2 Fig). The lower mass product of 4.6 kDa could result from initiation at one of several in-frame downstream alternative initiation codons (AUA, GUG and AUC), the first one being located 16 codons downstream of the ACG (Figs 2 and S2). To verify that the higher mass proteins arose from ORF3a, tandem stop codons (UAA UAG) were introduced by site-directed mutagenesis of two internal codons (UCA UCG, 14 codons downstream of the proposed ACG initiation codon) in order to prematurely interrupt translation of ORF3a (Figs 3A, 2stop construct, and S2). Translation of the corresponding sgRNA1 yielded neither the main 6.8 kDa protein nor the minor 7.3 kDa band (Fig 3B, lane 5). This observation confirms that translation of both products initiated at codons upstream of, and in-frame with, the introduced stop codons, which is in agreement with a major translation initiation at the aforesaid ACG codon (nt 3365) generating the P3a protein.

In a first attempt to detect the P3a protein *in vivo* (see below), a tagged version was constructed with a FLAG tag (DYKDDDDK) positioned directly after the P3a ACG initiation codon, by adding five codons (DDDDK) to the DYK encoded in the WT TuYV ORF3a (Figs 3A and S2, FLAG mutant). Translation of the FLAG construct generated a band of a slightly larger size (8 kDa) than the P3a protein expressed from the WT construct, with an additional faint band above it, supporting the ACG as the major initiation codon of ORF3a, and an upstream codon (presumably the aforementioned AUU) being used as an alternative initiation site (Figs 3B, lane 6, and S2). All together, these experiments showed that ORF3a can be translated *in vitro* in wheat germ extracts from a synthetic subgenomic RNA to produce a protein of 6.8 kDa apparent MW.

Translation of the wild type and mutant subgenomic RNAs also produced major bands corresponding to the P4 and the CP proteins (19.5 kDa and 22.5 kDa, respectively) (Fig 3B). The ORF3a AUG mutation reduced accumulation of the 22 kDa product when compared with the WT and the other constructs (Figs 3B and S3). No significant variation in the accumulation of either protein was noticed with any of the other mutants.

### The P3a protein is expressed, but not required for virus replication in protoplasts

**Fig 4:**
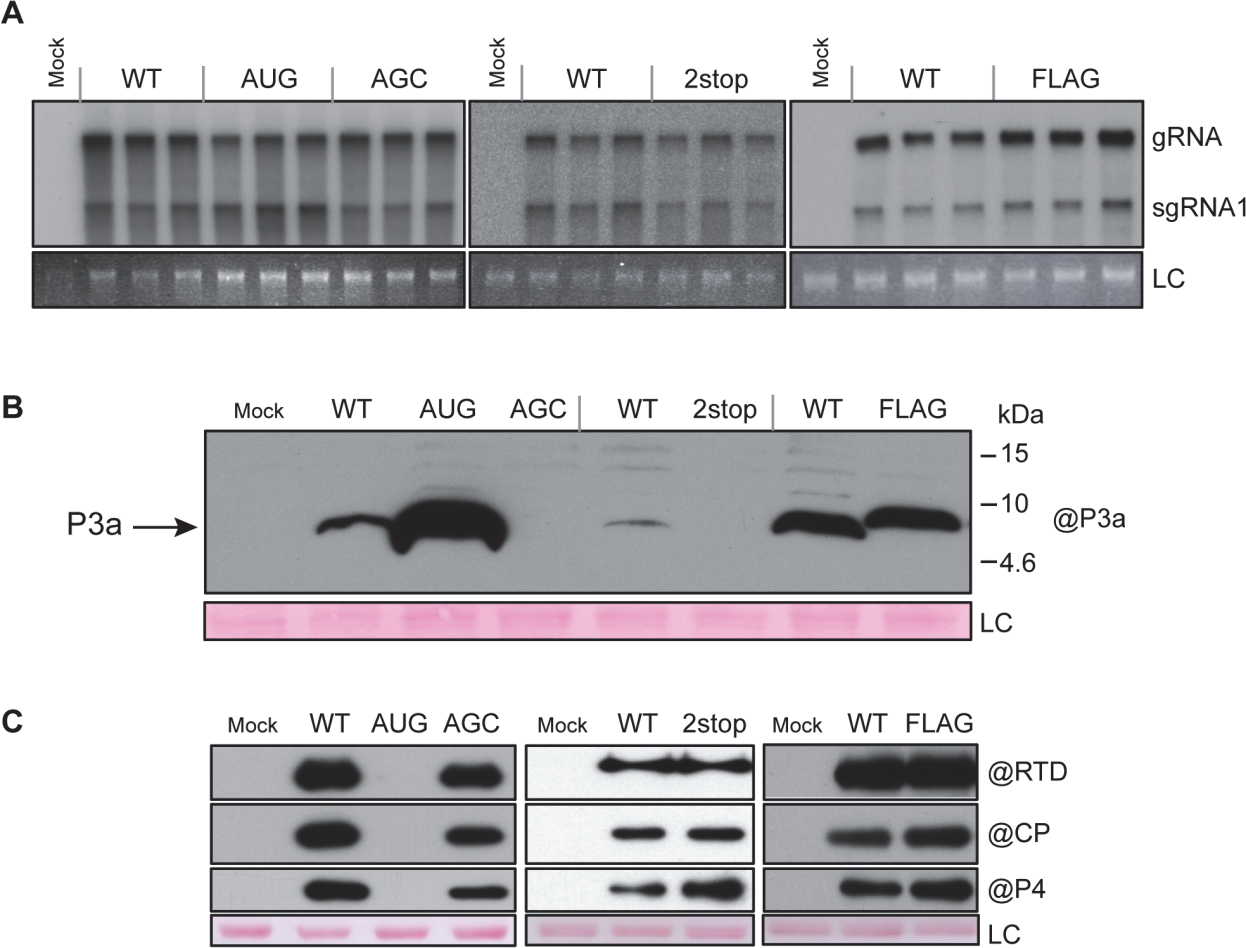
Protoplast infection with TuYV-ORF3a mutants. **A.** Northern blot analysis of RNA extracted from triplicate samples of *C*. *quinoa* protoplasts inoculated with *in vitro* transcribed genomic RNAs corresponding to the wild type TuYV (WT) or the TuYV-3aAUG (AUG), TuYV-3aAGC (AGC), TuYV-3a2stop (2stop) or TuYV-3aFLAG (FLAG) mutants. Mock, mock-inoculated control; gRNA, genomic RNA; sgRNA1, subgenomic RNA1; LC, loading control, consisting of ethidium bromide-stained ribosomal RNA. Blots indicate results of three separate experiments, each with its own negative (Mock) and positive (WT) control. **B.** and **C**. Western blot analyses of proteins extracted from the same infected protoplast samples as in panel A. The blots were incubated with antibody specific to P3a (B), or CP, P4 or RTD protein (C). The molecular weights and mobilities of prestained protein markers are indicated in B. LC, loading control of proteins stained on the membranes by Ponceau red.

To determine whether ORF3a is expressed during infection and whether it plays a role in viral replication, the previously described mutations were introduced into the T7-based TuYV full-length clone (pTuYV-WT, formerly named pBW_0_, [46]). Capped *in vitro* transcripts derived from the mutated viral clones pTuYV-3aAUG, -3aAGC, -3a2stop and -3aFLAG mutants were inoculated to *Chenopodium quinoa* protoplasts. At 44 hours post-inoculation (hpi), northern blot hybridization revealed that all four mutants produced genomic (gRNA) and the major subgenomic RNA (sgRNA1) at levels similar to TuYV-WT (Fig 4A). Thus viral RNA replication is independent of the presence of P3a.

Expression of ORF3a *in vivo* was analyzed by western blot using specific antibodies generated against the fifteen C-terminal amino acids of P3a. The P3a protein was detected with an apparent MW of 6.5 kDa in protoplasts infected with TuYV-WT or the TuYV-3aAUG mutant and 8.5 kDa in TuYV-3aFLAG-infected protoplasts (Fig 4B). TuYV-3aAUG yielded much more P3a protein than the WT virus, as already observed in the *in vitro* translation experiments (see above). Conversely, and as expected, no P3a protein was detected in protoplasts infected with the null-3a mutants TuYV-3aAGC or TuYV-3a2stop (Fig 4B). No other major P3a-related products were detected in protoplast extracts suggesting that the 7.3 kDa and 4.6 kDa products generated in cell-free translation (Fig 3B) were due to aberrant translation initiation that does not occur *in vivo*. The FLAG-tagged P3a was also detected using commercial antibodies against the FLAG epitope (S4 Fig). These results indicate unambiguously that the P3a protein is expressed *in vivo* during viral infection.

To investigate the effect of ORF3a on expression of the other proteins produced from the sgRNA1 in infected cells, CP-RTD, CP and P4 protein accumulation was analyzed by western blotting of proteins extracted from infected protoplasts. All mutants, except TuYV-3aAUG, produced amounts of CP, P4 and CP-RTD proteins similar to those of TuYV-WT (Fig 4C). In contrast, accumulation of CP, P4 and CP-RTD from the TuYV-3aAUG mutant was drastically impaired and not visible on the blot in Fig 4C. Loading increasing amounts of TuYV-3aAUG-infected protoplasts revealed a band corresponding to less than one-tenth of the amount of CP-RTD present in TuYV-WT-infected cells (S5 Fig). No CP could be detected in 30,000 protoplasts, while as few as 3,000 cells allowed CP detection in TuYV-WT-inoculated protoplasts (S5 Fig). Thus, the CP-RTD protein appears to accumulate in higher levels relative to CP in TuYV-3aAUG-infected protoplasts than in TuYV- WT-inoculated cells. Alternatively, the CP antibodies may have a nonlinear response to dilution and are unable to detect CP below a certain threshold at which the CP-RTD-specific antibodies can still detect CP-RTD. To conclude, while absence of P3a expression had little effect on expression of the other sgRNA1-encoded proteins (CP, P4, CP-RTD), overexpression of P3a from a strong initiation codon inhibited expression from downstream AUG codons much more strongly than in wheat germ extract.

### Analysis of the P3a mutants in inoculated leaves

**Fig 5:**
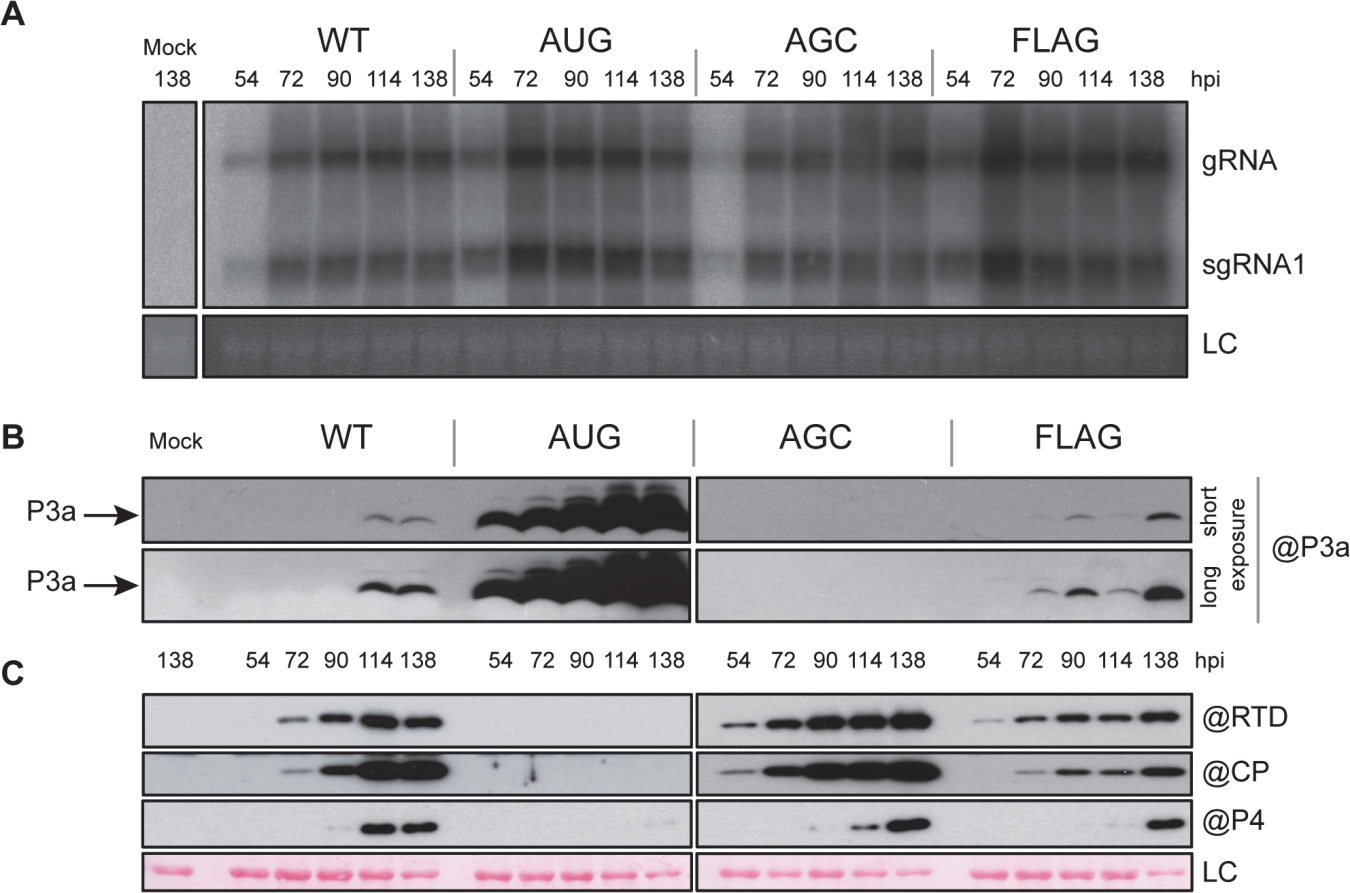
Infection of *A*. *thaliana* infiltrated leaves. **A.** Northern blot hybridization of RNA extracted from *A*. *thaliana* leaves agroinfiltrated with the wild-type TuYV (WT) or the mutants TuYV-3aAUG (AUG), TuYV-3aAGC (AGC), or TuYV-3aFLAG (FLAG). Mock, mock-inoculated control; gRNA, genomic RNA; sgRNA1, subgenomic RNA1, LC, loading control of ethidium bromide stained ribosomal RNA. Each sample corresponds to a mixture of leaves from 3 individual plants. **B.** and **C**. Western blot analysis of proteins extracted from the same infected leaf samples. Proteins were blotted on the same membrane and incubated with antibody (@) specific to P3a (B), CP, P4 and RTD protein (C). The blots were then cut in two for the figure. Two different exposures (short and long) are shown for P3a detection. LC, loading control of proteins stained on the membranes by Ponceau red.

To explore the role of the P3a protein in the viral infection process *in planta*, we first analyzed the outcome of infection with the TuYV-3a mutants in inoculated leaves of *Arabidopsis thaliana*. Full-length viral cDNAs containing the different mutations, and driven by the *Cauliflower mosaic virus* (CaMV) 35S promoter, were agroinfiltrated into *A*. *thaliana* leaves. Total RNA was analyzed by northern blot hybridization. To minimize sample variation, agroinfiltrated leaves from three different plants were collected for each time point. Viral RNAs (gRNA and sgRNA1) were detected in TuYV-WT-inoculated leaves at 54 hpi and accumulation reached a maximum at 72 hpi and remained at that level throughout the 138 hour experiment (Fig 5A). Both the P3a-overexpressing TuYV-3aAUG mutant and the null TuYV-3aAGC and TuYV-3a2stop mutants displayed similar replication kinetics to wild type (Fig 5A). Western blot analysis of TuYV-WT-infected samples detected P3a at 114 hpi (Fig 5B). P3a was detected as early as 54 hpi in TuYV-3aAUG-infected leaves and accumulated to much higher levels than in TuYV-WT-infected leaves. The tagged P3a protein from the TuYV-3aFLAG mutant was also detected earlier and accumulated to slightly higher levels (72 hpi) compared to TuYV-WT (Fig 5B). However this observation might be related to a higher epitope accessibility of P3aFLAG compared with the wild type protein. As expected, no P3a was detected in the TuYV-3aAGC-infected leaves (Fig 5B).

We also examined the accumulation levels of the CP, CP-RTD and P4 proteins in the samples inoculated with the different ORF3a mutants. All three proteins were detected by 90 hpi in TuYV-WT-infiltrated leaves (Fig 5C). Leaves infiltrated with the TuYV-3aAUG mutant reproducibly contained no detectable CP, CP-RTD or P4 at any time, even with longer blot exposure (Fig 5B). Conversely, in TuYV-3aAGC-infiltrated leaves, CP and CP-RTD accumulated in higher amounts at early time points compared to the levels in TuYV-WT-infiltrated leaves (Fig 5C). Expression of CP, CP-RTD and P4 was reduced in TuYV-3aFLAG-infiltrated leaves (Fig 5C). Thus, the ORF3a mutations not only affect P3a synthesis, but they also significantly alter accumulation of the other proteins encoded by sgRNA1.

### The P3a protein is required for efficient systemic infection of plants

**Fig 6:**
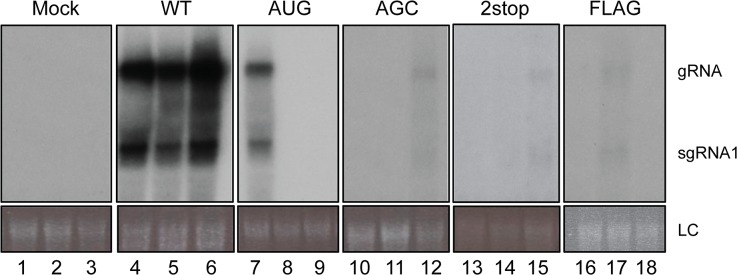
Systemic infection of *A. thaliana*. Northern blot analysis of RNA extracted from upper, non-inoculated leaves of *A*. *thaliana* plants infiltrated with the wild-type TuYV (WT) or one of the TuYV-3a mutants, TuYV-3aAUG (AUG), TuYV-3aAGC (AGC), TuYV-3a2stop (2stop) or TuYV-3aFLAG (FLAG). All plants were analyzed but only three samples representative for the TuYV-WT and each mutant are shown, with at least one sample of an infected plant, confirmed by RT-PCR. Mock, mock-inoculated control; gRNA, genomic RNA; sgRNA1, subgenomic RNA1, LC, loading control of ethidium bromide stained ribosomal RNA.

Systemic infection was investigated by analyzing the presence of viral RNA in upper non-inoculated leaves of *A*. *thaliana* plants that had been agroinfiltrated with TuYV-WT or with one of the TuYV-3a mutants. While the efficiency of TuYV-WT systemic infection was 92%, all the TuYV-3a mutants (whether over-expressor TuYV-3aAUG, knock-out TuYV-3aAGC and TuYV-3a2stop, or tagged TuYV-3aFLAG) were poorly infectious as shown by the low percentage of infected plants (Table 1). Moreover, accumulation of the viral RNAs in these plants was very low especially for the mutants TuYV-3aAGC, TuYV-3a2stop and TuYV-3aFLAG as shown by northern blot hybridization with one positive sample for each mutant (Fig 6, lanes 12, 15, 17). The stability of the engineered mutations in the viral progeny was investigated by RT-PCR and sequencing. In the two plants out of 29 that became infected with TuYV-3aAUG (e.g. Fig 6, lane 7), the initial AUG mutation had reverted to the WT ACG sequence. No other modifications were found in the entire subgenomic sequence. In the progeny of the three other mutants, TuYV-3aAGC, TuYV-3a2stop and TuYV-3aFLAG, no base changes were observed (3 plants analyzed for TuYV-3aAGC, 2 plants for TuYV-3a2stop and 5 for TuYV-3aFLAG) (Table 1), showing that the modifications introduced in the viral sequence of these three mutants were maintained through viral replication.

**Table 1:** Systemic infection by TuYV containing P3a mutants.

Virus	Systemically infected plants/ total inoculated plants^a^	Mutation outcome^b^
TuYV-WT	67/73 (92%)	WT
TuYV-3aAUG	2/29 (7%)	reverted to WT
TuYV-3aAGC	3*/32 (9%)	conserved
TuYV-3a2stop	2*/32 (6%)	conserved
TuYV-3aFLAG	7*/26 (27%)	conserved

^**a**^Infection was detected by northern blot hybridization of viral RNA from non-inoculated leaves and confirmed by RT-PCR.

^**b**^The outcome of the mutation was analyzed by sequencing the entire subgenomic RNA of progeny virus isolated from non-inoculated leaves.

*Very low level of RNA accumulation in non-inoculated leaves.

**Fig 7:**
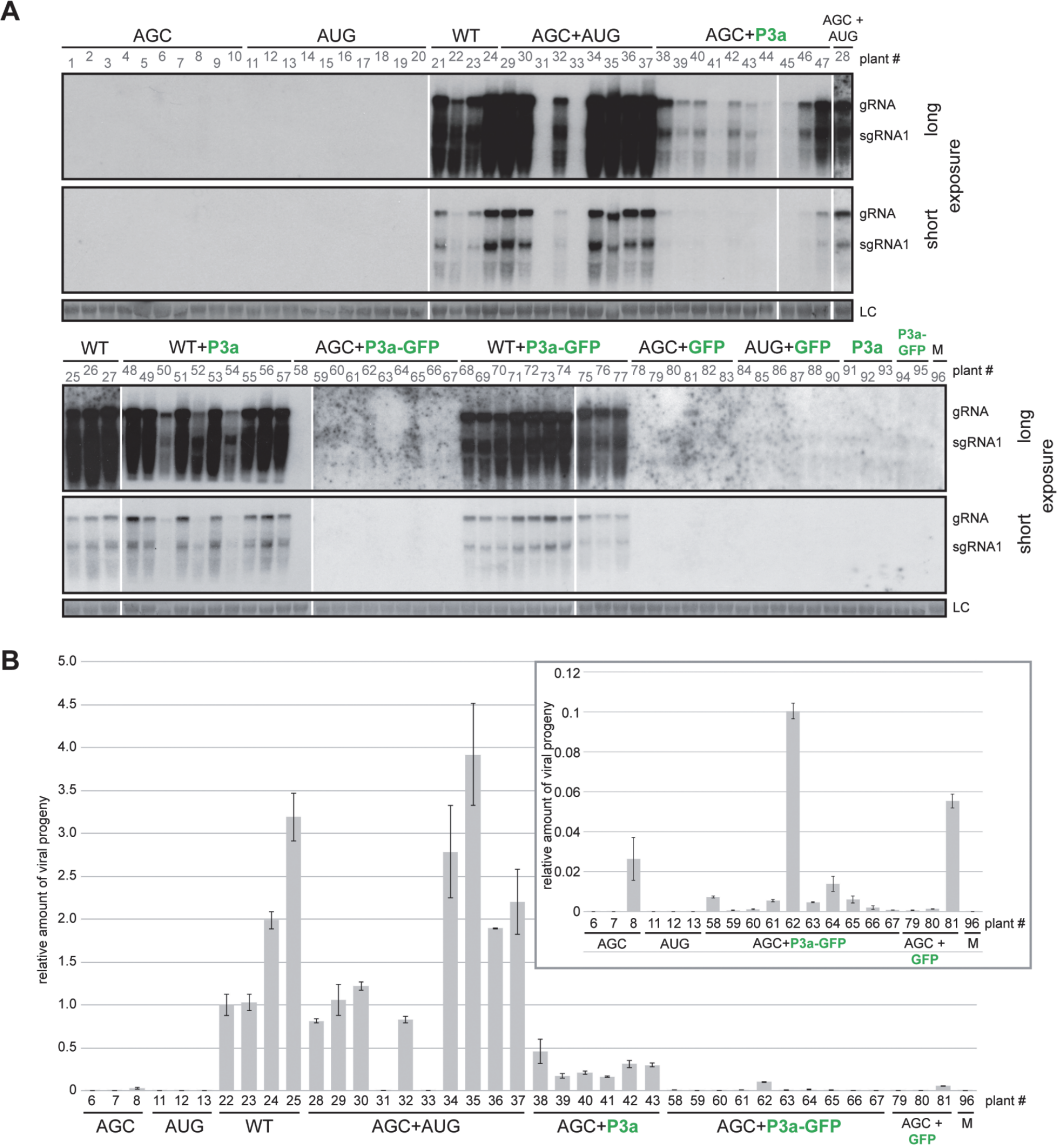
Complementation analysis of ORF3a mutants. **A.** Northern blot hybridizations of RNA extracted 21 d.p.i. from *N*. *benthamiana* systemic leaves co-infiltrated with the TuYV-3aAGC mutant (AGC) or the TuYV-WT virus (WT), and either the TuYV-3aAUG mutant (AUG) or agrobacterium transiently expressing the P3a, P3a-GFP or GFP protein. Viral mutants are labeled in black; transiently expressed proteins are labeled in green. Controls were conducted with infiltrations of TuYV-3aAGC (AGC), TuYV-3aAUG (AUG), TuYV-WT (WT), the empty vector (M) or agrobacterium expressing either P3a or P3a-GFP or co-infiltrations of TuYV-3aAUG (AUG) and agrobacterium expressing GFP. No additional suppressor of RNA silencing was supplied. Due to the number of samples, the blots are presented in two parts and for each of them a long and a short exposure is shown. **B.** Quantification of TuYV RNA by qRT-PCR in systemic leaves of *N*. *benthamiana* agroinfiltrated or co-agroinfiltrated with the mutants and agrobacterium expressing P3a, P3a-GFP or GFP as shown in panel A. The number of the samples refers to the same plants as those shown in panel A. After normalization with GAPDH reference gene, the values were normalized arbitrarily with sample #22. The insert is a magnification of the values of some samples that were particularly low.

We hypothesize that lack of systemic movement of TuYV-3aAGC is due to absence of P3a protein, and that lack of systemic movement of TuYV-3aAUG is due to insufficient translation of the downstream ORFs encoding the proteins CP, CP-RTD and P4. If this is the case, then the two mutant viruses may be able to complement each other to facilitate systemic infection. As we intended to investigate in parallel direct complementation by a P3a-expressing vector that had to be carried out in *N*. *benthamiana*, we chose this plant as a common host for both complementation experiments. Ten plants inoculated with TuYV-3aAGC alone triggered local infection but did not develop systemic infection with one exception, which had an extremely low level of viral RNA (Fig 7B, plant #8). This shows that P3a is required for long distance movement of TuYV in *N*. *benthamiana*, as well as *A*. *thaliana*. Similarly TuYV-3aAUG was able to accumulate only in infiltrated leaves however none of the 10 plants inoculated with TuYV-3aAUG showed viral spread in upper leaves (Fig 7, plants 11–20), as was also observed in *A*. *thaliana*. When *N*. *benthamiana* plants were co-infiltrated with TuYV-3aAGC and TuYV-3aAUG, eight out of ten co-infiltrated plants showed wild type levels of viral RNA accumulation in the non-inoculated leaves at 21 d.p.i. as shown by northern blot hybridization and real-time RT-PCR (Fig 7, plant numbers #28–37).

In order to identify the nature of the progeny viral genomes that moved systemically and multiplied in the upper leaves, we performed sequence-specific qRT-PCR, using primers designed to detect only WT, only TuYV-3aAUG, or only TuYV-3aAGC mutants (S6 Fig). The WT-specific primer indeed detected only WT RNA in the WT-inoculated plants (four plants tested #22–25) and gave no amplification of RNA in the eight systemically infected plants co-inoculated with TuYV-3aAGC and TuYV-3aAUG (S6 Fig, plants #28–37). Moreover, the TuYV-3aAGC and TuYV-3aAUG primers detected viral RNA only in plants inoculated with those mutants and not in the WT-infected plants. These quantifications were first normalized with a reference gene (GAPDH) whose expression was shown to remain stable upon various viral infections [47] before being normalized with the sample’s value obtained with the common set of primers and finally normalized with a positive sample (taken arbitrarily). Therefore the relative values shown in S6 Fig reflect only presence or absence of the corresponding virus and do not allow comparison of levels of one mutant versus another. The ratio of the two mutant viruses present in the systemic leaves of the co-inoculated plants could be estimated by comparing the cycle threshold (Ct) values of the samples to a calibration curve generated with the corresponding plasmid. A mean value of 2.4 was obtained for the ratio TuYV-3aAGC/TuYV-3aAUG (extreme values: 1.7–3.1). The RT-PCR results revealed that the mutations were maintained, and that the mutants did not revert to wild type. Recombination was not an issue as the mutations changed the same nucleotides (S6A Fig). Thus, both mutant viruses, each individually incapable of systemic infection, can complement each other to move systemically as efficiently as WT virus.

To determine whether the above complementation of TuYV-3aAGC by TuYV-3aAUG is due to the provision of P3a by the latter virus, we tested whether expression of P3a alone is capable of complementing TuYV-3aAGC. Indeed, co-infiltration of leaves with agrobacteria expressing TuYV-3aAGC and agrobacteria containing a plasmid that expresses only P3a driven by the CaMV 35S promoter, yielded significant levels of TuYV-3aAGC RNA in systemic leaves of 10 out of 10 plants at 21 d.p.i. (Fig 7). The RNA levels were generally less than those in the plants complemented with TuYV-3aAUG, but consistently and significantly above levels in systemic leaves of plants inoculated with TuYV-3aAGC alone (Fig 7, plants #38–44). Plasmid expressing P3a with a C-terminally fused green fluorescent protein (P3a-GFP) did not efficiently complement TuYV-3aAGC, except for one plant out of 10 (Fig 7, plant #62). Nevertheless most plants were positive by qRT-PCR, albeit at very low levels (Fig 7B see insert; compare plants #58–67 with mock-inoculated plant #96), suggesting that the GFP fusion reduced function or expression of P3a to levels that did not permit efficient complementation. Transient expression of P3a and P3a-GFP in presence or absence of the viral mutant TuYV-3aAGC was confirmed in infiltrated leaves by western blotting using specific antibodies (S7 Fig). Curiously, as observed by northern blot analysis in *A*. *thaliana* plants inoculated with TuYV-3aAGC (Table 1), one plant infiltrated with TuYV-3aAGC alone and one infiltrated with TuYV-3aAGC plus GFP gave positive but weak signals by qRT-PCR analysis in *N*. *benthamiana* (Fig 7B, plants # 8 and 81), suggesting the potential for rare escape events. Overall, the data provided in this work strongly support the notion that P3a is necessary for viral systemic infection and that it can facilitate long distance movement when provided *in trans*.

**Fig 8:**
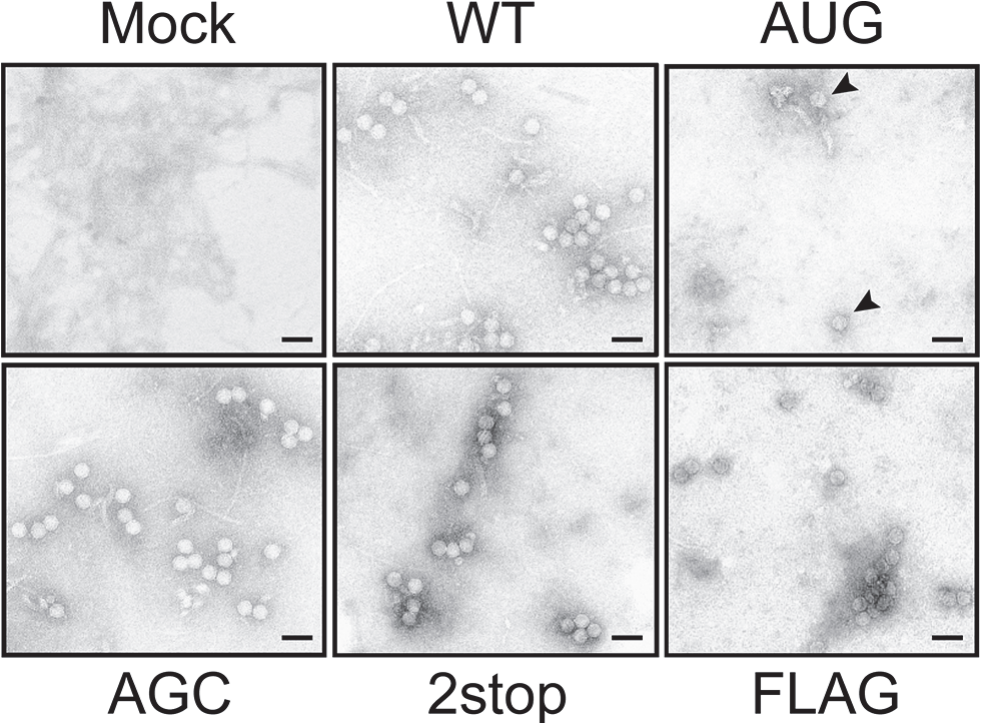
Viral particles produced by TuYV-3a mutants. Negatively stained viral particles in *A*. *thaliana* inoculated with TuYV-WT, TuYV-3aAUG, TuYV-3aAGC, TuYV-3a2stop and TuYV-3aFLAG. Virus purification was performed from leaves agroinfiltrated with the viral constructs. Viral particles were observed by transmission electron microscopy (TEM). A TuYV polyclonal antiserum was used to capture viral particles on the grids before TEM. Mock: purified sample from leaves agroinfiltrated with an empty pBin plasmid. Scale bar: 50 = nm.

Because virion formation is a prerequisite to TuYV long-distance movement [48], we investigated the ability of the TuYV-3a mutants to form particles. Immunosorbent electron microscopy (ISEM) performed on purified viral preparations from leaves agroinfiltrated with TuYV-3aAGC, TuYV-3a2stop and TuYV-3aFLAG mutants revealed typical virus particles which did not differ in conformation from WT virions (Fig 8). A few particles were detected on grids from the TuYV-3aAUG mutant. Interestingly, in protoplast infections where only one replication cycle occurs, no particles were observed for this mutant while particles were easily detected for the other mutants (S8 Fig). This suggests that the particles found in TuYV-3aAUG-inoculated leaves may be due to rare reversion events as described earlier (Table 1 and Fig 6); Therefore, the inability of both TuYV-3a knockout mutants to move efficiently to non-inoculated leaves of agroinfected plants cannot be attributed to the absence of capsid formation but rather to the inhibition of another step required for viral long-distance spread. This conclusion is reinforced by the ability of P3a to complement movement of the TuYV-3aAGC mutant. Taken together, these results show that the P3a protein plays a crucial role in systemic infection.

### P3a localizes to Golgi and in close proximity to plasmodesmata

**Fig 9:**
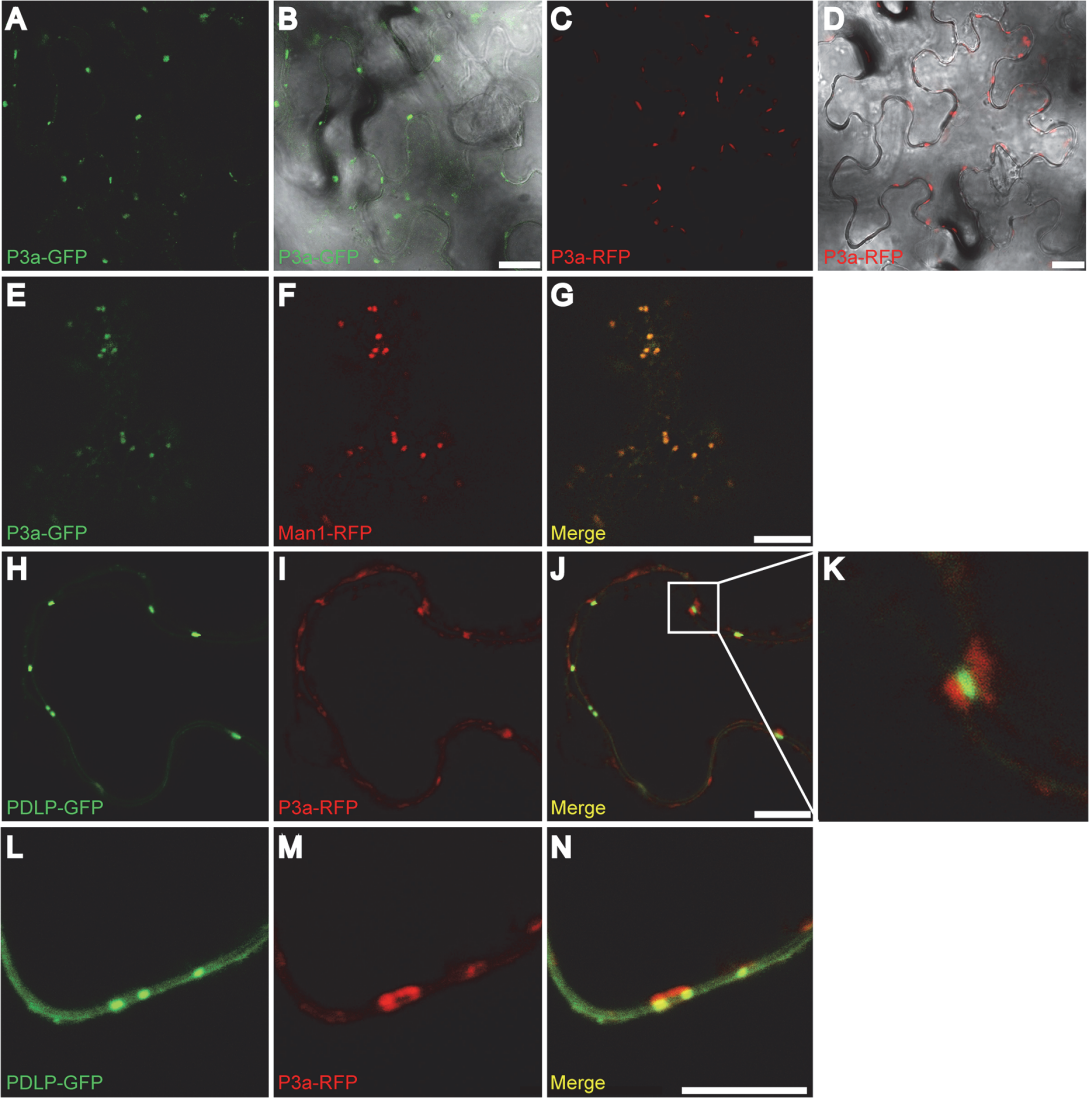
Subcellular localization of P3a. GFP- or RFP-fusion proteins were transiently expressed in *N*. *benthamiana* leaves and epidermal cells were observed under confocal microscopy. (A-B) Expression of P3a-GFP; (C-D) of P3a-RFP. (E-G) Co-expression of P3a-GFP (E) with Man1-RFP (a Golgi apparatus marker, panel F) and the merged fluorescent signal (G). (H-N) Co-expression of P3a-RFP (I and M) with the plasmodesmata marker PDLP1-GFP (H and L) and the merged fluorescent signal (panels J, K and N). A close-up of the box drawn in J is shown in panel K. Panels L-N show co-localization of the same proteins at a higher magnification. Scale bars, 20 μm for panels A-D and 10 μm for panels E-J and L-N. At least two independent experiments were performed for each condition. Representative images of the observations are presented. Western blot analyses of tissues expressing P3a-GFP and P3a-RFP fusions with GFP- and RFP-specific antibodies showed that the fusion proteins migrated in their monomeric form, with no major breakdown products released (S8 Fig).

To further address the role of the P3a protein in viral infection, its subcellular localization was observed in epidermal cells of *Nicotiana benthamiana*. Because P3a contains a putative trans-membrane domain near its N-terminus, whose function might be affected by the fusion with a bulky marker, ORF3a was fused at its 3’ end to a GFP or RFP ORF and expressed under the CaMV 35S promoter. When agroinfiltrated into *N*. *benthamiana* leaves, both constructs expressed fusion proteins of the expected size (S9 Fig). Both P3a-GFP and P3a-RFP proteins visualized by confocal laser scanning microscopy showed cytoplasmic punctuate structures (Fig 9A–9D). Co-expression of P3a-GFP and a *cis*-Golgi marker, α-1,2 mannosidase-1 fused to RFP (Man1-RFP) [49] showed a perfect co-localization of P3a-GFP with Man1-RFP (Fig 9E–9G), suggesting that P3a is associated with individual Golgi bodies. Fluorescent spots were also observed at discrete areas near the cell wall of epidermal cells. To pinpoint the locations of these spots, we co-expressed the P3a-RFP protein with a plasmodesmata marker (plasmodesmata-localized protein-1; PDLP-1) fused to GFP [50] (Fig 9H–9N). Whereas P3a-RFP localized near plasmodesmata, precise co-localization with the PDLP-1-GFP marker was not observed. Higher magnification views of some spots showed that P3a-RFP was adjacent to plasmodesmata, and appeared to remain essentially outside of the cell wall (Fig 9K–9N). To confirm this specific position of P3a, the leaf discs infiltrated with the construct P3a-RFP were stained with aniline blue, a callose marker. Callose is known to be deposited at the neck region of plasmodesmata [51]. Blue staining of callose was observed at potential positions of plasmodesmata in the cell wall while the P3a protein was consistently observed close to the labeled callose but not merged with it (S10 Fig).

## Discussion

### Translational control

By applying bioinformatics tools to genome sequences of luteoviruses and poleroviruses, we have discovered a previously overlooked essential gene, ORF3a, that is conserved throughout the *Luteovirus* and *Polerovirus* genera. Translation of ORF3a depends on non-AUG initiation on the sgRNA. Thus, via additional leaky scanning and stop codon readthrough, four distinct proteins (P3a, CP, P4 and CP-RTD) are expressed from a single sgRNA species. This work adds to the increasing known prevalence of non-AUG codons as start codons. While the use of ACG, AUA, AUU and CUG as weak start codons has been known for some time, including in plants [25] and viruses [52–54], this was thought to be a rarity, until many more were revealed by ribosome profiling and bioinformatics approaches [55–57]. This opens up a vast increase of potential coding capacity in viruses and host mRNAs for proteins needed only in small quantities [58].

In addition to containing ORF3a, the sequence of the sgRNA1 5’ end plays a key role in cap-independent translation of BYDV and other viruses in genus *Luteovirus*. These viruses contain a BYDV-like cap-independent translation element (BTE) in the 3’ UTR which must base pair to a stem-loop at the 5’ end of the mRNA [35] (upstream of ORF3a in sgRNA1) to facilitate translation initiation. In competitive conditions, sgRNA1 of BYDV translates more efficiently than the full-length genomic RNA, and this efficiency is conferred by what was thought to be the 5’ UTR, including ORF3a [59]. This differential translation efficiency was proposed to be due to the relative lack of secondary structure in the sgRNA1 5’ end, but the role of ORF3a in this preferential translation is unknown. This role for the 5’-terminal sequence of sgRNA1 in translation may apply only to genus *Luteovirus*, because poleroviruses are not known to harbor a 3’ cap-independent translation element. Truncation of the 5’UTR of the PLRV sgRNA1 was reported to increase translation efficiency of CP and P4 [60], an effect that is likely due to the absence of ORF3a and not solely to the shorter 5’ end *per se*.

Knock-out of ORF3a did not evidently alter the expression level of CP, P4 and CP-RTD in protoplast infections. In leaves infected with the AGC knock-out mutant, the CP and CP-RTD proteins appeared to accumulate slightly earlier relative to WT (Fig 5C) suggesting an influence of ORF3a on translation of the other sgRNA1 ORFs. Conversely, increasing translation of ORF3a dramatically inhibited translation of the other sgRNA1-encoded proteins. These drastic effects were not seen *in vitro*, most likely due to the more efficient and less competitive translation conditions of the wheat germs extract, where ribosomes are not limiting.

### Role of P3a in virus movement

The dispensability of P3a for replication in protoplasts was expected, because previous deletion analysis of infectious clones showed that large deletions that included ORF3a and ORFs 3, 4, and 5 [61], or mutations that prevented sgRNA1 synthesis [32] did not significantly reduce replication of BYDV RNA in protoplasts. Similarly, none of the products of ORFs 3, 4 or 5 of TuYV are needed for RNA replication in protoplasts [46].

Agroinoculation of the two hosts tested, *A*. *thaliana* and *N*. *benthamiana*, triggered local infection by all TuYV-3a mutants. However systemic infection was very inefficient or nonexistent in these hosts. TuYV particle formation is a prerequisite for long distance trafficking [48]. However viral particles were easily observed in leaves inoculated with these mutants (TuYV-3aAGC and TuYV-3a2stop, Fig 8). This indicates that P3a is not required for the encapsidation process and that the lack of systemic movement is not due to packaging deficiency, thus reinforcing a direct role for P3a in viral systemic spread. The P3a-overexpressing mutant TuYV-3aAUG showed a drastically reduced expression of CP, CP-RTD and P4 that correlates with its incapacity to form virions as shown in Fig 8 and explains its deficiency in systemic movement.

Importantly, the P3a-defective mutant TuYV-3aAGC was capable of long distance trafficking when P3a was supplied *in trans* by either replicating virus (TuYV-3aAUG) or from a non-replicating plasmid (Fig 7). These results demonstrate unequivocally that the P3a protein is a key factor in long distance movement that functions *in trans*. This raises the issue of the mode of action of P3a. One hypothesis could be that P3a functions only in the cell where it is expressed and assists the virus in its exit from the infected phloem cells and loading into the phloem. This could be achieved by increasing the size exclusion limit of the specialized plasmodesmata that connect phloem companion cells and the sieve tube, the so-called pore plasmodesmal unit (PPU) [62]. Polerovirus particles have indeed been detected in these PD [15, 63]. In this case, virus accumulation in systemic leaves would result exclusively from replication at primary infection sites. A second hypothesis could be that P3a either facilitates its own movement far beyond the agroinfiltrated cells, and/or that the complemented virus (TuYV-3aAGC) brings the plasmid-expressed P3a protein along with it to the phloem to permit unloading from the sieve element into phloem cells of neighboring leaves.

Bioinformatic predictions highlighted a putative trans-membrane domain which seems in contradiction with this hypothesis, except if during the infection cycle P3a could associate with another factor (i.e. CP or CP-RTD) to move long distance in the phloem. The structural proteins CP and CP-RT* (the encapsidated truncated form of the CP-RTD), and also CP-RTD possibly in its free form, were detected in phloem exudates of CABYV-infected cucumber plants [64]. These proteins might interact with P3a and move in the phloem until they reach, with or without virions, new sites in upper leaves.

Wild-type TuYV generates only minute amounts of P3a from a non-AUG initiation codon (Figs 3, 4, and 5) suggesting that only low amounts of P3a are required. The P3a-defective AGC mutant should therefore not be limited in P3a supply when complemented with the AUG mutant which overproduces P3a (Figs 4 and 5). In contrast, the AUG mutant—defective in CP, CP-RTD and P4 production—requires much larger amounts of these proteins from the complementing AGC mutant. This may explain the skewed ratio TuYV-3aAGC/TuYV-3aAUG in favor of the AGC mutant, with a mean value of 2.4 in double-infected plants.

### Trafficking

Sub-cellular localization studies with fluorescent protein fusions in infiltrated *N*. *benthamiana* leaves showed that P3a is targeted to the Golgi apparatus, and also close to plasmodesmata (Fig 9). Both subcellular locations are in accordance with a role in viral movement. Thus, we speculate that the inability of P3a-GFP to significantly complement the P3a-deficient mutant TuYV-3aAGC was due to the GFP domain interfering with interactions of P3a with viral components (RNA or protein) necessary for movement, rather than being due to impaired subcellular localization. The specific targeting of P3a-GFP indicates a close association with the host endomembrane network likely through the transmembrane domain predicted in P3a. The ER and the Golgi apparatus constitute the core components of the secretory pathway, suggesting movement processes similar to thoses of other viruses. Small movement proteins of carmoviruses [65, 66] and potyviruses [67–69] also usurp the secretory pathway, and the TGB3 protein of potexviruses drives TGB2 protein-induced vesicles via the ER to form punctate caps on the cytoplasmic orifices of PD, similarly to the P3a protein [70].

Although we have shown function and localization only for TuYV P3a, it is highly likely that P3a of the other poleroviruses and luteoviruses has the same function, given (i) that P3a is required for TuYV movement in both *Arabidopsis* and *N*. *benthamiana*, (ii) the amino acid sequence conservation of P3a among diverse luteovirids (S1 Fig), and (iii) the functional conservation of all the neighboring ORFs on sgRNA1. It is noteworthy that, in addition to P3a, the P4 movement protein of PLRV also localizes to PD [16], facilitated by actin- and ER-Golgi-dependent transport [71]. Ectopically expressed TuYV P4 similarly targets PD but the trafficking pathway has not been studied yet (Julia De Cillia and V.Z-G. personal communication). Like conventional movement proteins MPs, P4 binds single-stranded RNA, dimerizes, is subject to phosphorylation, and increases the PD size exclusion limit [16, 18, 72, 73]. Remarkably P4 was found to be a host-specific movement protein. PLRV and TuYV P4-deficient mutants were reported to spread systemically in some, but not all, hosts [17, 74]. This raises questions such as whether P3a and P4 of TuYV act cooperatively on the same viral entity (virions or ribonucleoprotein complexes, RNP), or whether one protein promotes movement of RNP and the other virions. Another alternative mode of action of both P3a and P4 proteins could be that they function on specific PD of certain phloem cells, at certain development stages or even in specific hosts [17, 71]. Are P3a and P4 proteins specialized for a specific virus transport through “conventional” PD or through PPU? Since we have shown that in the absence of P3a TuYV long-distance transport is impaired, it seems more likely that P3a could play a role in the viral movement across PPUs.

In addition to P4 and P3a, CP and CP-RTD also participate in polerovirus and luteovirus movement. The CP is essential for TuYV long-distance movement through its ability to form particles [48]. CP-RTD occurs *in planta* in two forms, the full-length protein (the non-structural form) and CP-RT*, which is a C-terminally truncated form incorporated into virions [75, 76]. The N-terminal part of the CP-RTD is required for TuYV to move between nucleated vascular cells [15]. Both CP-RTD and CP-RT* were shown to be required for efficient long distance movement of CABYV [64]. The discovery that the P3a protein is involved in systemic trafficking adds more complexity to this process.

Interestingly, assigned (PEMV1) and putative (*Citrus vein enation virus*) members of the third *Luteoviridae* genus, *Enamovirus*, lack ORF3a. They also lack P4, and the carboxy-terminal half of the readthrough domain, both of which have been implicated in cell-to-cell and systemic movement [17, 75]. Instead, PEMV1 relies on a protein or proteins encoded by an associated umbravirus (PEMV2) for systemic movement in the plant beyond the phloem cells [19] which renders both viruses mechanically transmissible. Apparently, because PEMV has a movement mechanism different from the other *Luteoviridae*, it does not require P4, the C-terminus of RTD, or P3a, which suggests that P3a may act in concert with P4 and the C-terminus of RTD for virus movement. Further understanding of luteo/poleroviral movement will require us to decipher the precise function and interplay of these multiple viral proteins involved in movement.

## Materials and Methods

### Computational analyses

Sequences were processed and analyzed using EMBOSS [77], BLAST [78], CLUSTALW [79] and MLOGD [37]. Transmembrane regions were predicted with TMHMM v2.0 [80].

All *Luteoviridae* sequences available in GenBank as of 16 Nov 2013 were downloaded. Patent sequences were removed. The remaining nucleotide sequences were used to generate a BLAST database [78]. Sequences with coverage of the ORF3/3a region were identified by applying TBLASTN to the NC_004750 (BYDV) P3 amino acid sequence and retaining sequences with ≥75% coverage and ≥30% identity (parameters sufficient to retrieve all luteovirus and polerovirus sequences with ORF3 coverage, as well as enamovirus sequences which were subsequently excluded), and then retaining sequences with sufficient flanking sequence 5' of the ORF3 AUG in order to cover the ORF3a region. In total, 459 luteovirid ORF3a-region sequences were retrieved (see S1 Datafile).

Sequences with complete or near-complete genome coverage were initially selected by taking a length cut-off limit of 5000 nt, followed by semi-automated inspection of ORF lengths and alignments. Sequences which were defective due to obvious disruptions (e.g. premature termination codons or insertions/deletions that disrupted the reading frame) in ORFs 0, 1, 2, 3, 4 or 5 were removed. Sequences were easily clustered into poleroviruses, luteoviruses and enamoviruses based on genome organization and ORF lengths. BYDV sequences (serotypes PAV, MAV, GAV, PAS and KerII) were separated from other luteovirus clades using a P1 phylogenetic tree (CLUSTALW amino acid alignment; CLUSTALX tree).

The full-length BYDV nucleotide sequences were aligned initially with CLUSTALW. For the MLOGD analysis and stop codon plots, to produce meaningful results it is important that sequences are aligned in-frame within coding ORFs. To ensure this, the ORF1-ORF2 and ORF3-ORF5 coding blocks were extracted from the BYDV nucleotide alignment; ORFs 1 and 2 were fused in-frame through the artificial insertion of 'N' at the frameshift site; then each of the two regions was translated, re-aligned as amino acid sequences, back-translated to a nucleotide alignment, the previously inserted 'N's in the ORF1-ORF2 alignment were removed, and the re-aligned regions were reinserted into the full-genome alignment. For the pan-polerovirus alignment, nucleotide sequences were too divergent for an initial full-genome nucleotide alignment to provide a suitable scaffold. Thus, for the polerovirus alignment, untranslated regions were not included in the alignment, except for 178 nt of sequence 5' of ORF3 in order to encompass the ORF3a region. For each genome sequence, the ORF0-ORF1-ORF2 and ORF3a-ORF3-ORF5 regions were extracted and the ORFs in each region were fused in-frame through the artificial insertion of 'NN' before the start of ORF1, 'N' at the ORF1/ORF2 frameshift site, and 'NN' before the start of ORF3 (except for NC_006265 where ORFs 3a and 3 do not overlap). Then each of the two regions was translated, aligned as amino acid sequences, back-translated to a nucleotide alignment, and the previously inserted 'N's were removed.

For the MLOGD analysis and stop codon plots (Fig 1B and 1D), reading frames were defined by mapping sequences onto a specific reference sequence (NC_004750.1 for Fig 1B and NC_003743.1 for Fig 1D). This is important since reading frames are often not preserved in intergenic regions. This 'correction' is relevant to four of the 97 full-genome polerovirus sequences (three detailed in Fig 2 and discussed in text, plus HM439608) where the reading frame of ORF3a is disrupted, besides NC_006265 (*Carrot red leaf virus*) where the reading frame of ORF3a with respect to that of ORF3 differs from normal due to a 3-nt intergenic region between ORFs 3a and 3.

The BYDV analysis (Fig 1B) is based on GenBank accessions AF218798, AF235167, AJ810418, AY220739, AY610953, AY610954, AY855920, D11028, D11032, D85783, EF043235, EF521828, EF521829, EF521831, EF521832, EF521833, EF521834, EF521835, EF521836, EF521837, EF521838, EF521840, EF521841, EF521842, EF521843, EF521844, EF521845, EF521846, EF521847, EF521849, EF521850, EU332307, EU332308, EU332309, EU332310, EU332311, EU332312, EU332313, EU332314, EU332315, EU332316, EU332317, EU332318, EU332319, EU332320, EU332321, EU332322, EU332323, EU332324, EU332325, EU332326, EU332327, EU332328, EU332329, EU332330, EU332331, EU332332, EU332333, EU332334, EU332335, EU332336, EU402386, EU402387, EU402388, EU402389, EU402390, EU402391, HE985229, KC571999, KC572000, KF523378, KF523379, KF523380, KF523381, KF523382 and X07653. The polerovirus analysis (Fig 1D) is based on GenBank accessions AB594828, AF157029, AF235168, AF352024, AF352025, AF453388, AF453389, AF453390, AF453391, AF453392, AF453393, AF453394, AF473561, AJ249447, AM072750, AM072751, AM072752, AM072753, AM072754, AM072755, AM072756, AY138970, AY695933, AY956384, D00530, D10206, D13953, D13954, DQ132996, EF521827, EF521830, EF521839, EF521848, EF529624, EU000534, EU000535, EU313202, EU636990, EU636991, EU636992, EU717545, EU717546, FM865413, GQ221223, GQ221224, GU167940, GU190159, GU327735, GU570006, GU570007, GU570008, HM439608, HM804471, HM804472, HQ245316, HQ245317, HQ245318, HQ245319, HQ245320, HQ245321, HQ245322, HQ342888, HQ388348, HQ388349, HQ388350, HQ388351, HQ439023, HQ827780, JF507725, JF925152, JF925153, JF925154, JF925155, JF939812, JF939813, JF939814, JQ346189, JQ346190, JQ346191, JQ420901, JQ420902, JQ420903, JQ420904, JQ420905, JQ700305, JQ700306, JQ700307, JQ700308, JQ862472, JX855134, KC121026, KC921392, L25299, X13063, X74789, X76931, X83110 and Y07496.

### Construction of TuYV genomic and subgenomic RNA1 mutants

pUC19 expression vector containing sgRNA cDNA sequence was obtained by cloning the region corresponding to the sgRNA (3259–5641 bases) with the upstream primer containing an XbaI restriction site and T7-promoter sequence (in italics), GAGGTCTAGA*TAATACGACTCACTATAGGG*ACACCCGATACCAGGAGAG, and the downstream primer containing an NcoI restriction site, GAGGCCATGGAGTGCCCAACTCTCTTTGG. The mutations were introduced via the QuikChange site-directed mutagenesis procedure (Agilent Technologies) using mutagenic primers for PCR and subsequent DpnI treatment of PCR mixture. Oligonucleotides used for mutagenesis (mutations in bold and italics): for the 3aAUG mutant: CTTAAGCAAACCCAATTAAAGATACAA***T***GGATTACAAATTCCTAGCAGGCTTCGCC and the reverse complement, for the 3aAGC mutant: CTTAAGCAAACCCAATTAAAGATACAA***GC***GATTACAAATTCCTAGCAGGCTTCGCC and the reverse complement, for 3a2stop mutant: CAGGCTTCGCCGCAGGCTTCGTTT***A***AT***A***GATACCAATATCCGTGATCAGTATC and the reverse complement, for FLAG tag mutant: CCCAATTAAAGATACAACGGATTACAAA***GACGACGACGATAAG***TTCCTAGCAGGCTTCGCCGCAGGC and the reverse complement.

In order to obtain the same mutations in the agroinfection vector pBinBW_0_ [13, 81] containing full-length TuYV cDNA sequence, the SpeI/SalI fragment of pBinBW_0_ was replaced with the corresponding mutated sequences. All constructs were sequenced to confirm the presence of the mutations. pBin-derived constructs were introduced by electroporation into *Agrobacterium tumefaciens* strain GV3101 [82].

### *In vitro* transcription and translation of TuYV sgRNA

Capped TuYV sgRNA transcripts were obtained by *in vitro* transcription using the bacteriophage T7 RNA polymerase and BglII-linearized pUC19 vectors containing WT and mutant sgRNA sequences [83]. Transcripts were translated in wheat germ extracts (Promega) according to the manufacturer's instructions using 80 mM potassium acetate and 0.77 μg of corresponding transcript in 12.5 μl reactions containing the amino acid mix without methionine and 0.6 μCi [^35^S]-methionine. Reactions were performed for 90 minutes and terminated by addition of an equal volume of 2×SDS-PAGE buffer [84] and incubated at 95°C for 5 min. Samples were run on a Tricine-SDS gel [85] (6% and 16% acrylamide gels for stacking and resolving gels respectively) for 2.5 hours. The gel was washed 3 times for 1 hour and fixed for 16h in 20% ethanol/10% acetic acid solution and then successively washed for 30 min with solutions containing 15%/7,5%/5%, 10%/5%/10%, 5%/2,5%/15% ethanol/acetic acid/PEG550 and finally with 20% PEG 550 for 1 hour to prevent gel cracking (http://sciphu.com/2008/03/use-of-polyethylene-glycol-for-drying-polyacrylamide-gel). The gel was dried for 2 hours at 70°C and exposed either with an X-ray film or with a PhosphoImager screen.

The CP and P4 relative quantities were calculated as areas under the corresponding peaks and normalized to the WT CP or P4 intensities. The quantification was performed using ImageJ software according to the standard procedure of the peak surface measurement (e. g. http://openwetware.org/wiki/Protein_Quantification_Using_ImageJ).

### *In vitro* transcription and protoplast infection

Full-length TuYV RNA transcripts were obtained by *in vitro* transcription using the T7 RNA polymerase and SalI-linearized pBS vectors containing WT or mutant TuYV cDNA sequences [83]. Capped transcripts were then used to inoculate *Chenopodium quinoa* protoplasts by electroporation as described previously [83], using 5 μg transcripts for 250,000 protoplasts and a pulse of 180 V. Protoplasts were harvested 44 hours post-inoculation (p.i.), and total proteins or RNAs were extracted as described previously [46, 83].

### Agroinfiltration and agroinoculation of plants

*Agrobacterium tumefaciens* GV3101 [86] containing empty pBin19 vector, pBinBW_0_, derived mutant vectors or protein-expressing vectors were grown for 24 hours, pelleted and incubated in buffer containing 10 mM MES (pH 5,6), 10 mM MgCl_2_ and 0.15 mM acetosyringone for 2 hours. Agro-infiltration was performed at an OD_600_ of 0.5 (when mixed infiltrations, OD_600_ was 0.5 for each culture) to 5-week old *A*. *thaliana* plants (ecotype Col0) or to 6-week old *N*. *benthamiana* plants. New upper leaves were harvested 3 weeks pi for RNA or protein analysis (100 mg). For infiltrated leaves analysis the samples were collected at indicated time points.

### Detection of viral proteins

Protoplasts were disrupted by addition of hot 2×SDS-PAGE buffer with subsequent heating at 95°C for 5 min. Plant total proteins were extracted by grinding 100 mg *A*. *thaliana* leaves in 250 μl of hot 2×SDS-PAGE buffer [84]. Proteins were separated in 10% or 12% SDS-PAGE gels and transferred onto PVDF Immobilon-P membrane (Millipore). Membranes were then blocked in PBS-Tw 1% buffer (150 mM NaCl, 2.7 mM KCl, 10 mM Na_2_HPO_4_, 1.5 mM KH_2_PO_4_, 1% Tween-20) with 5% fat-free milk for 2 hours and incubated with specific primary antibodies raised against CP, RTD or P4 proteins [10, 46]. The protein/antibody complex was detected by chemiluminescence (Lumi-LightPLUS kit, Roche).

To immunodetect the 3a protein, protein samples were run on a 16% acrylamide Tricine-SDS gel [85] for 2.5 hours as described above and transferred onto Immobilon-P^SQ^ membrane (Millipore). Membranes were then blocked in PBS-Tw 0.1% buffer with 1% BSA and incubated with primary antibodies raised against the FLAG epitope (Sigma) or a peptide corresponding to the P3a C-terminal 15 amino acids. The protein/antibody complex was detected by chemiluminescence (Lumi-LightPLUS kit, Roche).

### Detection of viral RNAs

RNAs from protoplasts were extracted as described by Veidt et al. [83]. Samples from infiltrated or upper *A*. *thaliana* leaves were ground in liquid nitrogen and RNAs were extracted using TriReagent (Sigma-Aldrich) according to the manufacturer instructions. 7.5 μg of RNA extracted from leaves or from 100,000 protoplasts were denatured and fractionated on a 1% formaldehyde-agarose gel [83] and transferred to nitrocellulose (Amersham Hybond-NX, GE Healthcare). Prehybridization was performed at 60°C for 2 h in PerfectHyb Plus buffer (Sigma). The radioactive probe was generated using the Prime-a-Gene labeling system (Promega) and a PCR product corresponding to the 3’-terminal 600 bases of TuYV genome as template. After hybridization and washing, the membrane was exposed onto an X-ray film or a Phosphoimager screen.

2 μg of RNA isolated from upper non-inoculated leaves were used as a template for the reverse transcription reaction using the SuperScript III system (Life Technologies) and a reverse complement oligonucleotide to the last 19 bases of TuYV genomic RNA as a primer. PCR was performed using Qiagen Taq polymerase and the oligonucleotides corresponding to the first and the last 19 bases of TuYV sgRNA1. Purified fragments were thereafter sequenced.

Real-time PCR was performed on cDNA corresponding to 20 ng of total RNA extracted from upper leaves of *N*. *benthamiana* plants infiltrated with the various recombinant agrobacteria using a LightCycler 480 II instrument (Roche). The reactions were carried out using the SYBR Green I Master (Roche). In order to distinguish the viral mutants present in the upper leaves infected with the mixture of AUG and AGC mutants, or to verify the progeny in the singly infected plants, four sets of primers were designed: one set of common primers to detect any TuYV RNA (named co-Tu-LP (CCAGGAGAGTAAAGAAGAAGAAAG) and co-Tu-RP (AAGCCTGCTAGGAATTTGTAATC)) and three sets of primers able to recognize specifically the TuYV WT, AUG or AGC mutated sequence (see S6 Fig). The forward oligonucleotide (co-Tu-LP, S6 Fig) located 74 nucleotides upstream of the mutation site was common for all viral RNA and the reverse primers ended precisely at the mutation site so that the last 1 or 2 nucleotides were different in WT, AUG and AGC primers. The specificity of the primers was confirmed with plasmids used for T7-transcription of WT and mutated viral sgRNAs. The *N*. *benthamiana* GAPDH gene (JQ256517.1) was used as reference gene. The corresponding forward and reverse primers used are GTGCCAAGAAGGTTGTGATC and CAAGGCAGTTGGTAGTGCAA respectively. We then normalized the values with those obtained with the common TuYV primers and finally for each specific primer set by one of the RNA samples (extracted from plants #22 for TuYV-WT, #28 for TuYV-3aAUG and TuYV-3aAGC). Therefore the values presented in S6 Fig can only be considered as relative and not quantitative values.

### ISEM

Virus particles were purified from 2.5 g of *A*. *thaliana* agroinfiltrated leaves using the classical protocol adapted to small volumes [87]. Virions were visualized by ISEM as described by Hipper et al. [48] using a TuYV polyclonal antiserum to capture viral particles on the grids before observation by transmission electron microscopy.

### Confocal laser-scanning microscopy

ORF3a was mobilised into pB7FWG2 or pB7RWG2 vectors [88] to obtain GFP- or RFP- fusions, respectively. Transient expression was performed by agroinfiltration on six week-old *N*. *benthamiana* using a bacterial OD_600_ of 0.3. For co-expressions, a 1:1 mixture of the two Agrobacteria transformants was infiltrated. For mRNA stabilization Agrobacteria containing the silencing suppressor P19-encoding vector were used at the final OD_600_ of 0.1. Confocal observations were performed between 24 and 30 hpi with leaf discs mounted with water and vacuum infiltrated. Confocal microscopy images were obtained with a Zeiss LSM700 or LSM780 inverted confocal laser microscope using a 40×oil immersion objective. The excitation wavelength for GFP and RFP detection was 488 and 561 nm, respectively.

To visualize PD-localized callose, leaf disks were vacuum-infiltrated with aniline blue solution (0.1% aniline blue in 67 mM phosphate buffer pH 8). Leaf disks were incubated in dark at room temperature for 15 minutes before imaging using a Zeiss LSM700/780 laser scanning confocal microscope. The excitation wavelength for aniline blue was 405 nm.

## Supporting Information

S1 Datafile Annotated nucleotide sequences for ORF3a and flanking regions. This includes all luteovirus and polerovirus sequences available in GenBank as of 16 Nov 2013 with coverage of the ORF3a region.
(PDF)

S1 Fig Sequence analysis of P3a. Predicted P3a peptide sequences for representative luteovirus and polerovirus NCBI RefSeq sequences (GenBank accession numbers indicated at left). NC_004756 was translated under the assumption that the single-nucleotide deletion (pink '-' in Fig 2) is a sequencing error (see main text); hence the ambiguous amino acid code 'X' in the NC_004756 P3a sequence. A predicted transmembrane region, conserved in all sequences except the N-terminally truncated *Beet chlorosis virus* and *Beet mild yellowing virus* sequences (see text), is indicated above the alignment. Annotated initiation sites are based on the identity and context of potential initiation codons, and comparative sequence analysis. Note that multiple initiation sites may be utilized in some species (e.g. see Fig 2). For illustrative purposes, peptide sequences are shown with the genetic-code decoding of the predicted initiator codon; however, non-AUG initiation codons are expected to be normally decoded by initiator Met-tRNA resulting in an N-terminal methionine, rather than the indicated amino acid, for each sequence.
(TIF)

S2 Fig Mutations introduced into ORF3a. These include substitution mutants TuYV-3aAUG (AUG), TuYV-3aAGC (AGC), TuYV-3a2stop (2stop), and insertion mutant (TuYV-3aFLAG). The ORF3a main initiation codon is in green and underlined; alternative ones are in green. The ORF3a stop codon is depicted in red while the CP and P4 initiation codons are respectively in blue and purple.
(TIF)

S3 Fig Quantification of the relative CP (A) and P4 (B) levels synthesized by the various TuYV-3a mutants compared to TuYV WT in *in vitro* translation experiments. *In vitro* synthesized subgenomic transcripts were incubated for 30 minutes in wheat germ extracts and radioactive proteins were subsequently fractionated on a 12% PAGE and exposed with a PhosphorImager screen. The bands corresponding to the 20 (P4) and 22 kDa (CP) products were quantified using ImageJ software (http://openwetware.org/wiki/Protein_Quantification_Using_ImageJ). The experiment was repeated twice. WT expression of CP or P4 was arbitrarily fixed to unity in both experiments.
(TIF)

S4 Fig Detection of FLAG-P3a protein in infected *C*. *quinoa* protoplasts. Commercial (SIGMA) specific anti-FLAG antibodies were used. LC, loading control of proteins stained on the membrane by Ponceau red.
(TIF)

S5 Fig Comparison of CP-RTD levels in various loadings of *C*. *quinoa* protoplasts infected with TuYV-WT or TuYV-3aAUG. Proteins were detected using specific antibodies against the RTD.
(TIF)

S6 Fig Detection of TuYV wild-type and mutant viral RNA by qRT-PCR in systemic leaves of *N*. *benthamiana* plants. Specific primers were used to detect viral RNA in plants agroinfiltrated with TuYV, TuYV-3aAGC and TuYV-3aAUG, or co-infiltrated with TuYV-3aAGC and agrobacteria transiently expressing P3a, P3a-GFP or GFP. **A.** Schematic representation of the position of the common primers (coTu-LP and coTu-RP, drawn as blue arrows) and specific primers (spTu-WT/AUG/AGC-RP, drawn as red arrow) used to amplify respectively all viruses or specifically the corresponding WT or mutant virus. The sequences of the common primer coTu-RP and primers specific for the wild-type and each mutant virus are shown, with the bases complementary to the last two bases of the ACG initiation codon (or corresponding mutant sequence) underlined in red. **B, C and D.** Specific amplification by qRT-PCR of TuYV (WT) (**B**), TuYV-3aAUG (AUG) (**C**) and TuYV-3aAGC (AGC) (**D**) viral progeny from plants infected with the indicated viruses (below the graph): TuYV (WT), TuYV-3aAGC and TuYV-3aAUG (AGC+AUG), or TuYV-3aAGC co-infiltrated with agrobacterium expressing the P3a (AGC+P3a), P3a-GFP (AGC+P3a-GFP) or GFP (AGC+GFP). Proteins names are indicated in green. Samples are from those with the same number in Fig 7.
(TIF)

S7 Fig Detection of P3a-GFP and P3a proteins extracted from *N*. *benthamiana* leaves. Leaves were infiltrated with the viral mutant TuYV-3aAGC (AGC) or agrobacteria transiently expressing P3a-GFP (P3a-GFP), GFP (GFP), or P3a (P3a), or co-infiltrated with TuYV-3aAGC + P3a-GFP (AGC + P3a-GFP), TuYV-3aAGC + GFP (AGC + GFP) or TuYV-3aAGC + P3a (AGC + P3a). Proteins expressed transiently from agrobacteria are labeled in green. Three days post-infiltration, proteins were detected using specific antibodies against GFP (panel A) or against P3a (panel B). LC, loading control of proteins stained on the membrane by Ponceau red.
(TIF)

S8 Fig Transmission electron micrograph images of virions. Virions (indicated by arrowheads) were negatively stained after partial purification from *C*. *quinoa* protoplasts that had been transfected with the indicated mutant TuYV RNA 48 h previously. Scale bar = 50 nM
(TIF)

S9 Fig Detection of P3a-GFP and P3a-RFP in *N*. *benthamiana* agro-infiltrated leaves. The immunoblots were incubated with antibody specific to GFP or RFP. Loading controls of proteins stained on the membranes by Ponceau red are indicated.
(TIF)

S10 Fig Colocalization of callose and P3a. Callose was detected by aniline blue staining (**A**), in *N*. *benthamiana* leaves expressing 3a-RFP (**B**), and their fluorescent signals merged (**C**). Scale bar 10 μm.
(TIF)
