# Supplementary material for: Discovery of a Small Non-AUG-Initiated ORF in Poleroviruses and Luteoviruses That Is Required for Long-Distance Movement
Source: PLoS Pathog. 2015 May 6;11(5):e1004868. doi: 10.1371/journal.ppat.1004868 (PMC4422679; doi:10.1371/journal.ppat.1004868)
Supplement: S1 Datafile — This includes all luteovirus and polerovirus sequences available in GenBank as of 16 Nov 2013 with coverage of the ORF3a region. (PDF) [file ppat.1004868.s001.pdf]

NNN Predicted ORF3a initiation sites
AUG ORF3 initiation codon
AUG ORF4 initiation codon
UNN ORF3a stop codon
UNN Upstream ORF3a-frame stop codons
UNN Premature ORF3a stop codon
HM439608 ORF3a-defective sequence
-/N Deletion/insertion in ORF3a-defective sequence
NNN ORF3a-preserving insertion in some BYDV sequences

```
NC_006265 GUU GUU GCA AUC GUC GGA AGC AAA GCC AAC UAC AAG AAA GCC ACC AUA GAU UAU AAA UUU UUA GCA GGU ACC GCC ACA GGA UUC AUU GUA GCG AUA CCA AUC UGU GUG AUA GGC UUA UAC UAC GUC UAU CUU AAA AUA UCC UCU AAC AUA CAA UCA AUC GUA AAC GAA UAC GGC AGA GGG UAA -UU UAU GGC CAC GUA UGG ACU CAC CAA GAG AGC
NC_004750 AAU CAA UAC UGC UUU CCA UAC CGG AAA ACC ACU CAC AAA ACG AAU AUU AAU UAC CAA AUU UUA GCU GGG UUU GGA AUA GGG UUU AUA GUU AGU AUA CCC UGU ACA UUA GCU CUC GCG UAC UUU AUU UAC AAU AAA GUU UCA GAC ACC ACU AGA GAG GUG GUG AAU GAA UUC AGU AGG UCG UAG AGG ACC UAG ACG CGC AAA UCA AAA UGG CAC AAG
DQ631849 AAU CAA UAC UAC UUU CCA UAC CGG AAA ACC ACU CAC AAA ACG AAU AUU AAU UAC CAA AUC UUA GCU GGG UUU GGA AUA GGG UUU AUA GUU UCU AUU CCC UGU ACA UUA GCU CUC UCC UAC UUU AUC UAC AAU AAA GUU UCU GAC ACC ACU AGG GAG GUG GUG AAU GAA UUC AGU AGG CCG UAG AGG ACC UAG ACG CGC AAA UCA AAA UGG CAC AAG
EU332333 AAU CAA UAC UAC UUU CCA UAC CGG AAA ACC ACU CAC AAA ACG AAU ACU AAU UAC CAA AUC UUA GCU GGG UUU GGA AUA GGG UUU AUA GUU UCU AUU CCC UGU ACA UUA GCU CUC UCC UAC UUU AUC UAC AAU AAA GUU UCU GAC ACC ACU AGG GAG GUG GUG AAU GAA UUC AGU AGG CCG UAG AGG ACC UAG ACG CGC AAA UCA AAA UGG CAC AAG
KF523382 AAU CAA UAC UAC UUU CCA UAC CGG AAA ACC ACU CAC AAA ACG ACU AUU AAU UAC CAA AUC UUA GCU GGG UUU GGA AUA GGG UUU AUA GUU UCU AUU CCC UGU ACA UUA GCU CUC UCC UAC UUU AUC UAC AAU AAA GUU UCU GAC ACC ACU AGG GAG GUG GUG AAU GAA UUC AGU AGG CCG UAG AAA UAA CCG CAG GAG AAA UGG CCC AAG GAG AGC
EU402388 AAU CAA UAC UAC UUU CCA UAC CGG AAA ACC ACU CAC AAA ACG ACU AUU AAU UAC CAA AUC UUA GCU GGG UUU GGA AUA GGG UUU AUA GUU UCU AUU CCC UGU ACA UUA GCU CUC UCC UAC UUU AUC UAC AAU AAA GUU UCU GAC ACC ACU AGG GAG GUG GUG AAU GAA UUC AGU AGG CCG UAG AAA UAA CCG CAG GAG AAA UGG CCC AAG GAG AGC
EU402387 AAU CAA UAC UAC UUU CCA UAC CGG AAA ACC ACU CAC AAA ACG ACU AUU AAU UAC CAA AUC UUA GCU GGG UUU GGA AUA GGG UUU AUA GUU UCU AUU CCC UGU ACA UUA GCU CUC UCC UAC UUU AUC UAC AAU AAA GUU UCU GAC ACC ACU AGG GAG GUG GUG AAU GAA UUC AGU AGG CCG UAG AAA UAA CCG CAG GAG AAA UGG CCC AAG GAG AGC
AY610953 AAU CAA UAC UAC UUU CCA UAC CGG AAA ACC ACU CAC AAA ACG ACU AUU AAU UAC CAA AUC UUA GCU GGG UUU GGA AUA GGG UUU AUA GUU UCU AUU CCC UGU ACA UUA GCU CUC UCC UAC UUU AUC UAC AAU AAA GUU UCU GAC ACC ACU AGG GAG GUG GUG AAU GAA UUC AGU AGG CCG UAG AAA UAA CCG CAG GAG AAA UGG CCC AAG GAG AGC
KF523381 AAU CAA UAC UAC UUU CCA UAC CGG AAA ACC ACU CAC AAA ACG ACU AUU AAU UAC CAA AUC UUA GCU GGG UUU GGA AUA GGG UUU AUA GUU UCU AUU CCC UGU ACA UUA GCU CUC UCC UAC UUU AUC UAC AAU AAA GUU UCU GAC ACC ACU AGG GAG GUG GUG AAU GAA UUC AGU AGG CCG UAG AAA UAA CCG CAG GAG AAA UGG CCC AAG GAG AGC
KF523380 AAU CAA UAC UAC UUU CCA UAC CGG AAA ACC ACU CAC AAA ACG ACU AUU AAU UAC CAA AUC UUA GCU GGG UUU GGA AUA GGG UUU AUA GUU UCU AUU CCC UGU ACA UUA GCU CUC UCC UAC UUU AUC UAC AAU AAA GUU UCU GAC ACC ACU AGG GAG GUG GUG AAU GAA UUC AGU AGG CCG UAG AAA UAA CCG CAG GAG AAA UGG CCC AAG GAG AGC
EU402390 AAU CAA UAC UAC UUU CCA UAC CGG AAA ACC ACU CAC AAA ACG ACU AUU AAU UAC CAA AUC UUA GCU GGG UUU GGA AUA GGG UUU AUA GUU UCU AUU CCC UGU ACA UUA GCU CUC UCC UAC UUU AUC UAC AAU AAA GUU UCU GAC ACC ACU AGG GAG GUG GUG AAU GAA UUC AGU AGG CCG UAG AAA UAA CCG CAG GAG AAA UGG CCC AAG GAG AGC
KF523379 AAU CAA UAC UAC UUU CCA UAC CGG AAA ACC ACU CAC AAA ACG ACU AUU AAU UAC CAA AUC UUA GCU GGG UUU GGA AUA GGG UUU AUA GUU UCU AUU CCC UGU ACA UUA GCU CUC UCC UAC UUU AUC UAC AAU AAA GUU UCU GAC ACC ACU AGG GAG GUG GUG AAU GAA UUC AGU AGG CCG UAG AAA UAA CCG CAG GAG AAA UGG CCC AAG GAG AGC
KF523378 AAU CAA UAC UAC UUU CCA UAC CGG AAA ACC ACU CAC AAA ACG ACU AUU AAU UAC CAA AUC UUA GCU GGG UUU GGA AUA GGG UUU AUA GUU UCU AUU CCC UGU ACA UUA GCU CUC UCC UAC UUU AUC UAC AAU AAA GUU UCU GAC ACC ACU AGG GAG GUG GUG AAU GAA UUC AGU AGG CCG UAG AAA UAA CCG CAG GAG AAA UGG CCC AAG GAG AGC
EU402391 AAU CAA UAC UAC UUU CCA UAC CGG AAA ACC ACU CAC AAA ACG ACU AUU AAU UAC CAA AUC UUA GCU GGG UUU GGA AUA GGG UUU AUA GUU UCU AUU CCC UGU ACA UUA GCU CUC UCC UAC UUU AUC UAC AAU AAA GUU UCU GAC ACC ACU AGG GAG GUG GUG AAU GAA UUC AGU AGG CCG UAG AAA UAA CCG CAG GAG AAA UGG CCC AAG GAG AGC
NC_004666 AAU CAA UAC UAC UUU CCA UAC CGG AAA ACC ACU CAC AAA ACG ACU AUU AAU UAC CAA AUC UUA GCU GGG UUU GGA AUA GGG UUU AUA GUU UCU AUU CCC UGU ACA UUA GCU CUC UCC UAC UUU AUC UAC AAU AAA GUU UCU GAC ACC ACU AGG GAG GUG GUG AAU GAA UUC AGU AGG CCG UAG AAA UAA CCG CAG GAG AAA UGG CCC AAG GAG AGC
AY610954 AAU CAA UAC UAC UUU CCA UAC CGG AAA ACC ACU CAC AAA ACG ACU AUU AAU UAC CAA AUC UUA GCU GGG UUU GGA AUA GGG UUU AUA GUU UCU AUU CCC UGU ACA UUA GCU CUC UCC UAC UUU AUC UAC AAU AAA GUU UCU GAC ACC ACU AGG GAG GUG GUG AAU GAA UUC AGU AGG CCG UAG AAA UAA CCG CAG GAG AAA UGG CCC AAG GAG AGC
EU402389 AAU CAA UAC UAC UUU CCA UAC CGG AAA ACC ACU CAC AAA ACG ACU AUU AAU UAC CAA AUC UUA GCU GGG UUU GGA AUA GGG UUU AUA GUU UCU AUU CCC UGU ACA UUA GCU CUC UCC UAC UUU AUC UAC AAU AAA GUU UCU GAC ACC ACU AGG GAG GUG GGG AAU GAA UUC AGU AGG CCG UAG AAA UAA CCG CAG GAG AAA UGG CCC AAG GAG AGC
EU402386 AAU CAA UAC UAC UUU CCA UAC CGG AAA ACC ACU CAC AAA ACG ACU AUU AAU UAC CAA AUC UUA GCU GGG UUU GGA AUA GGG UUU AUA GUU UCU AUU CCC UGU ACA UUA GCU CUC UCC UAC UUU AUC UAC CAU AAA GUU UCU GAC ACC ACU AGG GAG GUG GUG AAU GAA UUC AGU AGG CCG UAG AAA UAA CCG CAG GAG AAA UGG CCC AAG GAG AGC
EF408157 AAU CAA UAC UGC UUU CCA UAC CGG AAA ACC ACU CAC AAA ACG AAU AUU AAU UAU CAA AUC UUA GCU GGG UUU GGA AUA GGG UUU AUA GUU AGU AUA CCC UGU ACA UUA GCU CUC ACG UAC UUU AUU UAC AAU AAA GUU UCA GAC ACC ACU AGA GAG GUG GUG AAU GAA UUC AGU AGG CCG UAG AGG ACC UAG ACG CGC AAA UCA AAA UGG CAC AAG
EF521828 AAU CAA UCC UCC UCU CCA UUC CGG AGA ACA ACU CGC AAA ACG AAU AUU AAU UAC CAA AUC UUA GCC GGG UUU GGG AUA GGG UUU AUA GUA AGU AUU CCC UGU ACA UUA GCU CUC ACG UAC UUU AUU UAC AAU AAA GUU UCA GAC ACC ACU AGA GAG GUG GUG AAU GAA UUC AGU AGG CCG UAG AGG ACC UAG ACG AGC AAA UCA AAA UGG CCC AAG
DQ115533 AAU CAA UAC UAC UUU CCA UAC CGG AAA ACC ACU CAC AAA ACG AAU AUU AAU UAC CAA AUU UUA GCU GGG UUU GGA AUA GGG UUU AUA GUG UCU AUA CCC UGU ACA UUA GCU CUC ACG UAC UUU AUU UAC AAC AAA GUU UCA GAC ACU ACU AGA GAG GUA GUG AAU GAA UUC AGU AGG CCG UAG AAA UAA CCG CAG GAG AAA UGG CCC AAG GAG AGC
EU332325 AAU CAA UCC UCC UCU CCA UUC CGG AGA ACA ACU CGC ACA ACG AAU AUU AAU UAC CAA AUC UUA GCC GGG UUU GGG AUA GGG UUU AUA GUA AGC AUA CCC UGU ACA UUA GCU CUC ACG UAC UUU AUU UAC AAU AAA GUU UCA GAC ACC ACU AGA GAG GUG GUG AAU GAA UUC AGU AGG CCG UAG AGG ACC UAG AAG AGC AAA CAA UGG CAC AAG AAG
EU332316 AAU CAA UCC UCC UCU CCA UUC CGG AGA ACA ACU CGC ACA ACG AAU AUU AAU UAC CAA AUC UUA GCC GGG UUU GGG AUA GGG UUU AUA GUA AGU AUU CCC UGU ACA UUA GCU CUC ACG UAC UUU AUU UAC AAU AAA GUU UCA GAC ACC ACU AGA GAG GUG GUG AAU GAA UUC AGU AGG CCG UAG AGG ACC UAG AAG AGC AAA CAA UGG CAC AAG AAG
DQ115528 AAU CAA UAC UAC UUU CCA UAC CGG AAA ACC ACU CAC AAA ACG AAU AUU AAU UAC CAA AUC UUA GCU GGG UUU GGA AUA GGG UUU AUA GUG UCU AUA CCC UGU ACA UUA GCU CUC ACG UAC UUU AUU UAC AAU AAA GUU UCA GAC ACU ACU AGA GAG GUA GUG AAU GAA UUC AGU AGG CCG UAG AAA UAA CCG CAG GAG AAA UGG CCC AAG GAG AGC
AJ810418 AAU CAA UAC UGC UUU CCA UAC CGG AAA ACC ACU CAC AAA ACG AAU AUU AAU UAC CAA AUC UUA GCU GGG UUU GGA AUA GGG UUU AUA GUA AGU AUA CCC UGU ACA UUA GCU CUC ACG UAC UUU AUU UAC AAU AAA GUU UCA GAC ACC ACU AGA GAG GUG GUG AAU GGA UUC AGU AGG CCG UAG AGG ACC UAG ACG AGC AAA UCA AAA UGG CCC AAG
EF408184 GAU CAA UAC UAC UUU CCA UAC CGG AAA ACC ACU CAC AUA ACG AAU AUU AAU UAU CAA AUC UUA GCU GGG UUU GGA AUA GGG UUU AUA GUU UCU AUA CCC UGU ACA UUA GCU CUC ACG UAC UUU AUU UAC AAU AAA GUU UCA GAC ACU ACU AGA GAG GUA GUG AAU GAG UUU AGU AGG CCG UAG AAA UAA CCG CAG GAG AAA UGG CCC AAG GAG AGC
NC_002160 AAU CAA UCC UCC UCU CCA UUC CGG AGA ACA ACU CGC AAA ACG AAU AUU AAU UAC CAA AUC UUA GCC GGG UUU GGG AUA GGG UUU AUA GUA AGU AUU CCC UGU ACA UUA GCU CUC ACG UAC UUU AUU UAC AAU AAA GUU UCA GAC ACC ACU AGA GAG GUG GUG AAU GAA UUC AGU AGG CCG UAG AGG ACC UAG ACG AGC AAA UCA AAA UGG CCC AAG
EF408182 AAU CAA UAC UAC UUU CCA UAC CGG AAA ACC ACU CAC AUA ACG AAU AUU AAU UAU CAA AUC UUA GCU GGG UUU GGA AUA GGG UUU AUA GUU UCU AUA CCC UGU ACA UUA GCU CUC ACG UAC UUU AUU UAC AAU AAA GUU UCA GAC ACU ACU AGA GAG GUA GUG AAU GAG UUU AGU AGG CCG UAG AAA UAA CCG CAG GAG AAA UGG CCC AAG GAG AGC
EF408181 AAU CAA UAC UAC UUU CCA UAC CGG AAA ACC ACU CAC AUA ACG AAU AUU AAU UAU CAA AUC UUA GCU GGG UUU GGA AUA GGG UUU AUA GUU UCU AUA CCC UGU ACA UUA GCU CUC ACG UAC UUU AUU UAC AAU AAA GUU UCA GAC ACU ACU AGA GAG GUA GUG AAU GAA UUU AGU AGG CCG UAG AAA UAA CCG CAG GAG AAA UGG CCC AAG GAG AGC
EF408180 AAU CAA UAC UAC UUU CCA UAC CGG AAA ACC ACU CAC AUA ACG AAU AUU AAU UAU CAA AUC UUA GCU GGG UUU GGA AUA GGG UUU AUA GUU UCU AUA CCC UGU ACA UUA GCU CUC ACG UAC UUU AUU UAC AAU AAA GUU UCA GAC ACU ACU AGA GAG GUA GUG AAU GAA UUU AGU AGG CCG UAG AAA UAA CCG CAG GAG AAA UGG CCC AAG GAG AGC
DQ631846 --- --- -AC UAC UUU CCA UAC CGG AAA ACC ACU CAC AAA ACG AAU AUU AAU UAC CAA AUU UUA GCU GGG UUU GGA AUA GGG UUU AUA GUG UCU AUA CCC UGU ACA UUA GCU CUC ACG UAC UUU AUU UAC AAU AAA GUU UCA GAC ACU ACU AGA GAG GUA GUG AAU GAA UUC AGU AGG CCG UAG AAA UAA CCG CAG GAG AAA UGG CCC AAG GAG AGC
DQ631845 -AU CAA UAC UAC UUU CCA UAC CGG AAA ACC ACU CAC AAA ACG AAU AUU AAU UAC CAA AUU UUA GCU GGG UUU GGA AUA GGG UUU AUA GUG UCU AUA CCC UGU ACA UUA GCU CUC ACG UAC UUU AUU UAC AAU AAA GUU UCA GAC ACU ACU AGA GAG GUA GUG AAU GAA UUC AGU AGG CCG UAG AAA UAA CCG CAG GAG AAA UGG CCC AAG GAG AGC
DQ631844 -AU CAA UAC UAC UUU CCA UAC CGG AAA ACC ACU CAC AAA ACG AAU AUU AAU UAC CAA AUU UUA GCU GGG UUU GGA AUA GGG UUU AUA GUG UCU AUA CCC UGU ACA UUA GCU CUC ACG UAC UUU AUU UAC AAC AAA GUU UCA GAC ACU ACU AGA GAG GUA GUG AAU GAA UUC AGU AGG CCG UAG AAA UAA CCG CAG GAG AAA UGG CCC AAG GAG AGC
DQ631843 AAU CAA UAC UAC UUU CCA UAC CGG AAA ACC ACU CAC AAA ACG AAU AUU AAU UAC CAA AUU UUA GCU GGG UUU GGA AUA GGG UUU AUA GUG UCU AUA CCC UGU ACA UUA GCU CUC ACG UAC UUU AUU UAC AAU AAA GUU UCA GAC ACU ACU AGA GAG GUA GUG AAU GAA UUC AGU AGG CCG UAG AAA UAA CCG CAG GAG AAA UGG CCC AAG GAG AGC
DQ631842 AAU CAA UAC UAC UUU CCA UAC CGG AAA ACC ACU CAC AAA ACG AAU AUU AAU UAC CAA AUC UUA GCU GGG UUU GGA AUA GGG UUU AUA GUG UCU AUA CCC UGU ACA UUA GCU CUC ACG UAC UUU AUU UAC AAU AAA GUU UCA GAC ACU ACU AGA GAG GUA GUG AAU GAA UUC AGU AGG CCG UAG AAA UAA CCG CAG GAG AAA UGG CCC AAG GAG AGC
EF408183 AAU CAA UAC UAC UUU CCA UAC CGG AAA ACC ACU CAC AUA ACG AAU UUU AAU UAU CAA AUC UUA GCU GGG UUU GGA AUA GGG UUU AUA GUU UCU AUA CCC UGU ACA UUA GCU CUC ACG UAC UUU AUU UAC AAU AAA GUU UCA GAC ACU ACU AGA GAG GUA GUG AAU GAG UUU AGU AGG CCG UAG AAA UAA CCG CAG GAG AAA UGG CCC AAG GAG AGC
EF521850 AAU CAA UCC UCC UCU CCA UUC CGG AGA ACA ACU CGC ACA ACG AAU AUU GAU UAC CAA AUC UUG GCA GGG UUU GGA AUA GGG UUC AUA GUA GGU AUC CCC UGU ACA UUA GCU CUC ACG UAU UUU AUU UAC AAU AAA GUU UCA GAC ACU ACU AGA GAG GUA GUG AAU GAA UUC AGU AGG CCG UAG AGG ACC UAG ACG AGC AAA UCA AAA UGG CCC AAG
DQ631855 AAU CAA UCC UCC UCU CCA UUC CGG AGA ACG ACU CGC ACA ACG AAU AUU GAU UAC CAA AUC UUG GCA GGA UUU GGA AUA GGG UUU AUA GUA GGU AUC CCC UGU ACA UUA GCU CUC ACG UAC UUU AUU UAC AAU AAA GUU UCA GAC ACU ACU AGA GAG GUA GUG AAU GAA UUC AGU AGG CCG UAG AGG ACC UAG ACG AGC AAA UCA AAA UGG CCC AAG
EF408153 AAU CAA UCC UCC UCU CCA UUC CGG AGA ACA ACU CGC ACA ACG AAU AUU GAU UAC CAA AUC UUG GCA GGA UUU GGA AUA GGG UUU AUA GUA GGU AUC CCC UGU ACA UUA GCU CUC ACG UAC UUU AUU UAC AAU AAA GUU UCA GAC ACU ACU AGA GAG GUA GUG AAU GAA UUC AGU AGG CCG UAG AGG ACC UAG ACG AGC AAA UCA AAA UGG CCC AAG
DQ792506 -AU CAA UCC UCC UCU CCA UUC CGG AGA ACA ACU CGC ACA ACG AAU AUU GAU UAC CAA AUC UUG GCA GGG UUU GGA AUA GGG UUU AUA GUA GGU AUC CCC UGU ACA UUA GCU CUC ACG UAU UUU AUU UAC AAU AAA GUU UCA GAC ACU ACU AGA GAG GUA GUG AAU GAA UUC AGU AGG CCG UAG AGG ACC UAG ACG AGC AAA UCA AAA UGG CCC AAG
EF408155 AAU CAA UCC UCC UCU CCA UUC CGG AGA ACA ACU CGC ACA ACG AAU AUU GAU UAC CAA AUC UUG GCA GGG UUU GGA AUA GGG UUU AUA GUA GGU AUC CCC UGU ACA UUA GCU CUC ACG UAC UUU AUU UAC AAU AAA GUU UCA GAC ACU ACU AGA GAG GUA GUG AAU GAA UUC AGU AGG CCG UAG AGG ACC UAG ACG AGC AAA UCA AAA UGG CCC AAG
EF408154 AAU CAA UCC UCC UCU CCA UUC CGG AGA ACA ACU CGC ACA ACG AAU AUU GAU UAC CAA AUC UUG GCG GGG UUU GGA AUA GGG UUU AUA GUA GGU AUC CCC UGU ACA UUA GCU CUC ACG UAU UUU AUU UAC AAU AAA GUU UCA GAC ACU ACU AGA GAG GUA GUG AAU GAA UUC AGU AGG CCG UAG AGG ACC UAG ACG AGC AAA UCA AAA UGG CCC AAG
EF408152 AAU CAA UCC UCC UCU CCA UUC CGG AGA ACA ACU CGC ACA ACG AAU AUU GAU UAC CAA AUC UUG GCA GGA UUU GGG AUA GGG UUU AUA GUA GGU AUC CCC UGU ACA UUA GCU CUC ACG UAC UUU AUU UAC AAU AAA GUU UCA GAC ACU ACU AGA GAG GUA GUG AAU GAA UUC AGU AGG CCG UAG AGG ACC UAG ACG AGC AAA UCA AAA UGG CCC AAG
AY855920 AAU CAA UCC UCC UCU CCA UUC CGG AGA ACA ACU CGC ACA ACG AAU AUU AAU UAC CAA AUC UUA GCC GGG UUU GGG AUA GGG UUU AUA GUA GGU AUA CCC UGU ACA UUA GCU CUC ACG UAC UUU AUU UAC AAU AAA GUU UCA GAC ACC ACU AGA GAG GUG GUG AAU GAA UUC AGU AGG CCG UAG GGG ACC UAG AAG AGC AAA CAA UGG CAC AAG AAG
HE985229 AAU CAA UAC UGC UUU CCA UAC CGG AAA ACC ACU CAC AAA ACG AAU AUU AAU UAC CAG AUU UUA GCU GGG UUU GGA AUA GGG UUU AUA GCU AGU AUA CCC UGU ACA UUA GCU CUC ACG UAC UUU AUU UAC AAU AAA GUU UCA GAC ACC ACU AGA GAG GUG GUC AAU GAA UUC AGU AGG CCG UAG GGG ACC UAG ACG AGC AAA UCA AAA UGG CCC AGG
NC_003680 AAU CAA UAC UAC UUU CCA UAC CGG AAA ACC ACU CAC AUA ACG AAU AUU AAU UAC CAG AUU UUA GCU GGG UUU GGA AUA GGG UUU AUA GCG UCU AUA CCC UGU ACA UUA GCU CUC ACG UAU UUU AUU UAC AAU AAA GUU UCA GAC ACU ACU AGA GAG GUA GUG AAU GAA UUC AGU AGG CCG UAG AAA CAA CCG CAG GAG GAA UGG CCC AAG GAG AGC
EU332314 AAU CAA UCC UCC UCU CCA UUC CGG AGA ACA ACU CGC ACA ACG AAU AUU AAU UAC CAA AUC UUA GCC GGG UUU GGG AUA GGG UUU AUU CUA AGU AUU CCC UGU ACA UUA GCU CUC ACG UAC UUU AUU UAC AAU AAA GUU UCA GAC ACC ACU AGA GAG GUG GUG AAU GAA UUC AGU AGG CCG UAG AGG ACC UAG AAG AGC AAA UAA UGG CAC ACG AAG
EU332317 AAU CAA UCC UCC UCU CCA UUC CGG AGA ACA ACU CGC ACA ACG AAU AUU AAU UAC CAA AUC UUA GCC GGG UUU GGG AUA GGG UUU AUC ACU AGU AUU CCC UGU ACA UUA GCU CUC ACG UAC UUU AUU UAC AAU AAA GUU UCA GAC ACC ACU AGG GAG GUG GUG AAU GAA UUC AGU AGG CCG UAG AGG ACC UAG AAG AGC AAA UAA UGG CAC ACG AAG
EU332336 AAU CAA UCC UCC UCU CCA UUC CGG AGA ACA ACU CGC ACA ACG AAU AUU AAU UAC CAA AUC UUA GCC GGG UUU GGG AUA GGG UUU AUC ACU AGU AUU CCC UGU ACA UUA GCU CUC ACG UAC UUU AUU UAU AAU AAA GUU UCA GAC ACC ACU AGA GAG GUG GUG AAU GAA UUC AGU AGG CCG UAG AGG ACC UAG AAG AGC AAA UAA UGG CAC ACG AAG
EU332331 AAU CAA UCC UCC UCU CCA UUC CGG AGA ACA ACU CGC ACA ACG AAU AUU AAU UAC CAA AUC UUA GCC GGG UUU GGG AUA GGG UUU AUC ACU AGU AUU CCC UGU ACA UUA GCU CUC ACG UAC UUU AUU UAC AAU AAA GUU UCA GAC ACC ACU AGA GAG GUG GUG AAU GAA UUC AGU AGG CCG UAG AGG ACC UAG AAG AGC AAA CAA UGG CAC ACG AAG
EU332313 AAU CAA UCC UCC UCU CCA UUC CGG AGA ACA ACU CGC ACA ACG AAU AUU AAU UAC CAA AUC UUA GCC GGG UUU GGG AUA GGG UUU AUU ACU AGU AUU CCC UGU ACA UUA GCU CUC ACG UAC UUU AUU UAC AAU AAA GUU UCA GAC ACC ACU AGA GAG GUG GUG AAU GAA UUC AGU AGG CCG UAG AGG ACC UAG AAG AGC AAA CAA UGG CAC AAG AAG
EU332321 AAU CAA UCC UCC UCU CCA UUC CGG AGA ACA ACU CGC ACA ACG AAU AUU AAU UAC CAA AUC UUA GCC GGG UUU GGG AUA GGG UUU AUU ACU AGU AUU CCC UGU ACA UUA GCU CUC ACG UAC UUU AUU UAC AAU AAA GUU UCA GAC ACC ACU AGA GAG GUG GUG AAU GAA UUC AGU AGG CCG UAG AGG ACC UAG AAG AGC AAA CAA UGG CAC ACG AAG
EU332320 AAU CAA UCC UCC UCU CCA UUC CGG AGA ACA ACU CGC ACA ACG AAU AUU AAU UAC CAA AUC UUA GCC GGG UUU GGG AUA GGG UUU AUU ACU AGU AUU CCC UGU ACA UUA GCU CUC ACG UAC UUU AUU UAC AAU AAA GUU UCA GAC ACC ACU AGA GAG GUG GUG AAU GAA UUC AGU AGG CCG UAG AGG ACC UAG AAG AGC AAA CAA UGG CAC ACG AAG
EU332324 AAU CAA UCC UCC UCU CCA UUC CGG AGA ACA ACU CGC ACA ACG AAU AUU AAU UAC CAA AUC UUA GCC GGG UUU GGG AUA GGG UUU AUU ACU AGU AUU CCC UGU ACA UUA GCU CUC ACG UAC UUU AUU UAC AAU AAA GUU UCA GAC ACC ACU AGA GAG GUG GUG AAU GAA UUC AGU AGG CCG UAG AGG ACC UAG AAG AGC AAA UAA UGG CAC ACG AAG
EU332323 AAU CAA UCC UCC UCU CCA UUC CGG AGA ACA ACU CGC ACA ACG AAU AUU AAU UAC CAA AUC UUA GCC GGG UUU GGG AUA GGG UUU AUU ACU AGU AUU CCC UGU ACA UUA GCU CUC ACG UAC UUU AUU UAC AAU AAA GUU UCA GAC ACC ACU AGA GAG GUG GUG AAU GAA UUC AGU AGG CCG UAG AGG ACC UAG AAG AGC AAA CAA UGG CAC ACG AAG
EU332319 AAU CAA UCC UCC UCU CCA UUC CGG AGA ACA ACU CGC ACA ACG AAU AUU AAU UAC CAA AUC UUA GCC GGG UUU GGG AUA GGG UUU AUU ACU AGU AUU CCC UGU ACA UUA GCU CUC ACG UAC UUU AUU UAC AAU AAA GUU UCA GAC ACC ACU AGA GAG GUG GUG AAU GAA UUC AGU AGG CCG UAG AGG ACC UAG AAG AGC AAA CAA UGG CAC AAG AAG
EU332318 AAU CAA UCC UCC UCU CCA UUC CGG AGA ACA ACU CGC AUA ACG AAU AUU AAU UAC CAA AUC UUA GCC GGG UUU GGG AUA GGG UUU AUU ACU AGU AUU CCC UGU ACA UUA GCU CUC ACG UAC UUU AUU UAC AAU AAA GUU UCA GAC ACC ACU AGA GAG GUG GUG AAU GAA UUC AGU AGG CCG UAG AGG ACC UAG AAG AGC AAA CAA UGG CAC AAG AAG
EU332310 AAU CAA UCC UCC UCU CCA UUC CGG AGA ACA ACU CGC ACA ACG AAU AUU AAU UAC CAA AUC UUA GCC GGG UUU GGG AUA GGG UUU AUU ACU AGU AUU CCC UGU ACA UUA GCU CUC ACG UAC UUU AUU UAC AAU AAA GUU UCA GAC ACC ACU AGA GAG GUG GUG AAU GAA UUC AGU AGG CCG UAG AGG ACC UAG AAG AGC AAA CAA UGG CAC AAG AAG
EU332308 AAU CAA UCC UCC UCU CCA UUC CGG AGA ACA GCU CGC ACA ACG AAU AUU AAU UAC CAA AUC UUA GCC GGG UUU GGG AUA GGG UUU AUU ACU AGU AUU CCC UGU ACA CUA GCU CUC ACG UAC UUU AUU UAC AAU AAA GUU UCA GAC ACC ACU AGA GAG GUG GUG AAU GAA UUC AGU AGG CCG UAG AGG ACC UAG AAG AGC AAA CAA UGG CAC ACG AAG
DQ631841 AAU CAA UAC UAC UUU CCA UAC CGG AAA ACC ACU CAC AAA ACG AAU AUU AAU UAC CAA AUC UUA GCU GGG UUU GGA AUA GGG UUU AUA GUG UCU AUA CCC UGU ACA UUA GCU CUC ACG UAC UUU GUU UAC AAU AAA GUU UCA GAC ACU ACU AGA GAG GUA GUG AAU GAA UUC AGU AGG CCG UAG AAA UAA CCG CAG GAG AAA UGG CCC AAG GAG AGC
DQ631840 AAU CAA UAC UAC UUU CCA UAC CGG AAA ACC ACU CAC AAA ACG AAU AUU AAU UAC CAA AUC UUA GCU GGG UUU GGA AUA GGA UUU AUA GUG UCU AUA CCC UGU ACA UUA GCU CUC ACG UAC UUU GUU UAC AAU AAA GUU UCA GAC ACU ACU AGA GAG GUA GUG AAU GAA UUC AGU AGG CCG UAG AAA UAA CCG CAG GAG AAA UGG CCC AAG GAG AGC
EU332327 AAU CAA UCC UCC UCU CCA UUC CGG AGA ACA ACU CGC ACA ACG AAU AUU AAU UAC CAA AUC UUA GCC GGA UUU GGG AUA GGG UUU AUC GUA AGU AUU CCC UGU ACA UUA GCU CUC ACG UAC UUU GUU UAC AAU AAA GUU UCA GAC ACC ACU AGA GAG GUG GUG AAU GAA UUC AGU AGG CCG UAG AGG ACC UAG AAG AGC AAA CAA UGG CAC AAG AAG
EU332326 AAU CAA UCC UCC UCU CCA UUC CGG AGA ACA ACU CGC ACA ACG AAU AUU AAU UAC CAA AUC UUA GCC GGA UUU GGG AUA GGG UUU AUC GUA AGU AUU CCC UGU ACA UUA GCU CUC ACG UAC UUU GUU UAC AAU AAA GUU UCA GAC ACC ACU AGA GAG GUG GUG AAU GAA UUC AGU AGG CCG UAG AGG ACC UAG AAG AGC AAA CAA UGG CAC AAG AAG
EU332315 AAU CAA UCC UCC UCU CCA UUC CGG AGA ACA ACU CGC ACA ACG AAU AUU AAU UAC CAG AUC UUA GCC GGA UUU GGG AUA GGG UUU AUA GUA AGU AUU CCC UGU ACA UUA GCU CUC ACG UAC UUU GUU UAC AAU AAA GUU UCA GAC ACC ACU AGA GAG GUG GUG AAU GAA UUC AGU AGG CCG UAG AGG ACC UAG AAG AGC AAA CAA UGG CAC AAG AAG
EU332311 AAU CAA UCC UCC UCU CCA UAC CGG AGA ACA ACU CGC ACA ACG AAU AUU AAU UAC CAA AUC UUA GCC GGG UUC GGA AUA GGG UUC AUA GUA AGU AUU CCC UGU ACA UUA GCU CUC ACG UAC UUU GUU UAC AAU AAA GUU UCA GAC ACC ACU AGA GAG GUG GUG AAU GAA UUC AGU AGG CCG UAG AGG ACC UAG AAG AGC AAA CAA UGG CAC AAG AAG
DQ680136 AAU CAA UAC UCC UGG CAA UAC CAC AAA CAU CCC CGC AAU AUA GCU AUU AAU UAC CAA AUC UUG GCC GGG UUU GGC AUA GGG UUU AUU AUA UCU AUC CCC UGU ACA UUA GCU CUC ACG UAC UUU GUU UAC AAU AAA GUU UCA GAC ACC ACU AGA GAG GUG GUG AAU GAA UUC AGU AGG CCU UAA UUC CAG AAA GAG GAA GAG AAA UGG ACA ACC AAA
DQ680126 AAU CAA UAC UCC UUG CAA UAC CAC AAA CAU CCC CGC AAU AUA GCU AUU AAU UAC CAA AUC UUA GCC GGA UUU GGC AUA GGG UUU AUU AUA UCU AUU CCC UGU ACA UUA GCU CUC ACG UAC UUU GUG UAC AAU AAA GUU UCA GAC ACC ACU AGA GAG GUG GUG AAU GAA UUC AGU AGG CCU UAA UUC CAG AAA GAG GAA GAG AAA UGG ACA ACC AAA
DQ680128 --- --- --- --- --- --- --- --A AAA CAU CCC CGC AAU AUA GCU AUU AAU UAC CAA AUC UUG GCC GGA UUU GGU AUA GGG UUU AUU AUA UCU AUU CCC UGU ACA UUA GCU CUC ACG UAC UUU GUG UAC AAU AAA GUU UCA GAC ACC ACU AGA GAG GUG GUG AAU GAA UUC AGU AGG CCU UAA UUC CAG AAA GAG GAA GAG AAA UGG ACA ACC AAA
DQ680125 AAU CAA UAC UCC UGG CAA UAC CAC AAA CAU CCC CGC AAU AUA GCU AUU AAU UAC CAA AUC UUG GCC GGG UUU GGC AUA GGG UUU AUU AUA UCU AUC CCC UGU ACA UUA GCU CUC ACG UAC UUU GUG UAC AAU AAA GUU UCA GAC ACC ACU AGA GAG GUG GUG AAU GAA UUC AGU AGG CCU UAA UUC CAG AAA GAG GAA GAG AAA UGG ACA ACC AAA
DQ680117 AAU CAA UAC UCC UGG CAA UAC CAC AAA CAU CCC CGC AAU AUA GCU AUU AAU UAC CAA AUC UUG GCC GGG UUU GGC AUA GGG UUU AUU AUA UCU AUC CCC UGU ACA UUA GCU CUC ACG UAC UUU GUU UAC AAU AAA GUU UCA GAC ACC ACU AGA GAG GUG GUG AAU GAA UUC AGU AGG CCU UAA UUC CAG AAA GAG GAA GAG AAA UGG ACA ACC AAA
DQ680129 AAU CAA UAC UCC UGG CAA UAC CAC AAA CAU CCC CGC AAU AUA GCU AUU AAU UAC CAA AUC UUG GCC GGA UUU GGC AUA GGG UUU AUA AUA UCU AUU CCC UGU ACA UUA GCU CUC ACG UAC UUU GUU UAC AAU AAA GUU UCA GAC ACC ACU AGA GAG GUG GUG AAU GAA UUC AGU AGG CCU UAA UUC CAG AAA GAG GAA GAG AAA UGG ACA ACC AAA
DQ680122 AAU CAA UAC UCC UUG CAA UAC CAC AAA CAU CCC CGC AAU AUA GCU AUU AAU UAC CAA AUC UUA GCC GGA UUU GGU AUA GGG UUU AUU AUA UCU AUU CCC UGU ACA UUA GCU CUC ACG UAC UUU GUG UAC AAU AAA GUU UCA GAC ACC ACU AGA GAG GUG GUG AAU GAA UUC AGU AGG CCU UAA UUC CAG AAA GAG GAA GAG AAA UGG ACA AUC AAA
DQ680121 AAU CAA UAC UCC UGG CAA UAC CAC AAA CAU CCC UGC AAU AUA GCU AUU AAU UAC CAA AUC UUG GCC GGA UUU GGC AUA GGG UUU AUA AUA UCU AUU CCC UGU ACA UUA GCU CUC ACG UAC UUU GUU UAC AAU AAA GUU UCA GAC ACC ACU AGA GAG GUG GUG AAU GAA UUC AGU AGG CCU UAA UUC CAG AAA GAG GAA GAG AAA UGG ACA AUC AAA
DQ680114 AAU CAA UAC UCC UGG CAA UAC CAC AAA CAU CCC CGC AAU AUA GCU AUU AAU UAC CAA AUC UUG GCC GGA UUU GGC AUA GGG UUU AUA AUA UCU AUU CCC UGU ACA UUA GCU CUC ACG UAC UUU GUU UAC AAU AAA GUU UCA GAC ACC ACU AGA GAG GUG GUG AAU GAA UUC AGU AGG CCU UAA UUC CAG AAA GAG GAA GAG AAA UGG ACA AUC AAA
DQ680123 AAU CAA UAC UCC UGG CAA UAC CAC AAA CAU CCC CGC AAU AUA GCU AUU AAU UAC CAA AUC UUG GCC GGG UUU GGC AUA GGG UUU AUU AUA UCU AUC CCC UGU ACA UUA GCU CUC ACG UAC UUU GUU UAC AAU AAA GUU UCA GAC ACC ACU AGA GAG GUG GUG AAU GAA UUC AGU AGG CCU UAA UUC CAG AAA GAG GAA GAG AAA UGG ACA ACC AAA
DQ680115 -AU CAA UAC UCC UGG CAA UAC CAC AAA CAU CCC CGC AGU AUA GCU AUU AAU UAC CAA AUC UUG GCC GGG UUU GGC AUA GGG UUU AUU AUA UCU AUU CCC UGU ACA UUA GCU CUC ACG UAC UUU GUG UAC AAU AAA GUU UCA GAC ACC ACU AGA GAG GUG GUG AAU GAA UUC AGU AGG CCU UAA UUC CAG AAA GAG GAA GAG AAA UGG ACA ACC AAA
DQ680119 AAU CAA UAC UCC UGG CAA UAC CAC AAA CAU CCC CGC AAU AUA GCU AUU AAU UAC CAA AUC UUG GCC GGG UUU GGC AUA GGG UUU AUU AUA UCU AUC CCC UGU ACA UUA GCU CUC ACG UAC UUU GUU UAC AAU AAA GUU UCA GAC ACC ACU AGA GAG GUG GUG AAU GAA UUC AGU AGG CCU UAA UUC CAG AAA GAG GAA GAG AAA UGG ACA ACC AAA
DQ680116 AAU CAA UAC UCC UGG CAA UAC CAC AAA CAU CCC CGC AAU AUA GCU AUU AAU UAC CAA AUC UUG GCC GGG UUU GGC AUA GGG UUU AUU AUA UCU AUC CCC UGU ACA UUA GCU CUC ACG UAC UUU GUU UAC AAU AAA GUU UCA GAC ACC ACU AGA GAG GUG GUG AAU GAA UUC AGU AGG CCU UAA UUC CAG AAA GAG GAA GAG AAA UGG ACA ACC AAA
DQ680130 AAU CUA UAC UCC UUG CAA UAC CGC AAA CAU CCC CGC AAU AUA GCU AUU AAU UAC CAA AUC UUG GCC GGA UUU GGC AUA GGG UUU AUU AUA UCU AUU CCC UGU ACA UUA GCU CUC ACG UAC UUU GUG UAC AAU AAA GUU UCA GAC ACC ACU AGA GAG GUG GUG AAU GAA UUC AGU AGG CCU UAA UUC CAG AAA GAG GAA GAG AAA UGG ACA ACC AAA
DQ680135 AAU CAA UAC UCC UGG CAA UAC CAC AAA CAU CCC CGC AAU AUA GCU AUU AAU UAC CAA AUC UUG GCC GGG UUU GGC AUA GGG UUU AUU AUA UCU AUC CCC UGU ACA UUA GCU CUC ACG UAC UUU GUU UAC AAU AAA GUU UCA GAC ACC ACU AGA GAG GUG GUG AAU GAA UUC AGU AGG CCU UAA UUC CAG AAA GAG GAA GAG AAA UGG ACA ACC AAA
DQ680134 AAU CAA UAC UCC UGG CAA UAC CAC AAA CAU CCC CGC AAU AUA GCU AUU AAU UAC CAA AUC UUG GCC GGG UUU GGU AUA GGG UUU AUU AUA UCU AUU CCC UGU ACA UUA GCU CUC ACG UAC UUU GUU UAC AAU AAA GUU UCA GAC ACC ACU AGA GAG GUG GUG AAU GAA UUC AGU AGG CCU UAA UUC CAG AAA GAG GAA GAG AAA UGG ACA ACC AAA
DQ680133 AAU CAA UAC UCC UGG CAA UAC CAC AAA CAU CCC CGC AAU AUA GCU AUU AAU UAC CAA AUC UUG GCC GGA UUU GGC AUA GGG UUU AUU AUA UCU AUU CCC UGU ACA UUA GCU CUC ACG UAC UUU GUU UAC AAU AAA GUU UCA GAC ACC ACU AGA GAG GUG GUG AAU GAA UUC AGU AGG CCU UAA UUC CAG AAA GAG GAA GAG AAA UGG ACA ACC AAA
DQ680132 AAU CAA UAC UCC UGG CAA UAC CGC AAU CAU CCC CGC AAU AUA GCU AUU AAU UAC CAA AUC UUG GCC GGA UUU GGC AUA GGG UUU AUU AUA UCU AUU CCC UGU ACA UUA GCU CUC ACG UAC UUU GUU UAC AAU AAA GUU UCA GAC ACC ACU AGA GAG GUG GUG AAU GAA UUC AGU AGG CCU UAA UUC CAG AAA GAG GAA GAG AAA UGG ACA ACC AAA
DQ680131 AAU CAA UAC UCC UGG CAA UAC CGC AAU CAU CCC CGC AAU AUA GCU AUU AAU UAC CAA AUC UUG GCC GGA UUU GGC AUA GGG UUU AUU AUA UCU AUU CCC UGU ACA UUA GCU CUC ACG UAC UUU GUU UAC AAU AAA GUU UCA GAC ACC ACU AGA GAG GUG GUG AAU GAA UUC AGU AGG CCU UAA UUC CAG AAA GAG GAA GAG AAA UGG ACA ACC AAA
DQ680120 AAU CUA UAC UCC UUG CAA UAC CGC AAA CAU CCC CGC AAU AUA GCU AUU AAU UAC CAA AUC UUG GCC GGA UUU GGC AUA GGG UUU AUU AUA UCU AUU CCC UGU ACA UUA GCU CUC ACG UAC UUU GUG UAC AAU AAA GUU UCA GAC ACC ACU AGA GAG GUG GUG AAU GAA UUC AGU AGG CCU UAA UUC CAG AAA GAG GAA GAG AAA UGG ACA ACC AAA
X74789 UUG CAA GCA UUG GAA GUU CAA GUC UCG UUA CAU CAA CCG GAC AAA AUA GAU UAU AAA UUU UUA GCG GGA UUU GCU UUA GGA UUC UCA UCC GCA AUC CCA UUU UCA GUA GCC GGU UUA UAU UUU GUU UAC CUA AAG AUU UCC UCC CAC GUG CGA UCA AUU GUU AAU GAG UAC GGU CGU GGU UAA AGG AAA UGU CAA UGG UGG UGU ACA ACA ACC AAG
JQ346190 UUG CAA GCA UUG GAA GUU CAA GCC UCG UUA CAU UAA CCG GAC AAA AUA GAU UAU AAA UUC UUA GCG GGA UUU GCU UUA GGA UUC UCA UCC GCA AUC CCA UUU UCA GUA GCC GGU UUA UAU UUU GUU UAC CUA AAG AUU UCC UCC CAC GUG CGA UCA AUU GUU AAU GAG UAC GGU CGU GGU UAA AGG AAA UGU CAA UGG UGG UGU ACA ACA ACC AAG
JQ420903 UUG CAG GCA UUG GAA GUU CAA GCC UCG UUA CAU CAA CCG GAC AAA AUA GAU UAU AAA UUC UUA GCG GGA UUU GCU UUA GGA UUC UCA UCC GCA AUC CCA UUU UCA GUA GCC GGU UUA UAU UUU GUU UAC CUA AAG AUU UCC UCC CAC GUG CGA UCA AUU GUU AAU GAG UAC GGU CGU GGU UAG AGG AAA UGU CAA UGG UGG UGU ACA ACA ACC AAG
EU717545 UUG CAA GCA UUG GAA GUU CAA GCC UCG UUA CAU CAA CCG GAC AAA AUA GAU UAU AAA UUC UUA GCG GGA UUU GCU UUA GGA UUC UCA UCC GCA AUC CCA UUU UCA GUA GCC GGU UUA UAU UUU GUU UAC CUA AAG AUU UCC UCC CAC GUG CGA UCA AUU GUU AAU GAG UAC GGU CGU GGU UAA AGG AAA UGU CAA UGG UGG UGU ACA ACA ACC AAG
AY138970 UUG CAA GCA UUG GAA GUU CAA GUC UCG UUA CAU CAA CCG GAC AAA AUA GAU UAU AAA UUU UUA GCG GGA UUU GCU UUA GGA UUC UCA UCC GCA AUC CCA UUU UCA GUA GCC GGU UUA UAU UUU GUU UAC CUA AAG AUU UCC UCC CAC GUG CGA UCA AUU GUU AAU GAG UAC GGU CGU GGU UAA AGG AAA UGU CAA UGG UGG UGU ACA ACA ACC AAG
AF453393 UUG CAA GCA UUG GAA GUU CAA GCC UCG UUA CAU CAA CCG GAC AAA AUA GAU UAU AAA UUC UUA GCG GGA UUU GCU UUA GGA UUC UCA UCC GCA AUC CCA UUU UCA GUA GCC GGU UUA UAU UUC GUU UAC CUA AAG AUU UCC UCC CAC GUG CGA UCA AUC GUU AAU GAG UAC GGU CGU GGU UAA AGG AAA UGU CAA UGG UGG UGU ACA ACA ACC AAG
JQ420905 UUG CAA GCA UUG GAA GUU CAA GCC UCG UUA CAU CAA CCG GAC AAA AUA GAU UAU AAA UUC UUA GCG GGA UUU GCU UUA GGA UUC UCA UCC GCA AUC CCA UUU UCA GUA GCC GGU UUA UAU UUU GUU UAC CUA AAG AUU UCC UCC CAC GUG CGA UCA AUC GUU AAU GAG UAC GGU CGU GGU UAA AGG AAA UGU CAA UGG UGG UAU ACA ACA ACC AAG
EU313202 UUG CAA GCA UUG GAA GUU CAA GCC UCG UUA CAU CAA CCG GAC AAA AUA GAU UAU AAA UUC UUA GCG GGA UUU GCU UUG GGA UUC UCA UCC GCA AUC CCA UUU UCA GUA GCC GGU UUA UAU UUU GUU UAC CUA AAG AUU UCC UCC CAC GUG CGA UCA AUA GUU AAU GAG UAC GGU CGU GGU UAA AGG AAA UGU CAA UGG UGG UGU ACA ACA ACC AAG
JX855134 UUG CAA GCA UUG GAA GUU CAA GCC UCG UUA CAU CAA CCG GAC AAA AUA GAU UAU AAA UUC UUA GCG GGA UUC GCU UUA GGA UUU UCA UCC GCA AUC CCA UUU UCA GUA GCC GGU UUA UAU UUU GUU UAC CUA AAG AUU UCC UCC CAC GUG CGA UCA AUU GUU AAU GAG UAC GGU CGU GGU UAA AGG AAA UGU CAA UGG UGG UGU UCA ACA ACC AAG
JQ420901 UUG CAA GCA UUG GAA GUU CAA GCC UCG UUA CAU CAA CCG GAC AAA AUA GAU UAU AAA UUC UUA GCG GGA UUU GCU UUA GGA UUU UCA UCC GCA AUC CCA UUU UCA GUA GCC GGU UUA UAU UUU GUU UAC CUA AAG AUU UCC UCC CAC GUG CGA UCA AUU GUU AAU GAG UAC GGU CGU GGU UAA AGG AAA UGU CAA UGG UGG UGU UCA ACA ACC AAG
D13954 UUG CAA GCA UUG GAA GUU CAA GCC UCG UUA CAU CAA CCG GAC AAA AUA GAU UAU AAA UUC UUA GCG GGA UUC GCU UUA GGA UUU UCA UCC GCA AUC CCA UUU UCA GUA GCC GGU UUA UAU UUU GUU UAC CUA AAG AUU UCC UCC CAC GUG CGA UCA AUU GUU AAU GAG UAC GGU CGU GGU UAA AGG AAA UGU CAA UGG UGG UGU ACA ACA ACC AAG
JQ346191 UUG CAA GCA UUG GAA GUU CAA GCC UCG UUA CAU CAA CCG GAC AAA AUA GAU UAU AAA UUC UUA GCG GGA UUU GCU UUA GGA UUC UCA UCC GCA AUC CCA UUU UCA GUA GCC GGU UUA UAU UUU GUU UAC CUA AAG AUU UCC UCC CAC GUG CGA UCA AUU GUU AAU GAG UAC GGU CGU GGU UAA AGG AAA UGU CAA UGG UGG UGU ACA ACA ACC AAG
NC_001747 UUG CAA GCA UUG GAA GUU CAA GCC UCG UUA CAU CAA CCG GAC UAA AUA GAU UAU AAA UUC UUA GCG GGA UUU GCU UUA GGA UUC UCA UCC GCA AUC CCA UUU UCA GUA GCC GGU UUA UAU UUU GUU UAC CUA AAG AUU UCC UCC CAC GUG CGA UCA AUU GUU AAU GAG UAC GGU CGU GGU UAA AGG AAA UGU CAA UGG UGG UGU ACA ACA ACC AAG
JQ420904 UUG CAA GCA UUG GAA GUU CAA GCC UCG UUA CAU CAA CCG GAC AAA AUA GAU UAU AAA UUC UUA GCG GGA UUU GCU UUA GGA UUC UCA UCC GCA AUC CCA UUU UCA GUA GCC GGU UUA UAU UUA GUU UAC CUA AAG AUU UCC UCC CAC GUG CGA UCU AUU GUU AAU GAG UAC GGU CGU GGU UAA AGG AAA UGU CAA UGG UGG UGU ACA ACA ACC AAG
JQ420902 UUG CAA GCA UUG GAA GUU UAA GCC UCG UUA CAU CAA CCG GAC AAA AUA GAU UAU AAA UUC UUA GCG GGA UUU GCU UUA GGA UUC UCA UCC GCA AUC CCA UUU UCA GUA GCC GGU UUA UAU UUA GUU UAC CUA AAG AUU UCC UCC CAC GUG CGA UCA AUU GUU AAU GAG UAC GGU CGU GGU UAA AGG UAA UGU CAA UGG UGG UGU ACA ACA ACC AAG
EU717546 UUG CAA GCA UUG GAA GUU CAA GCC UCG UUA CAU CAA CCG GAC AAA AUA GAU UAU AAA UUC UUA GCG GGA UUU GCU UUA GGA UUC UCA UCC GCA AUC CCA UUU UCA GUA GCC GGU UUA UAU UUU GUU UAC CUA AAG AUU UCC UCC CAC GUG CGA UCA AUU GUU AAU GAG UAC GGU CGU GGU UAA AGG AAA UGU CAA UGG UGG UGU ACA ACA ACC AAG
AF453392 UUG CAA GCA UUG GAA GUU CAA GCC UCG UUA CAG CAA CCG GAC AAA AUA GAU UAU AAA UUU CUA GCG GGA UUU GCU UUA GGA UUC UCA UCC GCA AUC CCA UUU UCA GUA GCC GGU UUA UAU UUA GUU UAC CUA AAG AUU UCC UCC CAC GUG CGA UCA AUU GUU AAU GAG UAC GGU CGU GGU UAA AGG AAA UGU CAA UGG UGG UGU ACA ACA ACC AAG
AF453388 UUG CAA GCA UUG GAA GUU CAA GCC UCG UUA CAU CAA CCG GAU AAA AUA GAU UAU AAA UUC UUA GCG GGA UUU GCU UUA GGA UUC UCA UCC GCA AUC CCA UUU UCA GUA GCC GGU UUA UAU UUA GUU UAC CUA AAG AUU UCC UCC CAC GUG AGA UCA AUU GUU AAU GAG UAC GGU CGU GGU UAA GGG AAA UGU CAA UGG UGG UGU ACA ACA ACC AAG
M89926 UUG CAA GCA UUG GAA GUU UAA GCC UCG UUA CAU CAA CCG GAC AAA AUA GAU UAU AAA UUC UUA GCA GGA UUU GCU UUA GGA UUC UCA UCC GCA AUC CCA UUU UCA GUA GCC GGU UUA UAU UUU GUU UAC CUA AAG AUU UCC UCC CAC GUG CGA UCA AUU GUU AAU GAG UAC GGU CGU GGU UAA AGG AAA UGU CAA UGG UGG UGU ACA ACA ACC AAG
AF453391 UUG CAA GCA UUG GAA GUU CAA GCC UCG UUA CAU CAA CCG GAU AAA AUA GAU UAU AAA UUC UUA GCG GGA UUU GCU UUA GGA UUC UCA UCC GCA AUC CCA UUU UCA GUA GCC GGU UUA UAU UUA GUU UAC CUA AAG AUU UCC UCC CAC GUG AGA UCA AUU GGU AAU GAG UAC GGU CGU GGU UAA GGG AAA UGU CAA UGG UGG UGU ACA ACA ACC AAG
AF453390 UUG CAA GCA UUG GAA GUU CAA GCC UCG UUA CAU CAA CCG GAC AAA AUA GAU UAU AAA UUC UUA GCG GGA UUU GCU UUA GGA UUC UCA UCC GCA AUC CCA UUU UCA GUA GCC GGU UUA UAU UUA GUU UAC CUA AGG AUU UCC UCC CAC GUG CGA UCA AUU GUC AAU GAG UAC GGU CGU GGU UAA AGG AAA UGU CAA UGG UGG UGU ACA ACA ACC AAG
JQ346189 UUG CAA GCA UUG GAA GUU CAA GCC UCG UUA CAU CAA CCG GAC AAA AUA GAU UAU AAA UUC UUA GCG GGA UUU GCU UUA GGA UUC UCA UCC GCA AUC CCA UUU UCA GUA GCC GGU UUA UAC UUA GUU UAC CUA AAG AUU UCC UCC CAC GUG CGA GCA AUU GUU AAU GAG UAC GGU CGU GGU UAA AGG AAA UGU CAA UGG UGG UGU ACA ACA ACC AAG
Y07496 UUG CAA GCA UUG GAA GUU CAA GCC UCG UUA CAU CAA CCG GAU AAA AUA GAU UUU AAA UUC UUA GCG GGA UUU GCU UUA GGA UUC UCA UCC GCA AUC CCA UUU UCA GUA GCC GGU UUA UAU UUA GUU UAC CUA AAG AUU UCC UCC CAC GUG CGA UCA AUU GUU AAU GAG UAC GGU CGU GGU UAA AGG AAA UGU CAA UGG CGG UGU ACA ACA ACC AAG
AF453394 UUG CAA GCA UUG GAA GUU CAA GCC UCG UUA CAU CAA CCG GAC AAA AUA GAU UUU AAA UUC UUA GCG GGA UUU GCU UUA GGA UUC UCA UCC GCA AUC CCA UUU UCA GUA GCC GGU UUA UAU UUU GUU UAC CUA AAG AUU UCC UCC CAC GUG CGA UCA AUU GUU AAU GAG UAC GGU CGU GGU UAA AGG AAA UGU CAA UGG UGG UGC ACA CCA ACC AAG
AF453389 UUG CAA GCA UUG GAA GUU CAA GCC UCG UUA CAU CAA CCG GAU AAA AUU GAU UAU AAA UUU UUA GCG GGA UUU GCU UUA GGA UUC UUA UCC GCG AUC CCA UUC UCA GUA GCC GGU UUA UAU UUU GUU UAC CUA AAG AUU UCC UCC CAC GUG CGA UCA AUU GUU AAU GAG UAC GGU CGU GGU UAA AGG AAA UGU CAA UGG UGG UGU ACA ACA ACC AAG
D13953 UUG CAA GCA UUG GAA GUU CAA GCC UCG UUA CAU CAA CCG GAC AAA AUA GAU UAU AAA UUC AUA GCG GGA UUU GCU UCA GGA UUC UCA UCC GCA AUC CCA UUU UCA GUA GCC GGU UUA UAU UUA GUU UAC CUA AAG AUU UCC GAU CAC GUU CGA UCU AUU GUU AAU GAG UAC GGU CGU GGU UAA AGG AAA UGU CAA UGG UGC ACA ACA ACC AAG
NC_004756 GCA AGC AUC GGA AGU CAA GUC UUU CAC AUC AGC CGG ACA CAC AAA AUA GAU UUC AAA UUU CUA GCA GGA UUU GCA UCA GGC UUU CUU GCC GCA AUC CCA UUU UCA GUA GCC GGU AUU UAC UUA GUC UAC CUC AAA AUC UCA -CC CAC GUG CGG AGC AUC GUU AAU GAA UAC GGU CGU GGG UAG AAG AAC AAU CAA UGG AAG AAG ACG ACC ACG CAG
HM804472 GCA AGC GUC GGA AGU CUA GUC UUA CAC AUU AGC CGG ACA CAC AAA AUA GAU UUC AAG UUU CUA GCA GGA UUU GCA UCA GGC UUU CUU GCC GCA AUC CCA UUU UCA GUA GCC GGC CUU UAC UUA GUC UAC CUC AAA AUC UCA GCC CAC GUG CGA AGC AUU GUU AAU GAA UAC GGU CGU GGG UAG AAG GAC AAU CAA UGG GAG AAG ACG ACC ACG AAG
HM804471 GCA AGC GUC GGA AGU CUA GUC UUA CAC AUU AGC CGG ACA CAC AAA AUA GAU UUC AAG UUU CUA GCA GGA UUU GCA UCA GGC UUU CUU GCC GCA AUC CCA UUU UCA GUA GCC GGC CUU UAC UUA GUC UAC CUC AAA AUC UCA GCC CAC GUG CGA AGC AUU GUU AAU GAA UAC GGU CGU GGG UAG AAG GAC AAU CAA UGG GAG AAG ACG ACC ACG AAG
KC210049 UGC AAG CAU CGG AAG UUA AGC CUU UCA CAU UUG CCG GAC AUC ACA AUA GAU UAC AAG UUU CUA GCA GGA UUU GCC ACA GGC UUU CUA GCC GCA AUC CCA UUU UCA GUA GCC GGC AUA UAC AUA GUC UAC CUG AAA AUC UCA ACC CAC GUG CGA AGC AUC GUU AAU GAA UAC GGU CGU GGG UAG AAG AAC AAU CAA UGG AAG AAG ACG ACC ACG GAG
EU636991 CAA GCA UCG GAA GUC AAG CCU UUC ACA UCA GCC GGA CAC UAC AAA AUA GAU UAC AAA UUU CUA GCA GGA UUU GCA ACA GGC UUC CUA GCC GCA AUC CCA AUC UCA GUA GCC GGC AUC UAC AUA GUC UAC CUU AAA AUC UCA ACC CAC GUG CGA AGC AUC GUU AAU GAA UAC GGU CGU GGG UAG GAG AAC AAU CAA UGG ACG AAG ACG ACC ACG CAG
EU636990 CAA GCA UCG GAA GUC AAG CCU UUC ACA UCA GCC GGA CAC UAC AAG AUA GAU UUC AAA UUU CUA GCA GGA UUC GCA UCA GGC UUC CUA GCC GCA AUC CCA UUU UCA GUA GCC GGC AUC UAC UUA GUC UAC CUU AAA AUC UCA ACC CAC GUG CGA AGC AUC GUU AAU GAA UAC GGU CGU GGG UAG GAG AAC AAU CAA UGG ACG AAG ACG ACC ACG CAG
NC_010732 UUG CRA GCG UUG GAA GUU CAA GUC UGA UUA CCA AAG CCC GAC ACC AUA GAU UUU AAA UUU UUA GCA GGA UUU GCG UCA GGA UUU CUA UCC GCA AUC CCA AUU UCA GUU GCG GGC AUU UAC UUA GUC UAC CUC AAA AUC UCA GCC CAC GUU AGA GCU AUU GUU AAU GAA UAC GGG AGG AGC CAG GAG UAA UAA UGG AAA UGG UGG AUC ACG AGU CUC
AF402621 UUG CAA GCG UUG GAA GUU CAA GUC UGA UUA CCA AAG CCC GAC ACC AUA GAU UUU AAA UUU UUA GCA GGA UUU GCG UCA GGA UUU CUA UCC GCA AUC CCA AUU UCA GUU GCG GGC AUU UAC UUA GUC UAC CUC AAA AUC UCA GCC CAC GUU AGA GCU AUU GUU AAU GAA UAC GGG AGG AGU CAG GAG UAA UAA UGG AAA UGG UGG AUC ACG AGU CUC
AJ704819 UUG CAA GCG UUG GAA GUU CAA GUC UGA UUA CCA AAG CCC GAC ACC AUA GAU UUU AAA UUU UUA GCA GGA UUU GCG UCA GGA UUU CUA UCC GCA AUC CCA AUU UCA GUU GCG GGC AUU UACUUUA GUC UAC CUU AAA AUC UCA GCC CAC GUU AGA GCU AUU GUU AAU GAA UAC GGG AGG AGU CAG GAG UAA UAA UGG AAA UGG UGG AUC ACG AGU CUC
JX427533 UGC AAG CGU UGG AAG UUC AAG UCU GAU UAC CAA CAG CCU GAC ACA AUA GAU UUU AAA UUU UUA GCA GGA UUU GCG UUC GGA UUU CUA UCC GCA AUC CCA AUU UCG AUA GCA GGC AUA UAC UUA GUC UAC CUU AAA AUC UCA GCC CAC GUA CGC GCA AUC GUU AAU GAA UAC GGG AGG GGU UAG GAG CAA UAA UAA UGG AAA UGG UGG AUC ACG UAA
JX427532 UUG CAA GCG UUG GAA GUU CAA GUC UGA UAC CAA CAG CCU GAC ACA AUA GAU UUU AAA UUU UUA GCA GGA UUU GCG UUU GGA UUU CUA UCC GCA AUC CCA AUU UCG AUA GCA GGC AUA UAC UUA GUC UAC CUU AAA AUC UCA GCC CAC GUA CGC GCA AUC GUU AAU GAA UAC GGG AGG GGU UAG GAG UAA UAA UAA UGG AAA UGG UGG AUC ACG UAA
FN600344 UGC AAG CGU UGG AAG UUC AAG UCU GAU UAC CAA CAG CCU GAC ACA AUA GAU UUU AAA UUU UUA GCA GGA UUU GCG UUU GGA UUU CUA UCC GCA AUC CCA AUU UCG AUA GCA GGC AUA UAC CUA GUC UAC CUG AAA AUC UCA GCC CAC GUA CGC GCA AUC GUU AAU GAA UAC GGG AGG AGU UAG GAG UAA CAA UAA UGG AAA UGG UGG AUC ACG CAG
JX427534 UGC AAG CAU UGG AAG UUC AAG UCU GAU UAC CAA UAG CCU GAC ACA AUA GAU UUU AAG UUU UUA GCA GGA UUU GCG UUU GGA UUU CUA UCC GCA AUC CCA AUU UCG AUA GCA GGC AUA UAC CUA GUC UAC CUG AAA AUC UCA GCC CAC GUA CGC GCA AUC GUU AAU GAA UAC GGG AGG AGU UAG GAG UAA UAA AAA UGG AAA UGG UGG AUC ACG CAA
JX427537 GCA AGC GUU GGA AAG UUC AAG UCU GAU UAC CAA CAG CCU GAC ACA AUA GAU UUU AAA UUU UUA GCA GGA UUU GCG UUU GGA UUC UUA UCC GCA AUC CCA AUU UCU GUA GCA GGC AUA UAC CUA GUC UAC CUU AAA AUC UCA GCC CAC GUA CGC GCG AUC GUU AAU GAA UAC GGG AGG AGU UAA GAG AAA CAA UAA UGG AAA UGG UGG AUC ACG UAA
JX427541 UGC AAG CGU UGG AAG UUC AAG UCU GAU UAC CAA CAG CCU GAC ACA AUA GAU UAU AAA UUU UUA GCA GGA UUU GCG UUU GGA UUC UUA UCC GCA AUC CCA AUU UCU GUA GCA GGC AUA UAC CUA GUC UAC CUU AAA AUC UCA GCC CAC GUA CGC GCG AUC GUU AAU GAA UAC GGG AGG AGU UAG GAG AAA CAA UAA UGG AAA UGG UGG AUC ACG UAA
JX427539 UGC AAG CGU UGG AAG UUC AAG UCU GAU UAC CAA CAG CCU GAC ACA AUA GAU UAU AAA UUU UUA GCA GGA UUC GCG UUU GGA UUC UUA UCC GCA AUC CCA AUU UCU GUA GCA GGC AUA UAC UUA GUC UAC CUU AAA AUC UCA GCC CAC GUA CGC GCG AUC GUU AAU GAA UAC GGG AGG AGU UAG GAG AAA CAA UAA UGG AAA UGG UGG AUC ACG UAA
JX427536 GCA AGC GUU GGA AAG UUU UAG UCU GAU UAC CAG CAG CCU GAC ACA AUA GAU UAU AAG UUU UUA GCA GGA UUU GCG UUU GGA UUU UUA UCC GCA AUC CCA AUU UCU GUA GCA GGC AUA UAC CUA GUC UAC CUU AAG AUC UCA GCC CAC GUC CGU GCG AUC GUU AAU GAA UAC GGG AGG AGU UAG GAG AAA CAA UAA UGG AAA UGG UGG AUC ACG UAA
JX427535 GCA AGC GUU GGA AAG UUU UAG UCU GAU UAC CAG CAG CCU GAC ACA AUA GAU UAU AAG UUU UUA GCA GGA UUU GCG UUU GGA UUU UUA UCC GCA AUC CCA AUU UCU GUA GCA GGC AUA UAC CUA GUC UAC CUU AAG AUC UCA GCC CAC GUC CGU GCG AUC GUU AAU GAA UAC GGG AGG AGU UAG GAG AAA CAA UAA UGG AAA UGG UGG AUC ACG UAA
JX427531 UGC AAG CGU UGG AAG UUC AAG UCU GAU UAC CAA CAG CCU GAC ACA AUA GAU UAC AAA UUU UUA GCA GGA UUU GCG UUU GGA UUC UUA UCC GCA AUC CCA AUU UCU GUA GCA GGC AUA UAC CUA GUC UAC CUU AAA AUC UCA GCC CAC GUA CGC GCG AUC GUU AAU GAA UAC GGG AGG AGU UAG GAG AAA CAA UAA UGG AAA UGG UGG AUC ACG UAA
JX427540 UGC AAG CGU UGG AAG UUC AAG UCU GAU UAC CAA CAG CCU GAC ACA AUA GAU UAU AAA UUU UUA GCA GGA UUC GCG UUU GGA UUC UUA UCC GCA AUC CCA AUU UCU GUA GCA GGC AUA UAC CUA GUC UAC CUU AAA AUC UCA GCC CAC GUA CGC GCG AUC GUU AAU GAA UAC GGG AGG AGU UAG GAG AAA CAA UAA UGG AAA UGG UGG AUC ACG UAA
JX427538 UGC AAG CGU UGG AAG UUC AAG UCU GAU UAC CAA CAG CCU GAC AUA AUA GAU UAU AAA UUU UUA GCA GGA UUC GCG UUC GGA UUU UUA UCC GCA AUC CCA AUU UCU GUA GCA GGC AUA UAC CUA GUC UAC CUU AAG AUC UCA GCC CAC GUU CGC GCG AUC GUU AAU GAA UAC GGG AGG AGU UAG GAG CAA UAA UAA UGG AAA UGG UGG AUC ACG UAG
JX427542 GCA AGC GUU GGA AAG UUC UAG UCU GAU UAC CAA CAG CCU GAC AUA AUA GAU UAU AAA UUU UUA GCA GGA UUU GCG UUU GGA UUU UUA UCC GCA AUC CCA AUU UCU GUA GCA GGC AUA UAC CUA GUC UAC CUU AAA AUC UCA GCC CAC GUA CGU GCA AUC GUU AAU GAA UAC GGG AGG AGU UAG GAG AAA CAA UAA UGG AAA UGG UGG AUC ACG UAG
NC_015050 GCA AGC GUU GGA AAG UUU UAG UCU GAU UAC CAA CAG CCU GAC AUA AUA GAU UAU AAA UUU UUA GCA GGA UUU GCG UUU GGA UUC UUA UCC GCA AUC CCA AUU UCU GUA GCA GGC AUA UAC CUA GUC UAC CUU AAA AUC UCA GCC CAC GUU CGC GCA AUC GUU AAU GAA UAC GGG AGG GGU UAG GAG AAA UAA UAA UGG AAA UGG UGG AUC ACG UAA
JX427543 GCA AGC GUU GGA AAG UUC AAG UCU GAU UAC CAA CAG CCU GAC AUA AUA GAU UAU AAA UUU UUA GCA GGA UUU GCG UUU GGA UUC UUA UCC GCA AUC CCA AUU UCU GUA GCA GGC AUA UAC CUA GUC UAC CUU AAA AUC UCA GCC CAC GUA CGC GCA AUC GUU AAU GAA UAC GGG AGG GGU UAG GAG AAA CAA UAA UGG AAA UGG UGG AUC ACG UAA
HM439608 UGC AAG CGU UGG AAG UUC AAG UCU GAU UAC CAA CAG CCU GAC ACA AUA GAU UAC AAA UUU UUA GCA GGA UUU GCG UCU GGA UUU CUU UCC GCA AUC CCA CUU UCU GUA GCA GGC AUU UAC UUA GUC UAC CUU AAA AUC UUA GCC CACGUUC CGC GCG AUC GUU AAU GAA UAC GGA AGG GGU UAG GAG AAA UAA UAA UGG AAA UGG UGG AUC ACG CGG
KC121026 UUG CAA ACG CCG GAA GUU UAA AGU CUG AUU ACA UAA CAA GCC AAA AUA GAU UUC AAG UUU UUA GCA GGA UUU UCA AGU GGU CUA UUG UCA GCA AUA CCU GUA ACG GUA GUU GGC UUG UAU UUC GUC UAC CUU AAG AUU UCU CAC CAC GUC AGA UCA AUU GUU AAU GAA UAC GGU CGU GGG UAG GAG AAC GAU CAA UGG AAG AAG ACG ACC ACG UAG
NC_003491 UUG CAA ACG CCG GAA GUU UAA AGU CUG AUU ACA UAA CAA GCC AAA AUA GAU UUC AAG UUU UUA GCA GGA UUU UCA AGU GGU CUA -UG UCA GCA AUA CCU GUA ACG GUA GUU GGC UUG UAU UUC GUC UAC CUU AAG AUU UCU CAC CAC GUC AGA UCA AUU GUU AAU GAA UAC GGU CGU GGG UAG GAG AAC GAU CAA UGG AAG AAG ACG ACC ACG UAG
NC_002766 GUU GCA AAC ACC GGA AGU UUU AGU CUG AUU ACA CAA CAA GCC AAA AUA GAC UUC AAA UUU UUA GCA GGA UUU UCA AGC GGU CUA -UG UCA GCA AUA CCC GUA ACG GUA AUU GGC UUG UAU UUU GUC UAC CUG AAA AUC UCC CAA CAC GUA AGA UCA AUC GUU AAU GAA UAC GGU CGU GGG UAG GAG AAC AAU CAA UGG AAG AAG ACG ACC ACG AAG
EF107543 GUU GCA AAC GCC GGA AGU UUA AAU CUG AUU ACA UAA CAA GCC AAA AUA GAU UUC AAG UUU UUA GCA GGA UUU UCA AGU GGU CUA UUG UCA GCA AUA CCU GUA ACG G-- GUU GGC UUG UAU UUC GUC UAC CUU AAG AUU UCU CAC CAC GUC AGA UCA AUU GUU AAU GAA UAC GGU CGU GGG UAG GAG AAC GAU CAA UGG AAG AAG ACG ACC ACG UAG
DQ132996 UUG CAA ACG CCG GAA GUU UAA AGU CUG AUU ACA UAA CAA GCC AAA AUA GAU UUC AAG UUU UUA GCA GGA UUU UCA AGU GGU CUA UUG UCA GCA AUA CCU GUA ACG GUA GUU GGC UUG UAU UUC GUC UAC CUG AAG AUU UCU CAC CAC GUC AGA UCA AUU GUU AAU GAA UAC GGU CGU GGG UAG GAG AAC GAU CAA UGG AAG AAG ACG ACC ACG UAG
L39952 GUU GCA AAC ACC GGA AGU UUU AGU CUG AUU ACA CAA CAA GCC AAA AUA GAC UUC AAA UUU UUA GCA GGA UUU UCA AGC GGU CUA UUG UCA GCA AUA CCC GUA ACG GUA AUU GGC UUG UAU UUU GUC UAC CUG AAA AUC UCC CAA CAC GUA AGA UCA AUC GUU AAU GAA UAC GGU CGU GGG UAG GAG AAC AAU CAA UGG AAG AAG ACG ACC ACG AAG
AF352025 GUU GCA AAC ACC GGA AGU UUU AGU CUG AUU ACA CAA CAA GCC AAA AUA GAC UUC AAA UUU UUA GCA GGA UUC UCA AGC GGU CUA UUG UCA GCA AUA CCC GUA ACG GUA AUC GGC UUG UAU UUU GUC UAC CUG AAA AUC UCC CAA CAC GUA AGA UCA AUC GUU AAU GAA UAC GGU CGU GGG UAG GAG AAC AAU CAA UGG AAG AAG ACG ACC ACG CAG
L40019 GUU GCA AAC ACC GGA AGU UUU AGU CUG AUU ACA UAA CAA GCC AAA AUA GAC UUC AAA UUU UUA GCA GGA UUU UCA AGC GGU CUA UUG UCA GCA AUA CCC GUG ACG GUA AUU GGC UUG UAU UUU GUC UAC CUG AAA AUC UCC CAA CAC GUA AGA UCA AUC GUU AAU GAA UAC GGU CGU GGG UAG GAG AAC AAU CAA UGG AAG AAG ACG ACC ACG AAG
L39985 GUU GCA AAC ACC GGA AGU UUU AGU CUG AUA ACA UAA CAA GUC AAA AUA GAC UUC AAA UUU UUA GCA GGA UUU UCA AGC GGU CUA UUG UCA GCA AUA CCC GUG ACG GUA AUU GGC UUG UAU UUU GUC UAC CUG AAA AUC UCC CAA CAC GUA AGA UCA AUC GUU AAU GAA UAC GGU CGU GGG UAG GAG AAC AAU CAA UGG AAG AAG ACG ACC ACG AAG
L40020 GUU GCA AAC ACC GGA AGU UUU AGU CUG AUU ACA UAA CAA GCC AAA AUA GAC UUC AAA UUU UUA GCA GGA UUU UCA AGC GGU CUA UUG UCA GCA AUA CCC GUG ACG GUA AUU GGC UUG UAU UUU GUC UAC CUG AAA AUC UCC CAA CAC GUA AGA UCA AUC GUU AAU GAA UAC GGU CGU GGG UAG GAG AAC AAU CAA UGG AAG AAG ACG ACC ACG AAG
L39984 GUU GCA AAC ACC GGA AGU UUU AGU CUG AUU ACA UAA CAA GCC AAA AUA GAC UUC AAA UUU UUA GCA GGA UUU UCA AGC GGU CUA UUG UCA GCA AUA CCC GUG ACG GUA AUU GGC UUG UAU UUU GUC UAC CUG AAA AUC UCC CAA CAC GUA AGA UCA AUC GUU AAU GAA UAC GGU CGU GGG UAG GAG AAC AAU CAA UGG AAG AAG ACG GCG AAG
JQ700306 GUU GCA AGC GUU GGA AGU CUA GUC UCG UUA CAA AGA GUC CAC ACA AUA GAC UAC AAA UUU CUC GCA GGG UUU UCC AGC GGU CUA UUG UCC GCA AUA CCU GUC ACG GUA AUA GGA CUC UAU UUU GUC UAC CUA AAG AUU UCA GCA CAC GUG CGA GCC AUC GUU AAU GAA UAC GGC CGU GGC UAG AAA UCA AAA UGC AGG GAG GCG CAG GCG ACG AAA
JQ700305 UUG CAA GCA UUG GAA GUC UGA GUC UCG UUA CCA AAA GUC CAA ACA AUA GAU UAU AAG UUU CUC GCA GGA UUU UCU AGC GGU CUA UUG UCU GCA AUA CCU GUA ACG GUA AUU GGA CUU UAU UUU GUC UAC CUA AAG AUU UCA GCA CAC GUG CGA GCC AUU GUU AAU GAA UAC GGC CGU GGC UAG AAA UCA AAA UGC AGG GAG GCG AAG GCG AAG AAA
GU324102 GUU GCA AGC GUU GGA AGU CUA GUC UCG UUA CAA AGA GUC CAC ACA AUA GAC UAC AAA UUU CUC GCA GGG UUU UCC AGC GGU CUA UUG UCC GCA AUA CCU GUC ACG GUA AUA GGA CUC UAU UUU GUC UAC CUA AAG AUU UCA GCA CAC GUG CGA GCC AUC GUU AAU GAA UAC GGC CGU GGC UAG AAA UCA AAA UGC AGG GAG GCG CAG GCG ACG AAA
GU324095 UUG CAA GCA UUG GAA GUC UGA GUC UCG UUA CCA AGA GUC CAA ACA AUA GAU UAU AAG UUU CUC GCA GGA UUU UCU AGC GGU CUA UUG UCU GCA AUA CCU GUA ACG GUA AUU GGA CUU UAU UUU GUC UAC CUA AAG AUU UCA GCA CAC GUG AGA GCC AUU GUU AAU GAA UAC GGC CGU GGC UAG AAA UCA AAA UGC AGG GAG GCG AAG GCG AAG AAA
GU324104 UUG CAA GCA CUG GAA GUU UAA GUC UCG UUA CCA AGA GUC CAA ACA AUA GAU UAU AAG UUU CUC GCA GGA UUU UCU AGC GGU CUA UUG UCU GCA AUA CCU AUC ACG GUA AUC GGA CUC UAU UUU GUC UAC CUA AAG AUU UCA GCA CAC GUG CGA GCA AUU GUU AAU GAA UAC GGC CGU GGC UAG AAA UCA AAA UGC AGG GAG GCG CAG GCG ACG AAA
GU324103 UUG CAA GCA CUG GAA GUU UAA GUC UCG UUA CCA AGA GUC CAA ACA AUA GAU UAU AAA UUU CUC GCA GGA UUU UCU AGC GGU CUA UUG UCU GCA AUA CCU AUC ACG GUA AUC GGA CUC UAU UUU GUC UAC CUA AAG AUU UCA GCA CAC GUG CGA GCA AUU GUU AAU GAA UAC GGC CGU GGC UAG AAA UCA AAA UGC AGG GAG GCG CAG GCG ACG AAA
GU324101 UUG CAA GCA CUG GAA GUU UAA GUC UCG UUA CCA AGA GUC CAA ACA AUA GAU UAU AAA UUU CUC GCA GGA UUU UCU AGC GGU CUA UUG UCU GCA AUA CCU AUC ACG GUA AUC GGA CUC UAU UUU GUC UAC CUA AAG AUU UCA GCA CAC GUG CGA GCA AUU GUU AAU GAA UAC GGC CGU GGC UAG AAA UCA AAA UGC AGG GAG GCG CAG GCG ACG AAA
GU324099 UUG CAA GCA CUG GAG GUU UAA GUC UCG UUA CCA AGA GUC CAA ACA AUA GAU UAU AAA UUU CUC GCA GGA UUU UCU AGC GGU CUA UUG UCU GCA AUA CCU AUC ACG GUA AUC GGA CUC UAU UUU GUC UAC CUA AAG AUU UCA GCA CAC GUG CGA GCA AUU GUU AAU GAA UAC GGC CGU GGC UAG AAA UCA AAA UGC AGG GAG GCG CAG GCG ACG AAA
GU324098 UUG CAA GCA CUG GAA GUU UAA GUC UCG UUA CCA AGA GUC CAA ACA AUA GAU UAU AAA UUU CUC GCA GGG UUU UCU AGC GGU CUA UUG UCU GCA AUA CCU AUC ACG GUA AUC GGA CUC UAU UUU GUC UAC CUA AAG AUU UCA GCA CAC GUG CGA GCA AUU GUU AAU GAA UAC GGC CGU GGC UAG AAA UCA AAA UGC AGG GAG GCG CAG GCG ACG AAA
GU324097 GUU GCA AGC GUU GGA AGU CUA GUC UCG UUA CAA AGA GUC CAA ACA AUA GAU UAU AAA UUU CUC GCA GGA UUU UCU AGC GGU CUA UUG UCU GCA AUA CCU AUC ACA GUA AUC GGA CUC UAU UUU GUC UAC CUA AAG AUU UCA GCA CAC GUG CGA GCA AUU GUU AAU GAA UAC GGC CGU GGC UAG AAA UCA AAA UGC AGG GAG GCG CAG GCG ACG AAA
GU324096 UUG CAA GCA CUG GAA GUU UGA GUC UCG UUA CCA AGA GUC CAA ACA AUA GAU UAU AAA UUU CUC GCA GGA UUU UCU AGC GGU CUA UUG UCU GCA AUA CCU AUU ACG GUA AUC GGA CUC UAU UUU GUC UAC CUA AAG AUU UCA GCA CAC GUG CGA GCA AUU GUU AAU GAA UAC GGC CGU GGC UAG AAA UCA AAA UGC AGG GAG GCG CAG GCG ACG AAA
HQ700823 UUG CAA GCG UUG GAA GUA UAA GUC UCG UUA CAA AGA GUC CAC ACA AUA GAU UAU AAA UUU CUC GCA GGA UUU UCU AGC GGU CUA UUG UCU GCU AUA CCC AUC ACG GUA AUA GGA CUC UAU UUU GUC UAC CUA AAG AUU UCA GCA CAC GUG CGA GCA AUU GUU AAU GAA UAC GGC CGU GGU UAG AAA UCA AAA UGC AGG GAG GCG CAG GCG ACG AAA
GU324100 UUG CAA GCA UCG GAA GUC UGA GUC UCG UUA CCA AGA GUC CAC ACA AUA GAU UAU AAG UUU CUC GCA GGA UUU UCA AGC GGU CUA UUG UCA GCA AUA CCC AUC ACG GUG AUU GGG CUC UAU UUU GUC UAC CUA AAG AUU UCA GCC CAC GUU CGA UCA AUU GUU AAU GAA UAC GGU CGU GGC UAG AAA UCC AAA UGC AGG GAG GCG AAG GCG AAG AAA
NC_003688 UUG CAA GCA UUG GAA GUC UGA GUC UCG UUA CCA AGA GUC CAC ACA AUA GAU UAU AAG UUU CUC GCA GGA UUU UCA AGC GGU CUA UUG UCU GCA AUA CCC AUC ACG GUA AUC GGA CUC UAU UUU GUC UAC CUA AAG AUU UCA GCA CAC GUG CGU UCA AUU GUU AAU GAA UAC GGU CGC GGC UAG AAA UCA AAA UGC AGG GAG GCG AAG GCG AAG AAA
JF939814 CUG CAA GCA UUG GAA GUC UGA GUC UCG UUA CCA AGA GUC CAC ACA AUA GAU UAU AAG UUU CUC GCA GGA UUU UCA AGC GGU CUA UUG UCU GCA AUA CCU AUU ACG GUA AUC GGA CUC UAU UUU GUC UAC CUA AAG AUU UCA GCA CAC GUG CGC UCA AUU GUU AAU GAA UAC GGC CGC GGC UAG AAA UCA AAA UGC AGG GAG GCG AAG GCG AAG AAA
JF939812 UUG CAA GCA UUG GAA GUC UGA GUC UCG UUA CUA AGA GUC CAC ACA AUA GAU UAU AAG UUU CUC GCA GGA UUU UCA AGC GGU CUA UUG UCU GCA AUA CCC AUC ACG GUA AUC GGA CUC UAU UUU GUC UAC CUA AAG AUU UCA GCA CAC GUA CGC UCA AUU GUU AAU GAA UAC GGU CGC GGC UAG AAA UCA AAA UGC AGG GAG GCG AAG GCG AAG AAA
JF939813 UUG CAA GCA UUG GAA GUA UAA GUC UCG UUA CCA AGA GUC CAC ACA AUA GAU UAC AAA UUU CUC GCA GGA UUU UCA AGC GGU CUA UUG UCU GCU AUA CCC AUC ACG GUG AUU GGA CUC UAU UUU GUC UAC CUA AAG AUU UCG UCA CAC GUG CGA UCA AUU GUU AAU GAA UAC GGU CGU GGC UAG AAA UCA AAA UGC AGG GAG GCG AAG GCG GAG AAA
HQ700824 UUG CAA GCG UUG GAA GUU AAA GCC UCG UUA CAA AGA GUC CAC ACA AUA GAU UAU AAA UUU CUC GCA GGA UUU UCU AGC GGU CUA UUG UUA GCG AUA CCU GUA ACG GUA GUA GGA CUC UAU UUU GUC UAC CUA AAG AUU UCA GCA CAC GUG CGA GCA AUC GUU AAU GAA UAC GGC CGU GGC UAG AAA UCA AAA UGC AGG GAG GCG CAG GCG ACG AAA
EU244327 UUG CAA GCA UUG GAA GUC UGA GUC UCG UUA CCA AGA GUC CAC ACA AUA GAU UAC AAA UUU CUC GCA GGA UUU UCU AGC GGU CUA UUG UUA GCA AUA CCU GUC ACG GUA AUU GGA CUC UAU UUU GUC UAC CUA AAG AUU UCA GCC CAC GUG CGA UCA AUU GUU AAU GAA UAC GGU CGU GGU UAG AAA UCA AAA UGC AGG GAG GCG GAG GCG AAG AAA
EU244326 UUG CAA GCA UUG GAA GUC UGA GUC UCG UUA CCA AGA GUC CAC ACA AUA GAU UAC AAA UUU CUC GCA GGA UUU UCU AGC GGU CUA UUG UUA GCU AUA CCU GUC ACG GUA AUU GGA CUC UAU UUU GUC UAC CUA AAG AUU UCG GCC CAC GUG CGA UCA AUA GUU AAU GAA UAC GGU CGU GGU UAG AAA UCA AAA UGC AGG AAG GCG GAG GCG AAG AAG
EF488997 UUG CAA GCA UUG GAA GUC UGA GUC UCG UUA CCA AGA GUC CAC ACA AUA GAU UAC AAA UUU CUC GCA GGA UUU UCU AGC GGU CUA UUG UUA GCA AUA CCU GUC ACG GUA AUU GGA CUC UAU UUU GUC UAC CUA AAG AUU UCA GCC CAC GUG CGA UCA AUU GUU AAU GAA UAC GGC CGC GGU UAG AAA UCA AAA UGC AGG GAG GCG GAG GCG AAG AAA
GQ221224 UUG CAA GCA UUG GAA GUA UAA GUC UCG UUA CCA AGA GUC CAC ACA AUA GAU UAC AAA UUU CUC GCA GGA UUU UCU AGC GGU CUA UUG UCU GCA GUA CCA GUU ACG GUA AUA GGA CUC UAU UUU GUC UAC CUA AAG AUU UCA GCA CAC GUG CGC UCA AUU GUU AAU GAA UAC GGC CGC GGC UAG AAA UCA AAA UGC AGG GAG GCG GAG GCG AAG AAA
EU244318 UUG CAA GCA UUG GAA GUA UAA GUC UCG UUA CCA AGA GUC CAC ACA AUA GAU UAC AAA UUU CUC GCA GGA UUU UCU AGC GGU CUA UUG UCU GCA GUG CCA GUU ACG GUA AUA GGA CUC UAU UUU GUC UAC CUA AAG AUU UCA GCA CAC GUG CGA UCA AUU GUU AAU GAA UAC GGC CGU GGC UAG AAA UCA AAA UGC AGG GAG GCG GAG GCG AAG AAA
EU244321 UUG CAA GCA UUG GAA GUA UAA GUC UCG UUA CCA AGA GUC CAC ACA AUA GAU UAC AAA UUU CUC GCA GGA UUU UCU AGC GGU CUA UUG UCU GCA GUG CCA GUU ACG GUA AUA GGA CUC UAU UUU GUC UAC CUA AAG AUU UCA GCA CAC GUG CGA UCA AUU GUU AAU GAA UAC GGC CGC GGC UAG AAA UCA AAA UGC AGG GAG GCG GAG GCG AAG AAA
EU244323 UUG CAA GCA UUG GAA GUA UAA GUC UCG UUA CCA AGA GUC CAC ACA AUA GAU UAC AAA UUU CUC GCA GGA UUU UCU AGC GGU CUA UUG UCU GCA GUG CCA GUU ACG GUA AUA GGA CUC UAU UUU GUC UAC CUA AAG AUU UCA GCA CAC GUG CGA UCA AUU GUU AAU GAA UAC GGC CGC GGC UAG AAA UCA AAA UGC AGG GAG GCG GAG GCG AAG AAA
EU244320 UUG CAA GCA UUG GAA GUA UAA GUC UCG UUA CCA AGA GUC CAC ACA AUA GAC UAC AAA UUU CUC GCA GGA UUU UCU AGC GGU CUA UUG UCU GCA GUG CCA GUU ACG GUA AUA GGA CUC UAU UUU GUC UAC CUA AAG AUU UCA GCA CAC GUG CGA UCA AUU GUU AAU GAA UAC GGC CGC GGC UAG AAA UCA AAA UGC AGG GAG GCG GAG GCG AAG AAA
EU244319 UUG CAA GCA UUG GAA GUA CAA GUC UCG UUA CCA AGA GUC CAC ACA AUA GAU UAU AAA UUU CUC GCA GGA UUU UCU AGC GGU CUA UUG UCU GCA GUA CCA GUC ACG GUA AUA GGA CUC UAU UUU GUC UAC CUA AAG AUU UCA GCA CAC GUG CGA UCA AUU GUU AAU GAA UAC GGU CGC GGC UAG AAA UCA AAA UGC AGG GAG GCG GAG GCG AAG AAA
EU244317 UUG CAA GCA UUG GAA GUA UAA GUC UCG UUA CUA AGA GUC CAC ACA AUA GAU UAC AAA UUU CUC GCA GGA UUU UCU AGC GGU CUA UUG UCU GCA GUG CCA GUU ACG GUA AUA GGA CUC UAU UUU GUC UAC CUA AAG AUU UCA GCA CAC GUG CGA UCA AUU GUU AAU GAA UAC GGC CGU GGU UAG AAA UCA GAA UGC AGG GAG GCG GAG GCG AAG AAA
EU244315 UUG CAA GCG UUG GAA GUA CAA GUC UCG UUA CCA AGA GUC CAC ACA AUA GAU UAC AAA UUU CUC GCA GGA UUU UCU AGC GGU CUA UUG UCU GCA GUA CCA GUU ACG GUA AUA GGA CUC UAU UUU GUC UAC CUA AAG AUU UCA GCA CAC GUG CGA UCA AUU GUU AAU GAA UAC GGC CGU GGC UAG AAA UCA AAA UGC AGG GAG GCG GAG GCG AAG AAA
EU091148 UUG CAA GCA UUG GAA GUA CAA GUC UCG UUA CCA AGA GUC CAC ACA AUA GAU UAC AAA UUU CUC GCA GGA UUU UCU AGC GGU CUA UUG UCU GCA GUA CCA GUU ACG GUA AUC GGA CUC UAU UUU GUC UAC CUA AAG AUU UCA GCA CAC GUG CGA UCA AUU GUU AAU GAA UAC GGC CGC GGC UAG AAA UCA AAA UGC AGG GAG GCG GAG GCG AAG AAA
EU000535 UUG CAA GCA UUG GAA GUA UAA GUC UCG UUA CUA AGA GUC CAC ACA AUA GAU UAC AAA UUU CUC GCA GGA UUU UCU AGC GGU CUA UUG UCU GCA GUG CCA GUU ACG GUA AUA GGA CUC UAU UUU GUC UAC CUA AAG AUU UCA GCA CAC GUG CGA UCA AUC GUU AAU GAA UAC GGC CGU GGC UAG AAA UCA AAA UGC AGG GAG GCG GAG GCG AAG AAA
HQ439023 UUG CAA GCA UUG GAA GUA UAA GUC UCG UAA CCA AGA GUC CAC ACA AUA GAU UAC AAA UUU CUC GCA GGA UUU UCU AGC GGU CUA UUG UCU GCA GUG CCA GUU ACG GUA AUA GGA CUC UAU UUU GUC UAC CUA AAG AUU UCA GCA CAC GUG CGA UCA AUU GUU AAU GAA UAC GGC CGU GGC UAG AAA UCA AAA UGC AGG GAG GCG GAG GCG AAG AAA
FJ425879 UUG CAA GCA UUG GAA GUA UAA GUC UCG UUA CCA AGA GUC CAC ACA AUA GAU UAC AAA UUU CUC GCA GGA UUU UCU AGC GGU CUA UUG UCU GCA GUA CCA GUU ACG GUA AUA GGA CUC UAU UUU GUC UAC CUA AAG AUU UCA GCA CAC GUG CGC UCA AUU GUU AAU GAA UAC GGC CGC GGC UAG AAA UCA AAA UGC AGG GAG GCG GAG GCG AAG AAA
EU244325 UUG CAA GCA UUG GAA GUA UAA GUC UCG UUA CCA AGA GUC CAC ACA AUA GAU UAU AAA UUU CUC GCA GGA UUU UCU AGC GGU CUA UUG UCA GCA GUA CCA GUG ACG GUA AUA GGA CUC UAU UUU GUC UAC CUA AAG AUU UCA GCG CAC GUG CGA UCA AUU GUU AAU GAA UAC GGC CGC GGC UAG AAA UCA AAA UGC AGG GAG GCG GAG GCG AAG AAG
EU262631 UUG CAA GCA UUG GAA GUA UAA GUC UCG UUA CCA AGA GUC CAC ACA AUA GAU UAC AAA UUU CUC GCA GGA UUU UCU AGC GGU CUA UUG UCU GCA GUG CCA GUU ACG GUA AUA GGA CUC UAU UUU GUC UAC CUA AAG AUU UCA GCA CAC GUG CGA UCA AUU GUU AAU GAA UAC GGC CGU GGC UAG AAA UCA AAA UGC AGG GAG GCG GAG GCG AAG AAA
EU262629 UUG CAA GCA UUG GAA GUA UAA GUC UCG UUA CCA AGA GUC CAC ACA AUA GAU UAC AAA UUU CUC GCA GGA UUU UCU AGC GGU CUA UUG UCU GCA GUA CCA GUU ACG GUA AUA GGA CUC UAU UUU GUC UAC CUA AAG AUU UCA GCA CAC GUG CGA UCA AUU GUU AAU GAA UAC GGC CGU GGC UAG GAA UCA AAA UGC AGG GAG GCG GAG GCG AAG AAA
EF063706 UUG CAA GCA UUG GAA GUA UAA GUC UCG UUA CCA AGA GUC CAC ACA AUA GAU UAC AAA UUU CUC GCA GGA UUU UCU AGC GGU CUA UUG UCU GCA GUA CCA GUU ACG GUA AUA GGA CUC UAU UUU GUC UAC CUA AAG AUU UCA GCA CAC GUG CGA UCA AUC GUU AAU GAA UAC GGC CGC GGC UAG AAA UCA AAA UGC AGG GAG GCG GAG GCG AAG AAA
EU244316 UUG CAA GCG UUG GAA GUA CAA GUC UCG UUA CCA AGA GUC CAC ACA AUA GAU UAC AAA UUU CUC GCA GGA UUU UCU AGC GGU CUA UUG UCU GCA GUA CCA GUU ACG GUA AUA GGA CUC UAU UUU GUC UAC CUA AAG AUU UCA GCA CAC GUG CGA UCA AUU GUU AAU GAA UAC GGC CGU GGC UAG AAA UCA AAA UGC AGG GAG GCG GAG GCG AAG AAA
FJ425880 UUG CAA GCA UUG GAA GUA UAA GUC UCG UUA CCA AGA GUC CAC ACA AUA GAU UAC AAA UUU CUC GCA GGG UUU UCU AGC GGU CUA UUG UCU GCA GUG CCA GUC ACG GUA AUA GGA CUC UAU UUU GUC UAC CUA AAG AUU UCA GCA CAC GUG CGA UCA AUU GUU AAU GAA UAC GGC CGU GGC UAG AAA UCA AAA UGC AGG GAG GCG GAG GCG AAG AAA
EU262627 UUG CAA GCA UUG GAA GUA UUA GUC UCG UUA CUA AGA GUC CAC ACA AUA GAU UAC AAA UUU CUC GCA GGA UUU UCU AGC GGU CUA UUG UCU GCA GUA CCA GUU ACG GUG AUA GGA CUC UAU UUU GUC UAC CUA AAG AUU UCA GCA CAC GUG CGA UCA AUU GUU AAU GAA UAC GGC CGC GGC UAG AAA UCA AAA UGC AGG GAG GCG GAG GCG AAG AAA
EU262625 UUG CAA GCA UUG GAA GUU UAA GUC UCG UUA CCA AGA GUC CAC ACA AUA GAU UAC AAA UUU CUC GCA GGA UUU UCU AGC GGU CUA UUG UCU GCA GUA CCA GUU ACG GUA AUA GGA CUC UAU UUU GUC UAC CUA AAG AUU UCA GCA CAC GUG CGA UCA AUU GUU AAU GAA UAC GGC CGC GGC UAG AAA UCA AAA UGC AGG GAG GCG GAG GCG AAG AAA
EU262624 UUG CAA GCA UUG GAA GUA UUA GUC UCG UUA CCA AGA GUC CAC ACA AUA GAU UAC AAA UUU CUC GCA GGA UUU UCU AGC GGU CUA UUG UCU GCA GUA CCA GUU ACG GUG AUA GGA CUC UAU UUU GUC UAC CUA AAG AUU UCA GCA CAC GUG CGA UCA AUU GUU AAU GAA UAC GGC CGC GGC UAG AAA UCA AAA UGC AGG GAG GCG GAG GCG AAG AAA
GQ221223 UUG CAA GCA UUG GAA GUA UAA GUC UCG UUA CCA AGA GUC CAC ACA AUA GAU UAC AAA UUU CUC GCA GGG UUU UCU AGC GGU CUA UUG UCU GCA GUG CCA GUC ACG GUA AUA GGA CUC UAU UUU GUC UAC CUA AAG AUU UCA GCA CAC GUG CGA UCA AUU GUU AAU GAA UAC GGC CGU GGC UAG AAA UCA AAA UGC AGG GAG GCG GAG GCG AAG AAA
FJ460216 UUG CAA GCA UUG GAA GUA UAA GUC UCG UUA CCA AGA GUC CAC ACA AUA GAU UAC AAA UUU CUC GCA GGA UUU UCU AGC GGU UUA UUG UCU GCA GUA CCA GUU ACG GUA AUA GGA CUC UAU UUU GUC UAC CUA AAG AUU UCA GCA CAC GUG CGA UCA AUU GUU AAU GAA UAC GGC CGU GGC UAG AAA UCA AAA UGC AGG GAG GCG GAG GCG AAG AAA
FJ460214 UUG CAA GCA UUG GAA GUA UAA GUC UCG UUA CCA AGA GUC CAC ACA AUA GAU UAC AAA UUU CUC GCA GGA UUU UCU AGU GGU CUA UUG UCU GCA GUA CCA GUU ACG GUA AUA GGA CUC UAU UUU GUC UAC CUA AAG AUU UCA GCA CAC GUG CGA UCA AUU GUU AAU GAA UAC GGC CGU GGC UAG AAA UCA AAA UGC AGG GCG GAG GCG AAG AAA
EU636992 UUG CAA GCG UUG GAA GUA CAA GUC UCG UUA CCA AGA GUC CAC ACA AUA GAU UAC AAA UUU CUC GCA GGA UUU UCU AGC GGU CUA UUG UCU GCA GUA CCA GUU ACG GUA AUA GGA CUC UAU UUU GUC UAC CUA AAG AUU UCA GCA CAC GUG CGA UCA AUU GUU AAU GAA UAC GGC CGU GGC UAG AAA UCA AAA UGC AGG GCG GAG GCG AAG AAA
EU262630 UUG CAA GCA UUG GAA GUA UAA GUC UCG UUA CCA AGA GUC CAC ACA AUA GAU UAC AAA UUU CUC GCA GGA UUU UCU AGC GGU CUA UUG UCU GCA GUA CCA GUU ACG GUA AUA GGA CUC UAU UUU GUC UAC CUA AAG AUU UCA GCA CAC GUG CGA UCA AUU GUU AAU GAA UAC GGC CGU GGC UAG GAA UCA AAA UGC AGG GAG GCG GAG GCG AAG AAA
EU262626 UUG CAA GCA UUG GAA GUA CAA GUC UCG UUA CCA AGA GUC CAC ACA AUA GAU UAC AAA UUU CUC GCA GGA UUU UCU AGC GGU CUA UUG UCU GCA GUA CCA GUU ACG GUA AUA GGA CUC UAU UUU GUC UAC CUA AAG AUU UCA GCA CAC GUG CGA UCA AUU GUU AAU GAA UAC GGC CGU GGC UAG GAA UCA AAA UGC AGG GAG GCG GAG GCG AAG AAA
EU244322 UUG CAA GCA UUG GAA GUA UAA GUC UCG UUA CCA AGA GUC CAC ACA AUA GAU UAC AAA UUU CUC GCA GGA UUU UCU AGC GGU CUA UUG UCU GCA GUA CCA GUU ACG GUA AUA GGA CUC UAU UUU GUC UAC CUA AAG AUU UCA GCA CAC GUG CGA UCA AUU GUU AAU GAA UAC GGC CGU GGU UAG AAA UCA AAA UGC AGG GAG GCG GAG GCG AAG AAA
EU262623 UUG CAA GCA UUG GAA GUA UAA GUC UCG UUA CCA AGA GUC CAC ACA AUA GAU UAC AAA UUU CUC GCA GGA UUU UCU AGC GGU CUA UUG UCU GCA GUG CCA GUU ACG GUA AUA GGA CUC UAU UUU GUC UAC CUA AAG AUU UCA GCA CAC GUG CGA UCA AUU GUU AAU GAA UAC GGC CGU GGC UAG AAA UCA AAA UGC AGG GAG GCG GAG GCG AAG AAA
EF488996 UUG CAA GCA UUG GAA GUA UAA GUC UCG UUA CCA AGA GUC CAC ACA AUA GAU UAC AAA UUU CUC GCA GGA UUU UCU AGC GGU CUA UUG UCU GCA GUG CCA GUU ACG GUA AUA GGA CUC UAU UUU GUC UAC CUA AAG AUU UCA GCA CAC GUG CGA UCA AUU GUU AAU GAA UAC GGC CGU GGC UAG AAA UCA AAA UGC AGG GAG GCG GAG GCG AAG AAA
EF063707 UUG CAA GCA UUG GAA GUA UAA GUC UCG UUA CCA AGA GUC CAC ACA AUA GAU UAC AAA UUU CUC GCA GGG UUU UCU AGC GGU CUA UUG UCU GCA GUG CCA GUU ACG GUA AUA GGA CUC UAU UUU GUC UAC CUA AAG AUU UCA GCA CAC GUG CGA UCA AUU GUU AAU GAA UAC GGC CGC GGC UAG AAA UCA AAA UGC AGG GAG GCG GAG GCG AAG AAA
FJ460218 GAG AAG AAA GAA UUA CGG AUA UCG CAC UUC AUA AAG CGU CCG ACA AUA GAU UAU AAA UUU CUU GCA GGA UUU UCA AGC GGU CUA UUG GCA GCG GUA CCC GUU ACA GUA AUC GGA CUA UAU UUU GUC UAC CUA AAG AUU UCC AAU CAC GUG CGA UCA AUC GUU AAU GAG UAC GGU CGU GGU UAG AAA UGG CAA UGG CGC GGU UCA AAG AAG ACG
EU244324 UUG CAA GCA UUG GAA GUA UAA GUC UCG UUA CCA AGA GUC CAC ACA AUA GAU UAU AAA UUU CUC GCA GGA UUU UCU AGC GGU CUA UUG UCU GCA AUA CCA GUU ACG GUA AUA GGA CUC UAU UUU GUC UAC CUA AAG AUU UCA GCA CAC GUG CGA UCA AUC GUU AAU GAA UAC GGC CGC GGC UAG AAA UCA AAA UGC AGG GAG GCG GAG GCG AAG AAA
NC_018571 UUG UUG CAA UUG CCG GAA GUU AAA GUC AAC UAC AAC AUA GGC CAA CCA GAU UUC AAG UUU UUA GCA GGA UUU ACU UCG GGC UUC CUG AUA GCU GUA CCA AUU UCA GCA CUU GGC CUA UAU UUG AUC UAC CUA AAG AUU UCU AAA CAC GUG CAA UCG AUA GUU AAU GAA UAC GGU CGU GGU UAG GAA UCG AAA UGG CAU GGG AAG AAG GAG AAG GAA
GU324107 UGU UGC AAU UGC CGG AAG UUA AAA GUC AAC UAC AAC AUA GGC CAA CCA GAU UUC AAA UUU UUA GCA GGA UUU ACU UCG GGC UUC CUG AUA GCU GUA CCA AUU UCA GCA CUU GGC CUA UAU UUG AUC UAC CUA AAG AUU UCU AAA CAC GUG CAA UCA AUA GUU AAU GAA UAC GGU CGU GGU UAG AAA UCG AAA UGG CAU GGG AAG AAG GAG AAG GAA
EU259784 UGU UGC AAU UGC CGG AGG UUU UCA GCC AAC UAC AUC AUA GGC CAA CCA GAU UUC AAG UUU UUA GCA GGA UUU ACU UCG GGC UUC CUG AUA GCU GUA CCA AUU UCA GCA CUU GGC CUA UAU UUG AUC UAC CUA AAG AUU UCU AAA CAC GUG CAA UCG AUA GUU AAU GAA UAC GGU CGU GGU UAG AAA UCG AAA UGG CAA GGG AAG AAG GAG AAG GAA
HQ700826 UUG UUG CAA UUG CCG GAA GUU AAA GUC AAC UAC AAC AUA GGC CAA CCA GAU UUC AAG UUU UUA GCA GGA UUU ACU UCG GGC UUC CUG AUA GCU GUA CCA AUU UCA GCA CUU GGC CUA UAU UUG AUC UAC CUA AAG AUU UCU AAA CAC GUG CAA UCG AUC GUU AAU GAA UAC GGU CGU GGU UAG AAA UCG AAA UGG CAU GGG AAG AAG GAG AAG GAA
HQ700825 UUG UUG CAA UUG CCG GAA GUU AAA GUC AAC UAC AAC AUA GGC CAA CCA GAU UUC AAG UUU UUA GCA GGA UUU ACU UCG GGC UUC CUG AUA GCU GUA CCA AUU UCA GCA CUU GGC CUA UAU UUG AUC UAC CUA AAG AUU UCU AAA CAC GUG CAA UCG AUC GUU AAU GAA UAC GGU CGU GGU UAG AAA UCG AAA UGG CAU GGG AAG AAG GAG AAG GAA
GU324109 UUG UUG CAA UUG CCG GAA GUU AAA GUC AAC UAC AAC AUA GGC CAA CCA GAU UUC AAG UUU UUA GCA GGA UUU ACU UCG GGC UUC CUG AUA GCU GUA CCA AUU UCA GCA CUU GGC CUA UAU UUG AUC UAC CUA AAG AUU UCU AAA CAC GUG CAA UCA AUA GUU AAU GAA UAC GGU CGU GGU UAG AAA UCG AAA UGG CAU GGG AAG AAG GAG AAG GAA
GU324105 UUG UUG CAA UUG CCG GAA GUU AAA GUC AAC UAC AAC AUA GGC CAA CCA GAU UUC AAG UUU UUA GCA GGA UUU ACU UCG GGC UUC CUG AUA GCU GUA CCA AUU UCA GCA CUU GGC CUA UAU UUG AUC UAC CUA AAG AUU UCU AAA CAC GUG CAA UCA AUA GUU AAU GAA UAC GGU CGU GGU UAG AAA UCA AAA UGG CAU GGG AAG AAG GAG AAG GAA
FJ425878 UGU UGC AAU UGC CGG AAG UUA AAA GUC AAC UAC AAC AUA GGC CAA CCA GAU UUU AAA UUU UUA GCA GGA UUU ACU UCG GGC UUC CUG AUA GCU GUA CCA AUU UCA GCA CUU GGC CUA UAU UUG AUC UAC CUA AAG AUU UCU AAA CAC GUG CAA UCA AUA GUU AAU GAA UAC GGU CGU GGU UAG AAA UCG AAA UGG CAU GGG AAG AAG GAG AAG GAA
EU259785 UGU UGC AAU UGC CGG AGG UUU UCA GCC AAC UAC AAC AUA GGC CAA CCA GAU UUC AAG UUU UUA GCA GGA UUU ACU UCG GGC UUC CUG AUA GCU GUA CCA AUU UCA GCA CUU GGC CUA UAU UUG AUC UAC CUA AAG AUU UCU AAA CAC GUG CAA UCA AUU GUU AAU GAA UAC GGU CGU GGU UAG AAA UCG UAA UGG CAU GGG AAG AAG GAG AAG GAA
GU324111 UGU UGC AAU UGC CGG AAG UUA AAA GUC AAC UAC AAC AUA GGC CAA CCG GAU UUC AAA UUU UUA GCA GGA UUU ACU UCG GGC UUC CUG AUA GCU GUA CCA AUU UCA GCA CUU GGC CUA UAU UUG AUC UAC CUC AAG AUU UCU AAA CAC GUG CAA UCA AUA GUU AAU GAA UAC GGU CGU GGU UAG AAA UCG AAA UGG CAU GGG AAG AAG GAG AAG GAA
GU324106 UGU UGC AAU UGC CGG AAG UUA AAA GUC AAC UAC AAC AUA GGC CAA CCA GAU UUC AAA UUU UUA GCA GGA UUU ACU UCG GGC UUC CUG AUA GCU GUA CCA AUU UCA GCA CUU GGC CUA UAU UUG AUC UAC CUA AAG AUU UCU AAA CAC GUG CAA UCA AUA GUU AAU GAA UAC GGU CGU GGU UAG AAA UCG AAA UGG CAU GGG AAG AAG GAG AAG GAA
GU324110 UGU UGC AAU UGC CGG AAG UUA AAA GUC AAC UAC AAC AUA GGC CAA CCA GAU UUC AAG UUU UUA GCA GGA UUU ACU UCG GGC UUC CUG AUA GCU GUA CCA AUU UCA GCA CUU GGC CUA UAU UUG AUC UAC CUA AAG AUU UCU AAA CAC GUG CAA UCA AUA GUU AAU GAA UAC GGU CGU GGU UAG AAA UCG AAA UGG CAU GGG AAG AAG GAG AAG GAA
GU324108 UGU UGC AAU UGC CGG AAG UUA AAA GUC AAC UAC AAC AUA GGC CAA CCA GAU UUC AAA UUU UUA GCA GGA UUU ACU UCG GGC UUC CUG AUA GCU GUA CCA AUU UCA GCA CUU GGC CUA UAU UUG AUC UAC CUA AAG AUU UCU AAA CAC GUG CAA UCA AUA GUU AAU GAA UAC GGU CGU GGU UAG AAA UCG AAA UGG CAU GGG AAG AAG GAG AAG GAA
HQ997896 UGU UGC AAU UGC CGG AAG UUA ACA GUC AAC UAC AAC AUA GGC CAA CCA GAU UUC AAG UUU UUA GCA GGA UUU ACU UCG GGC UUC CUG ACA GCU GUA CCA AUU UCA GCA CUU GGC CUA UAU UUA AUC UAC CUA AAG AUU UCU AAA CAC GUG CAA UCA AUU GUU AAU GAA UAC GGU CGU GGU UAG AAA UCG AAA UGG CAU GGG AAG AAG GAG AAG GAA
NC_010809 UUG UUG CAA UUG CCG GAA GUU UAA GCC AAC UAC AAA AUA GGC CAA GCG GAU UUU AGA UUC GUA GCA GGA UUC ACA UCG GGC UUC CUG GCA GCU AUA CCA CUU UGC GCA CUA GGC CUA UAC CUA AUC UAC CUA AAG AUU UCA GCC CAC GUU CGA UCA AUU GUU AAU GAA UAC GGU CGC GGC UAG GAA UCA AAA UGG CAU GGG AAG GAG GAG ACG GAA
JQ700307 UUG UUG CAA UUG CCG GAA GUU UAA GCC AAC UAC AAA AUA GGC CAA GCG GAU UUC AGA UUC GUA GCA GGA UUC ACA UCG GGC UUC CUG GCA GCU AUA CCA CUU UGC GCA CUA GGC CUA UAC UUA AUC UAC CUA AAG AUU UCA GCC CAC GUU CGA UCA AUU GUU AAU GAA UAC GGU CGC GGC UAG AAA UCA AAA UGG CAU GGG AAG GAG GAG ACG GAA
GU324114 UUG UUG CAA UUG CCG GAA GUU UAA GCC AAC UAC AAA AUA GGC CAA GCG GAU UUC AGA UUC GUA GCA GGA UUC ACA UCG GGC UUC CUG GCA GCU AUA CCA CUU UGC GCA CUA GGC CUA UAC CUG AUC UAC CUA AAG AUU UCA GCC CAC GUU CGA UCA AUU GUU AAU GAA UAC GGU CGC GGC UAG GAA UCA AAA UGG CAU GGG AAG GAG GAG ACG GAA
GU324113 UUG UUG CAA UUG CCG GAA GUU UAA GCC AAC UAC AAA AUA GGC CAA GCG GAU UUC AGA UUC GUA GCA GGA UUC ACA UCG GGC UUC CUG GCA GCU AUA CCA CUU UGC GCA CUA GGC CUA UAC CUA AUC UAC CUA AAG AUU UCA GCC CAC GUU CGA UCA AUU GUU AAU GAA UAC GGU CGC GGC UAG GAA UCA AAA UGG CAU GGG AAG GAG GAG ACG GAA
EU091149 UUG UUG CAA UUG CCG GAA GUU UAA GCC AAC UAC AAA AUA GGC CAA GCG GAU UUU AGA UUC GUA GCA GGA UUC ACA UCG GGC UUC CUG GCA GCU AUA CCA CUU UGC GCA CUA GGC CUA UAC CUA AUC UAC CUA AAG AUU UCA GCC CAC GUU CGA UCA AUU GUU AAU GAA UAC GGU CGC GGC UAG GAA UCA AAA UGG CAU GGG AAG GAG GAG ACG GAA
GU324112 UUG UUG CAA UUG CCG GAA GUU UAA GCC AAC UAC AAA AUA GGC CAA GCG GAU UUC AGA UUC GUA GCA GGA UUC ACA UCG GGC UUC CUG GCA GCU AUA CCA CUU UGC GCA CUA GGC CUA UAC UUA AUC UAC CUA AAG AUU UCA GCC CAC GUU CGA UCA AUU GUU AAU GAA UAC GGU CGC GGC UAG GAA UCA AAA UGG CAU GGG AAG GAG GAG ACG GAA
FJ460217 CUG UUG CAA UUG CCG GAA GUU UAA GCC AAC UAC AAA AUA GGC CAA GCG GAU UUU AGA UUC GUA GCA GGA UUC ACA UCG GGC UUC CUG GCA GCU AUA CCA CUU UGC GCA CUA GGC CUA UAC CUA AUC UAC CUA AAG AUU UCA GCC CAC GUU CGA UCA AUU GUU AAU GAA UAC GGU CGC GGC UAG GAA UCA AAA UGG CAU GGG AAG GAG GAG ACG GAA
EU262628 UUG UUG CAA UUG CCG GAA GUU UAA GCC AAC UAC AAA AUA GGC CAA GCG GAU UUU AGA UUC GUA GCA GGA UUC ACA UCG GGC UUC CUG GCA GCU AUA CCA CUU UGC GCA UUA GGC CUA UAC CUA AUC UAC CUA AAG AUU UCA GCC CAC GUU CGA UCA AUU GUU AAU GAA UAC GGU CGC GGC UAG GAA UCA AAA UGG CAU GGG AAG GAG GAG ACG GAA
FJ460213 UUG UUG CAA UUG CCG GAA GUU UAA GCC AAC UAC AAA AUA GGC CAA GCG GAU UUC AGA UUC GUA GCA GGA UUC ACA UCG GGC UUC CUG GCA GCU AUA CCA CUU UGC GCA CUA GGC CUA UAC CUA AUC UAC CUA AAG AUU UCA GCC CAC GUU CGA UCA AUU GUU AAU GAA UAC GGU CGC GGC UAG GAA UCA AAA UGG CAU GGG AAG GAG GAG ACG GAA
EU091151 UUG UUG CAA UUG CCG GAA GUU UAA GCC AAC UAC AAA AUA GGC CAA GCG GAU UUC AGA UUC GUA GCA GGA UUC ACA UCG GGC UUC CUG GCA GCU AUA CCA CUU UGC GCA CUA GGC CUA UAC CUA AUC UAC CUA AAG AUU UCA GCC CAC GUU CGA UCA AUU GUU AAU GAA UAC GGU CGC GGC UAG GAA UCA AAA UGG CAU GGG AAG GAG GAG ACG GAA
EU091150 UUG UUG CAA UUG CCG GAA GUU UAA GCC AAC UAC AAA AUA GGC CAA GCG GAU UUU AGA UUC GUA GCA GGA UUC ACA UCG GGC UUC CUG GCA GCU AUA CCA CUU UGC GCA CUA GGC CUA UAC CUA AUC UAC CUA AAG AUU UCA GCC CAC GUU CGA UCA AUU GUU AAU GAA UAC GGU CGC GGC UAG GAA UCA AAA UGG CAU GGG AAG GAG GAG ACG GAA
EF063708 UUG UUG CAA UUG CCG GAA GUU UAA GCC AAC UAC AAA AUA GGC CAA GCG GAU UUC AGA UUC GUA GCA GGA UUC ACA UCG GGC UUC CUG GCA GCU AUA CCA CUU UGC GCA UUA GGC CUA UAC CUA AUC UAC CUA AAG AUU UCA GCC CAC GUU CGA UCA AUU GUU AAU GAA UAC GGU CGC GGC UAG GAA UCA AAA UGG CAU GGG AAG GAG GAG ACG GAA
FJ460215 UUG UUG CAA UUG CCG GAA GUU UAA GCC AAC UAC AAA AUA GGC CAA GCG GAU UUC AGA UUC GUA GCA GGA UUC ACA UCG GGC UUC CUG GCA GCU AUA CCA CUU UGC GCA CUA GGC CUA UAU UUA AUC UAC CUA AAG AUU UCA ACC CAC GUU CGA UCA AUU GUU AAU GAA UAC GGU CGC GGC UAG GAA UCA AAA UGG CAU GGG AAG GAG GAG ACG GAA
NC_014545 UUG UUG CAU UAG AGA GAA GCU UAA AGU CAA GAU CAC AGC CUA ACA CUG GAC UAC AAA UUC CUA UCA GGA UUU ACA GCA GGU UUU CUG GUA GCA GUA CCA AUA UCA ACG UUA GGC UUA UAU UUA AUC UAC CUA AAG GUU UCU GCC CAC GUG CGA CAA AUA GUU AAU GAA UAC GGU CGU GGG UAG AAG AAC GAU CAA UGG AAG AAG ACG ACC ACG UAG
GQ379224 UUG UUG CAA UAG AGA GAA GCU UAA AGU CAA GAU CAC AGC CUA ACA CUG GAC UAC AAA UUC CUA UCA GGA UUC ACA GCA GGU UUU CUG GUA GCA GUA CCA AUA UCA ACG UUA GGC UUA UAU UUA AUC UAC CUA AAG GUU UCU GCU CAC GCU CAC GUG CGA CAA AUA GUU AAU GAA UAC GGU CGU GGG UAG AAG AAC GAU UAA UGG AAG AAG ACG ACC ACG UAG
```

HQ827780	[illegible]
JF803842	[illegible]
AJ575129	[illegible]
JF507725	[illegible]
NC_008249	[illegible]
NC_016038	[illegible]
EF126150	[illegible]
EF090905	[illegible]
EF079908	[illegible]
EF079906	[illegible]
EF079905	[illegible]
HQ388349	[illegible]
HQ388350	[illegible]
EF079907	[illegible]
EF079904	[illegible]
JN015068	[illegible]
HQ388351	[illegible]
L40011	[illegible]
L40015	[illegible]
L39986	[illegible]
L39980	[illegible]
L39975	[illegible]
L39976	[illegible]
L39974	[illegible]
L39973	[illegible]
EF079903	[illegible]
L40017	[illegible]
L40014	[illegible]
L40013	[illegible]
L39981	[illegible]
L40012	[illegible]
L39979	[illegible]
L39971	[illegible]
L40016	[illegible]
L39977	[illegible]
NC_003743	[illegible]
JQ862472	[illegible]
L39970	[illegible]
L39969	[illegible]
L39968	[illegible]
L39967	[illegible]
NC_003056	[illegible]
JN674409	[illegible]
L24049	[illegible]
JN674408	[illegible]
JN674407	[illegible]
JN674406	[illegible]
JN674405	[illegible]
JN674404	[illegible]
JN674403	[illegible]
JN674402	[illegible]
AB038148	[illegible]
AB076038	[illegible]
AB038149	[illegible]
AB038150	[illegible]
DQ145545	[illegible]
NC_003369	[illegible]
HM439776	[illegible]
GQ404380	[illegible]
NC_004751	[illegible]
EF521848	[illegible]
DQ988088	[illegible]
EF521839	[illegible]
EF521830	[illegible]
EF521827	[illegible]
D10206	[illegible]
DQ988102	[illegible]
DQ988099	[illegible]
DQ988096	[illegible]
DQ988095	[illegible]
DQ988091	[illegible]
DQ988090	[illegible]
DQ988089	[illegible]
DQ988087	[illegible]
DQ988097	[illegible]
NC_012931	[illegible]
L10356	[illegible]
AF020090	[illegible]
DQ988107	[illegible]
NC_002198	[illegible]
DQ988108	[illegible]
DQ988104	[illegible]
DQ988106	[illegible]
DQ988094	[illegible]
DQ988093	[illegible]
DQ988092	[illegible]
DQ988086	[illegible]
DQ988085	[illegible]
DQ988084	[illegible]
DQ988083	[illegible]
DQ988105	[illegible]
DQ988100	[illegible]
DQ988098	[illegible]
DQ988101	[illegible]
DQ988103	[illegible]
DQ988082	[illegible]
JF925155	[illegible]
JF925153	[illegible]
HQ245318	[illegible]
GU327735	[illegible]
AF369929	[illegible]
AF369928	[illegible]
AF369927	[illegible]
JF925154	[illegible]
JF925152	[illegible]
GU190168	[illegible]
NC_000874	[illegible]
JQ266227	[illegible]
JQ266226	[illegible]
HQ342888	[illegible]
HQ245322	[illegible]
HQ245320	[illegible]
HQ245319	[illegible]
HQ245317	[illegible]
HQ245316	[illegible]
GU190167	[illegible]
GU190166	[illegible]
GU190165	[illegible]
GU190163	[illegible]
GU190162	[illegible]
GU190161	[illegible]
GU190160	[illegible]
GU190159	[illegible]
AM072753	[illegible]
AM072752	[illegible]
AM072751	[illegible]
AY236971	[illegible]
AF369923	[illegible]
HQ245321	[illegible]
AF369926	[illegible]
AM072750	[illegible]
AF369924	[illegible]
JQ266228	[illegible]
AF369925	[illegible]
GU570008	[illegible]
GU570004	[illegible]
GU570007	[illegible]
AJ249447	[illegible]
GU570006	[illegible]
GU190164	[illegible]
JQ266229	[illegible]
AM072756	[illegible]
AM072755	[illegible]
AM072754	[illegible]
NC_021484	[illegible]
NC_010806	[illegible]
NC_021481	[illegible]
KC572000	[illegible]
KC559092	[illegible]
EF408185	[illegible]
AY541037	[illegible]
AY540130	[illegible]
AY541038	[illegible]
AY541039	[illegible]
EF408159	[illegible]
DQ115526	[illegible]
EU332307	[illegible]
DQ631856	[illegible]
EF521834	[illegible]
EF408171	[illegible]
DQ115525	[illegible]
DQ631852	[illegible]
EF521840	[illegible]
DQ115532	[illegible]
EU332332	[illegible]
EU332309	[illegible]
EF521841	[illegible]
DQ631853	[illegible]
DQ631851	[illegible]
DQ631850	[illegible]
DQ631848	[illegible]
AY879231	[illegible]
EF408179	[illegible]
EF408178	[illegible]
EF408176	[illegible]
EF408177	[illegible]
EF408175	[illegible]
EF408174	[illegible]
EF408172	[illegible]
EU332329	[illegible]
EU332328	[illegible]
EF521847	[illegible]
EF521846	[illegible]
EF521845	[illegible]
EF521844	[illegible]
EF521838	[illegible]
EF521837	[illegible]
EF521833	[illegible]
EF521832	[illegible]
EF521831	[illegible]
EF408173	[illegible]
EF043235	[illegible]
DQ631854	[illegible]
DQ631847	[illegible]
EF521836	[illegible]
EF521835	[illegible]
EF521829	[illegible]
DQ115530	[illegible]
EF408169	[illegible]
EF408168	[illegible]
EF408167	[illegible]
EF408166	[illegible]
EF408165	[illegible]
EF408164	[illegible]
EF408163	[illegible]
EF408162	[illegible]
EF408161	[illegible]
EF408160	[illegible]
DQ118372	[illegible]
DQ079612	[illegible]
DQ079611	[illegible]
DQ079610	[illegible]
DQ079609	[illegible]
D11032	[illegible]
X56050	[illegible]
EF521849	[illegible]
EF521843	[illegible]
EF521842	[illegible]
EF408170	[illegible]
DQ631857	[illegible]
DQ115531	[illegible]
D85783	[illegible]
AF235167	[illegible]
EF408156	[illegible]

EU332335	[illegible]
EU332334	[illegible]
EU332312	[illegible]
EU332330	[illegible]
EU332322	[illegible]

Notes

All luteovirid sequences with coverage of ORF3/3a available in GenBank as of 16 Nov 2013 are shown above. The vast majority of the 459 sequences have an intact ORF3a.

Sequences from which NCBI RefSeqs were derived (list follows) have been omitted in favour of the RefSeq accession numbers (NC_004750 - X07653, NC_002160 - AF218798, NC_004666 - AY220739, NC_021481 - KC571999, NC_001747 - D00530, NC_018571 - JQ700308, NC_010809 - EU000534, NC_016038 - HQ388348, NC_008249 - AY956384, NC_012931 - FM865413, NC_002766 - AF352024, NC_000874 - AF157029, NC_014545 - GU167940, NC_002198 - AF235168, NC_004756 - AF473561, NC_003056 - AB038147, NC_010732 - EF529624, NC_015050 - AB594828, NC_003369 - AF441393, NC_021484 - KC921392, NC_010806 - EU024678, NC_006265 - AY695933, NC_021564 - HF679486).

BYDV sequences EU332335, EU332334, EU332312, EU332330 and EU332322 have a 15-codon insertion in ORF3a, but maintain an intact ORF3a and P3a predicted transmembrane domain.

The accession numbers of ORF3a defective-sequences are highlighted in pink. Descriptions are given below.

- NC_004756 and L39976 have single-nucleotide deletions that fuse ORFs 3a and 3.
- NC_003491 and NC_002766 have an AUUG to AUG substitution relative to other sequences from the same species. The substitution introduces a premature termination codon into the non-AUG-initiated ORF3a but allows AUG-initiation of a 5'-truncated ORF3a whose product would lack the predicted transmembrane domain present in full-length P3.
- EF107543 has a two-nucleotide deletion that introduces a premature termination codon into ORF3a. L40012 has a single-nucleotide deletion that introduces a premature termination codon into ORF3a.
- JF925155 has 3 single-nucleotide insertions that maintain ORF3a, but introduce local changes in amino acid sequence. These changes disrupt the predicted transmembrane domain present in the ORF3a product of other luteovirid sequences.
- L10356 has a single-nucleotide deletion and 7 single-nucleotide insertions. These introduce a premature termination codon into ORF3a.
- AJ704819 and HM439608 have single-nucleotide insertions that introduce a premature termination codons into ORF3a (only slightly premature in the case of HM439608).
