# Supplementary figures and images for: Discovery of a Small Non-AUG-Initiated ORF in Poleroviruses and Luteoviruses That Is Required for Long-Distance Movement
Source: PLoS Pathog. 2015 May 6;11(5):e1004868. doi: 10.1371/journal.ppat.1004868 (PMC4422679; doi:10.1371/journal.ppat.1004868)

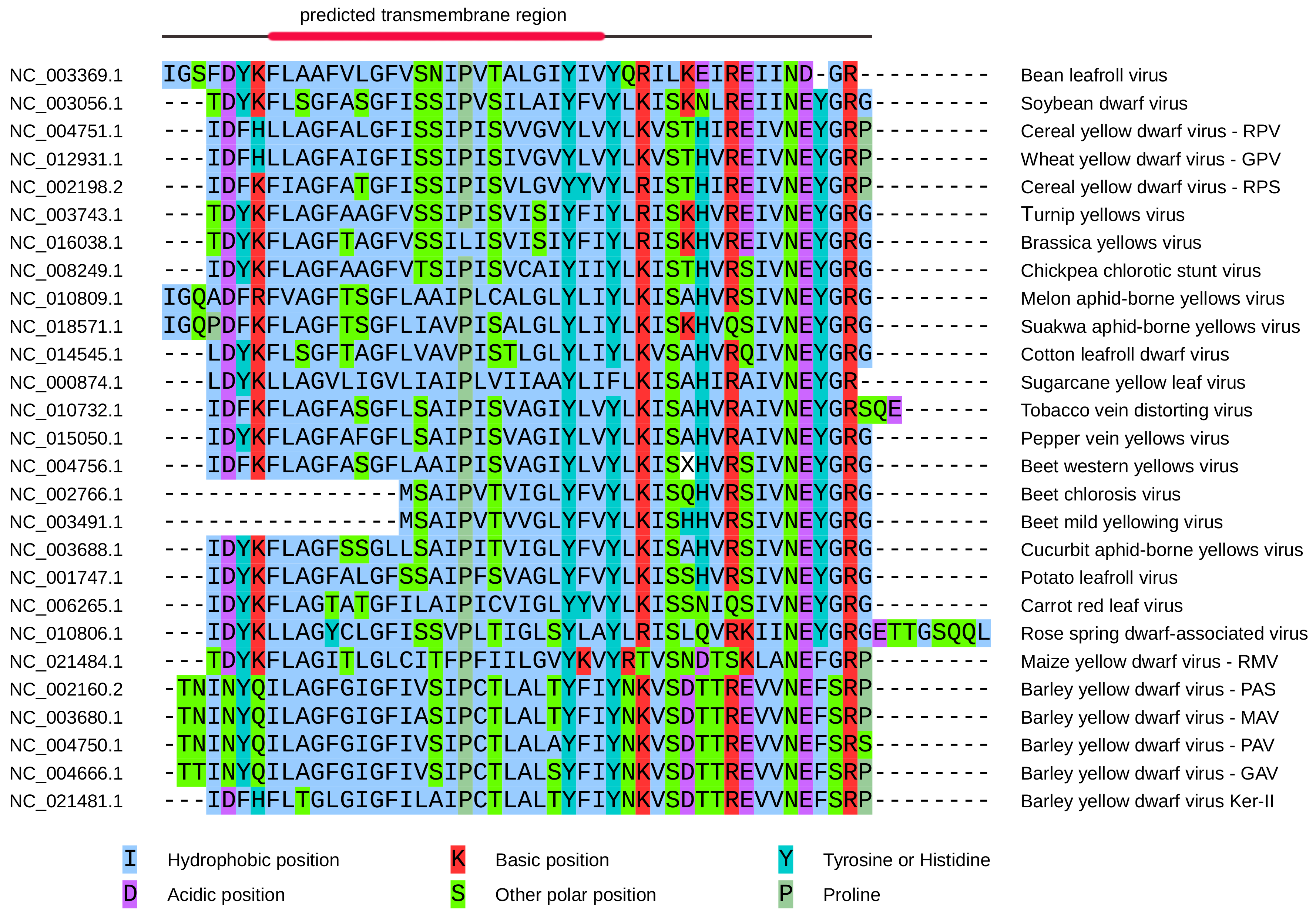

Supplement: S1 Fig — Predicted P3a peptide sequences for representative luteovirus and polerovirus NCBI RefSeq sequences (GenBank accession numbers indicated at left). NC_004756 was translated under the assumption that the single-nucleotide deletion (pink '-' in Fig 2) is a sequencing error (see main text); hence the ambiguous amino acid code 'X' in the NC_004756 P3a sequence. A predicted transmembrane region, conserved in all sequences except the N-terminally truncated Beet chlorosis virus and Beet mild yellowing virus sequences (see text), is indicated above the alignment. Annotated initiation sites are based on the identity and context of potential initiation codons, and comparative sequence analysis. Note that multiple initiation sites may be utilized in some species (e.g. see Fig 2). For illustrative purposes, peptide sequences are shown with the genetic-code decoding of the predicted initiator codon; however, non-AUG initiation codons are expected to be normally decoded by initiator Met-tRNA resulting in an N-terminal methionine, rather than the indicated amino acid, for each sequence. (TIF) [file ppat.1004868.s002.tif]

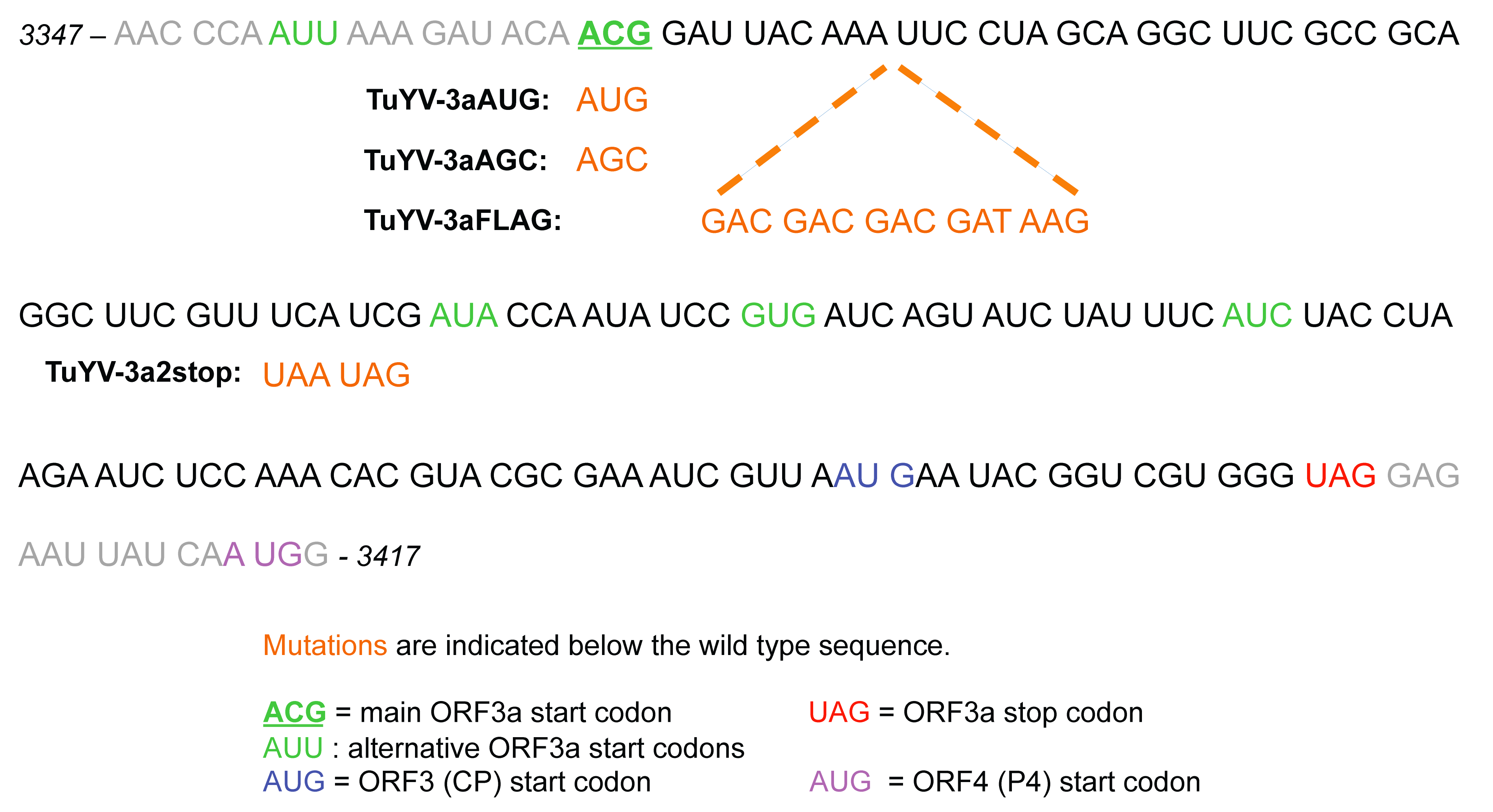

Supplement: S2 Fig — These include substitution mutants TuYV-3aAUG (AUG), TuYV-3aAGC (AGC), TuYV-3a2stop (2stop), and insertion mutant (TuYV-3aFLAG). The ORF3a main initiation codon is in green and underlined; alternative ones are in green. The ORF3a stop codon is depicted in red while the CP and P4 initiation codons are respectively in blue and purple. (TIF) [file ppat.1004868.s003.tif]

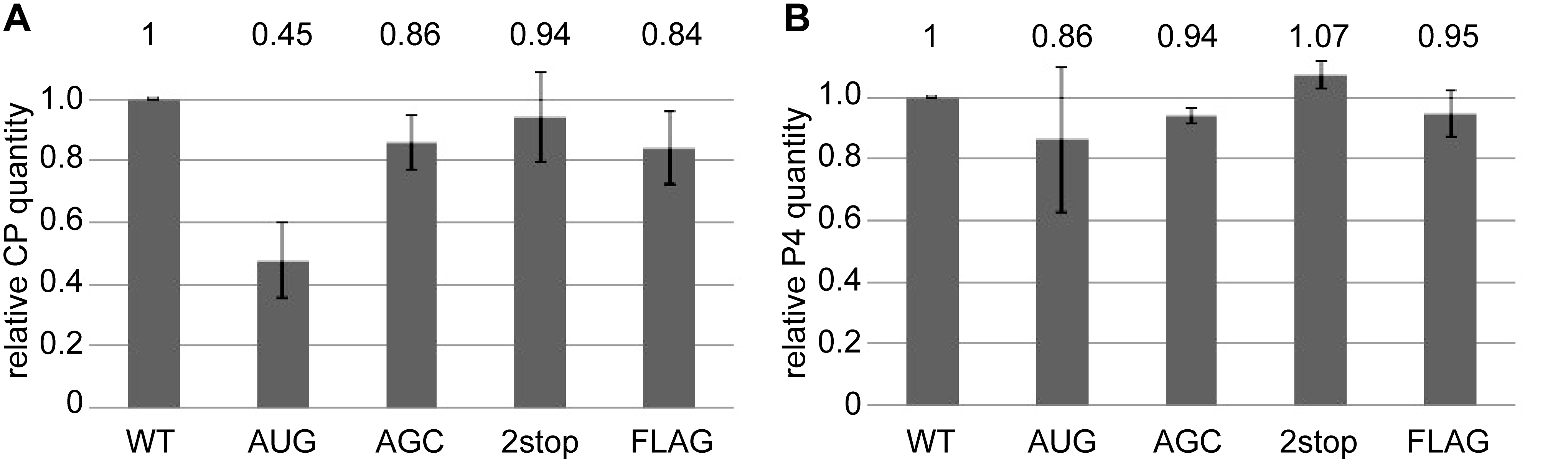

Supplement: S3 Fig — In vitro synthesized subgenomic transcripts were incubated for 30 minutes in wheat germ extracts and radioactive proteins were subsequently fractionated on a 12% PAGE and exposed with a PhosphorImager screen. The bands corresponding to the 20 (P4) and 22 kDa (CP) products were quantified using ImageJ software (http://openwetware.org/wiki/Protein_Quantification_Using_ImageJ). The experiment was repeated twice. WT expression of CP or P4 was arbitrarily fixed to unity in both experiments. (TIF) [file ppat.1004868.s004.tif]

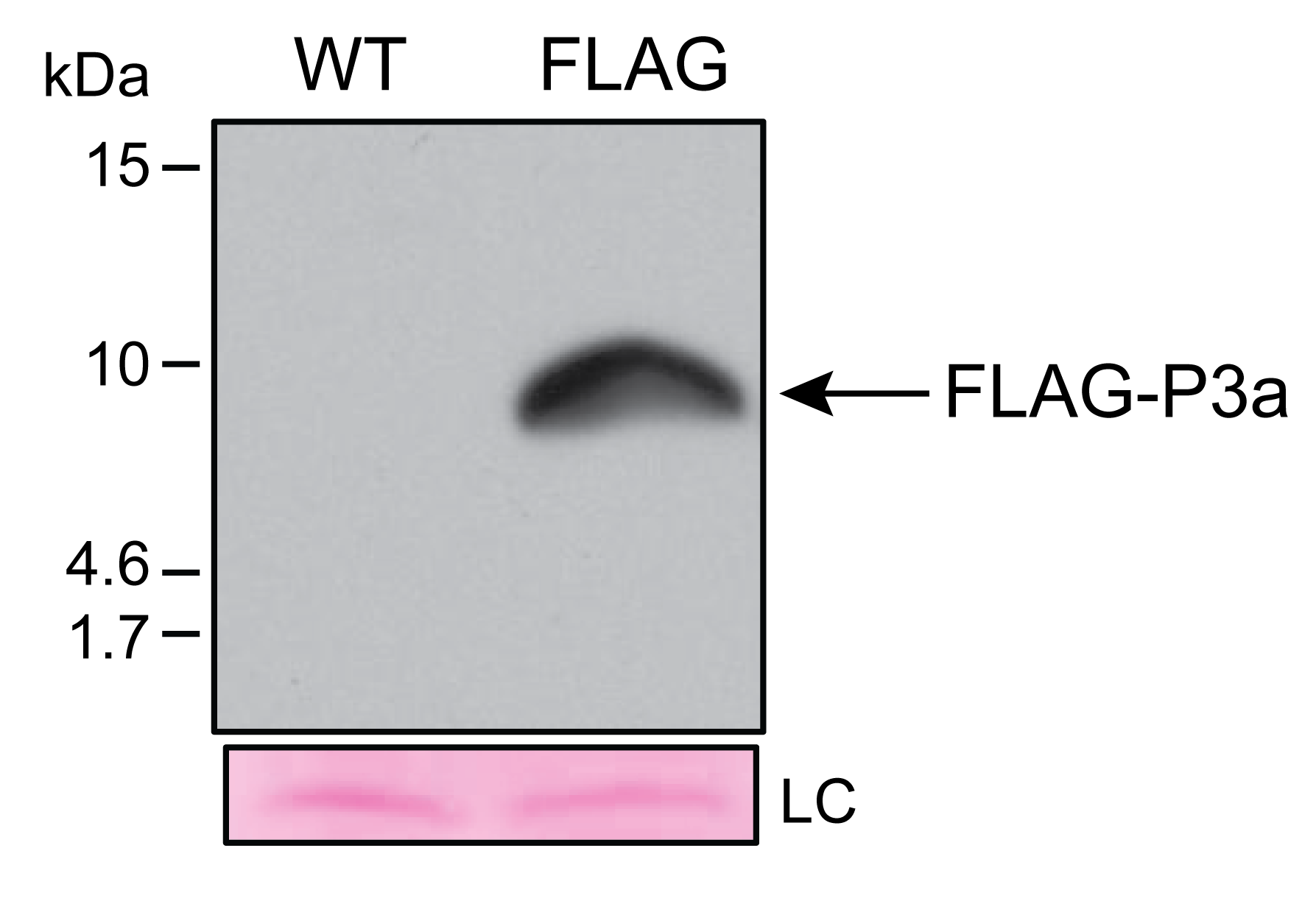

Supplement: S4 Fig — Commercial (SIGMA) specific anti-FLAG antibodies were used. LC, loading control of proteins stained on the membrane by Ponceau red. (TIF) [file ppat.1004868.s005.tif]

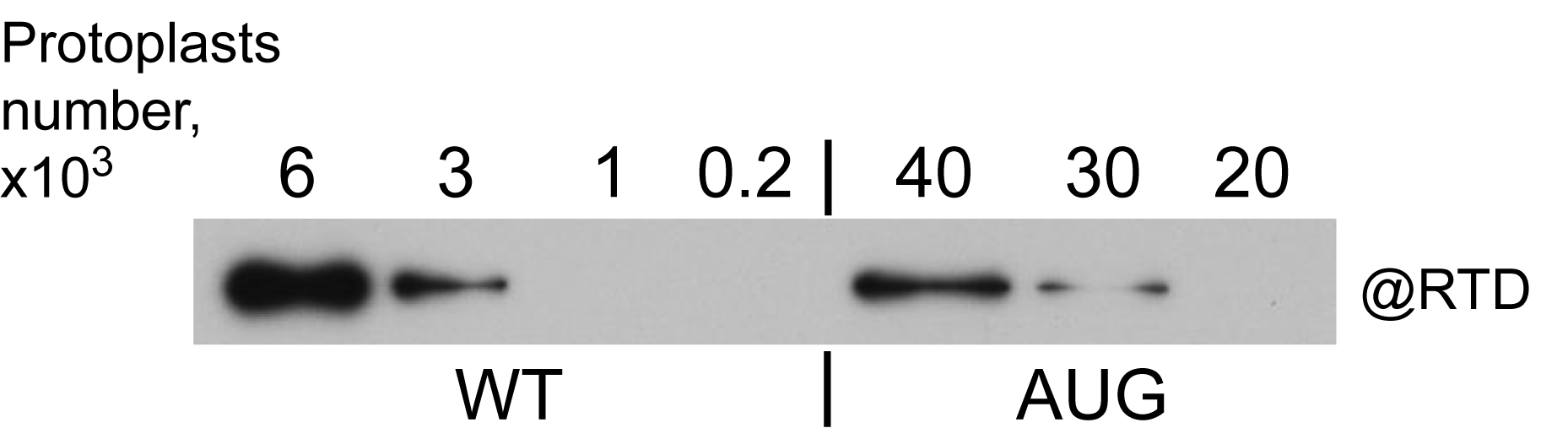

Supplement: S5 Fig — Proteins were detected using specific antibodies against the RTD. (TIF) [file ppat.1004868.s006.tif]

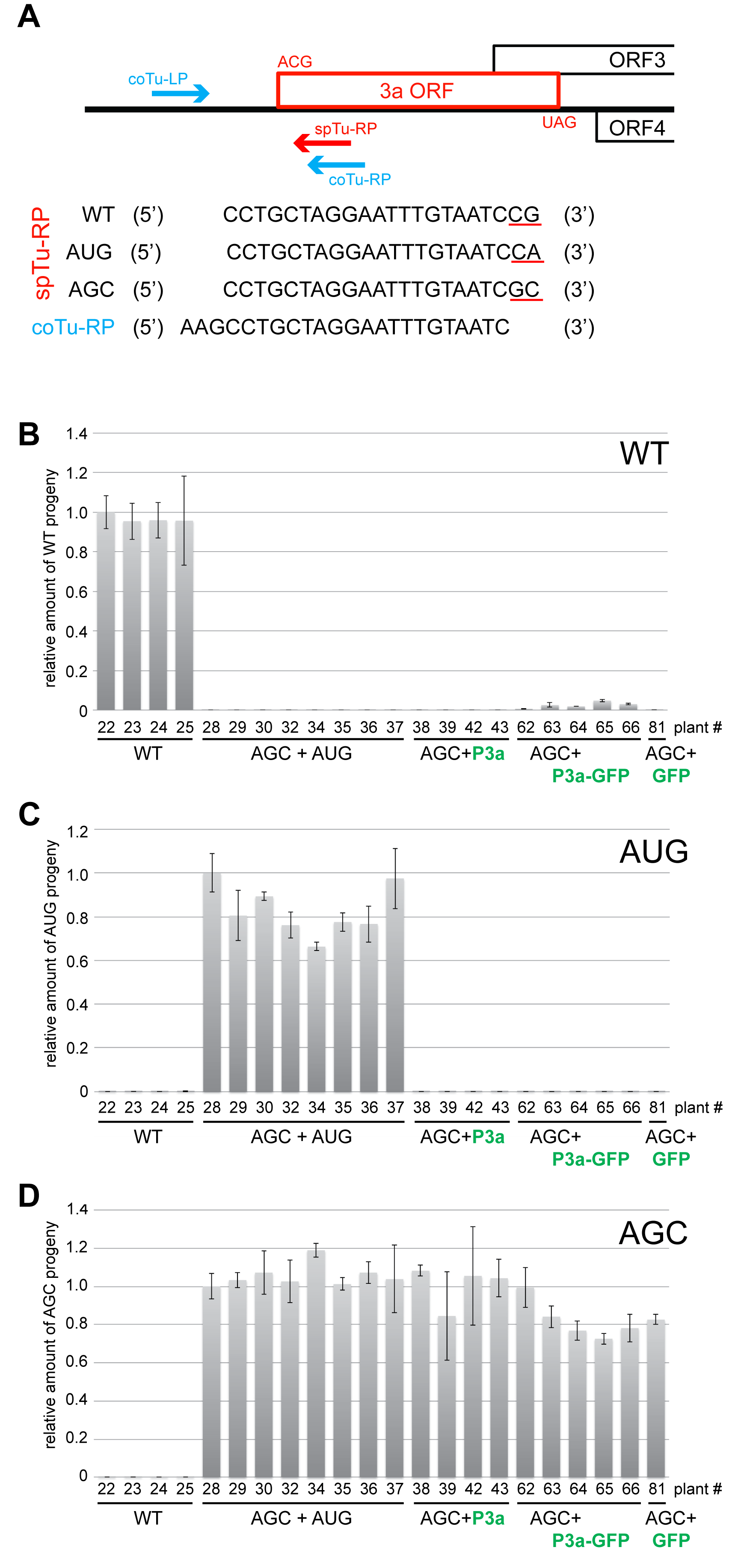

Supplement: S6 Fig — Specific primers were used to detect viral RNA in plants agroinfiltrated with TuYV, TuYV-3aAGC and TuYV-3aAUG, or co-infiltrated with TuYV-3aAGC and agrobacteria transiently expressing P3a, P3a-GFP or GFP. A. Schematic representation of the position of the common primers (coTu-LP and coTu-RP, drawn as blue arrows) and specific primers (spTu-WT/AUG/AGC-RP, drawn as red arrow) used to amplify respectively all viruses or specifically the corresponding WT or mutant virus. The sequences of the common primer coTu-RP and primers specific for the wild-type and each mutant virus are shown, with the bases complementary to the last two bases of the ACG initiation codon (or corresponding mutant sequence) underlined in red. B, C and D. Specific amplification by qRT-PCR of TuYV (WT) (B), TuYV-3aAUG (AUG) (C) and TuYV-3aAGC (AGC) (D) viral progeny from plants infected with the indicated viruses (below the graph): TuYV (WT), TuYV-3aAGC and TuYV-3aAUG (AGC+AUG), or TuYV-3aAGC co-infiltrated with agrobacterium expressing the P3a (AGC+P3a), P3a-GFP (AGC+P3a-GFP) or GFP (AGC+GFP). Proteins names are indicated in green. Samples are from those with the same number in Fig 7. (TIF) [file ppat.1004868.s007.tif]

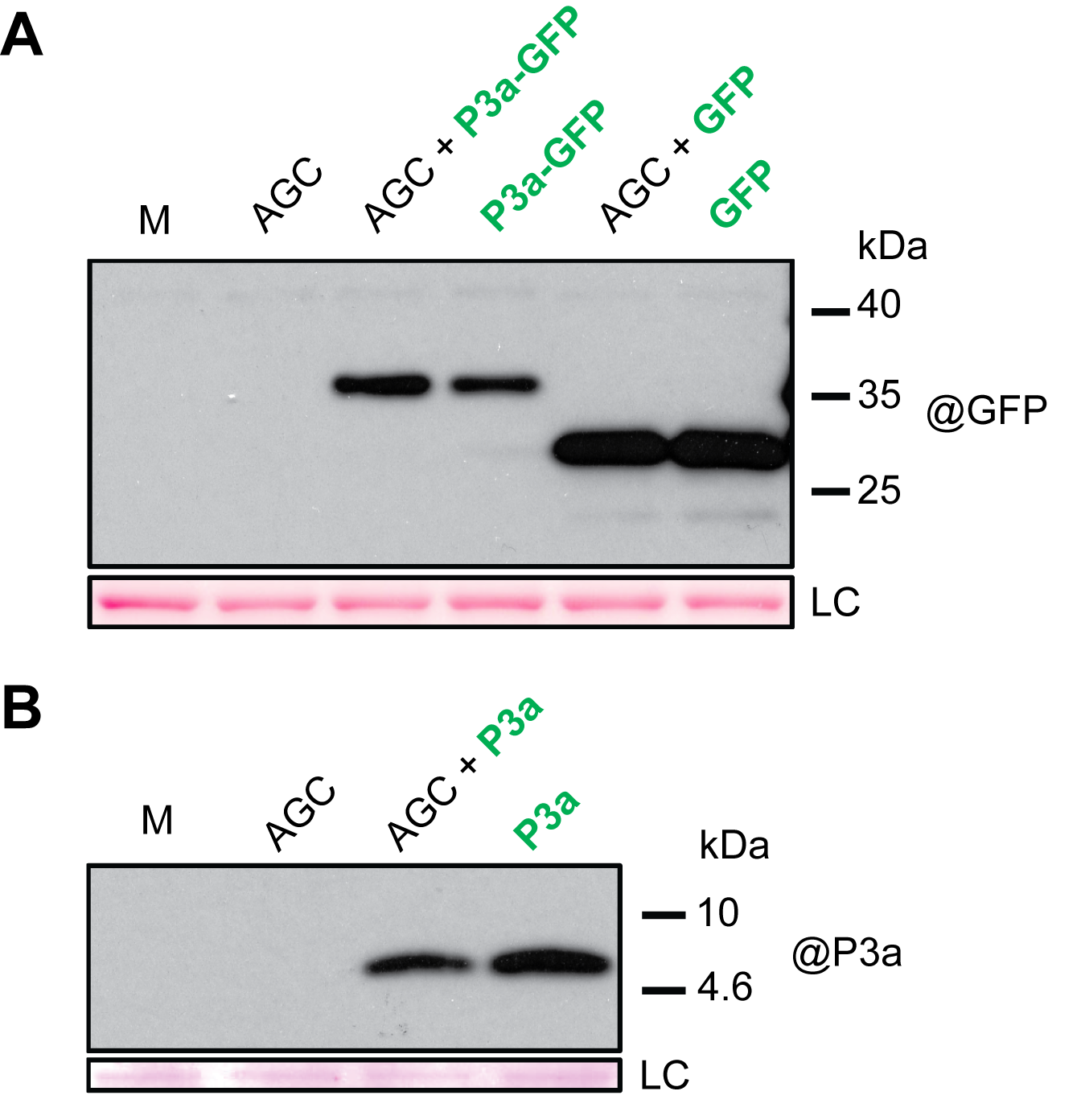

Supplement: S7 Fig — Leaves were infiltrated with the viral mutant TuYV-3aAGC (AGC) or agrobacteria transiently expressing P3a-GFP (P3a-GFP), GFP (GFP), or P3a (P3a), or co-infiltrated with TuYV-3aAGC + P3a-GFP (AGC + P3a-GFP), TuYV-3aAGC + GFP (AGC + GFP) or TuYV-3aAGC + P3a (AGC + P3a). Proteins expressed transiently from agrobacteria are labeled in green. Three days post-infiltration, proteins were detected using specific antibodies against GFP (panel A) or against P3a (panel B). LC, loading control of proteins stained on the membrane by Ponceau red. (TIF) [file ppat.1004868.s008.tif]

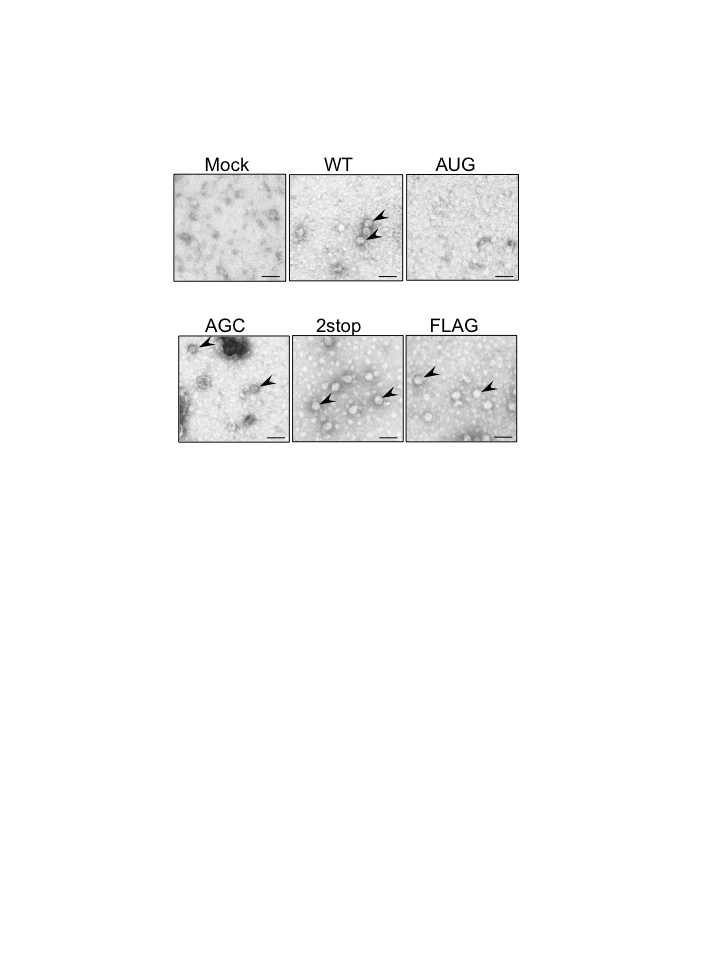

Supplement: S8 Fig — Virions (indicated by arrowheads) were negatively stained after partial purification from C. quinoa protoplasts that had been transfected with the indicated mutant TuYV RNA 48 h previously. Scale bar = 50 nM (TIF) [file ppat.1004868.s009.tif]

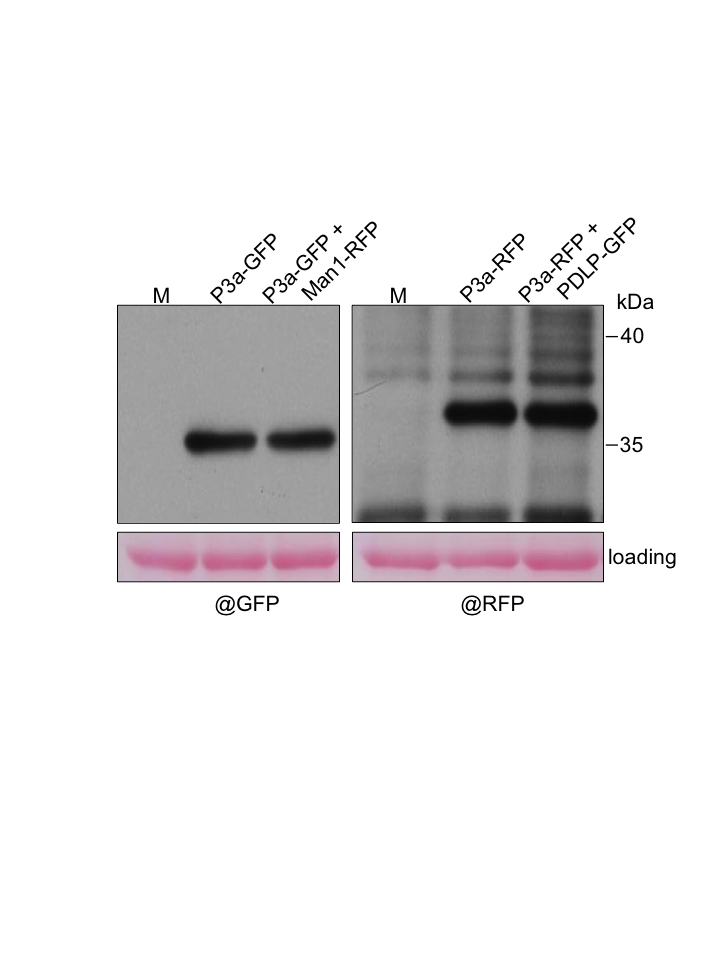

Supplement: S9 Fig — The immunoblots were incubated with antibody specific to GFP or RFP. Loading controls of proteins stained on the membranes by Ponceau red are indicated. (TIF) [file ppat.1004868.s010.tif]

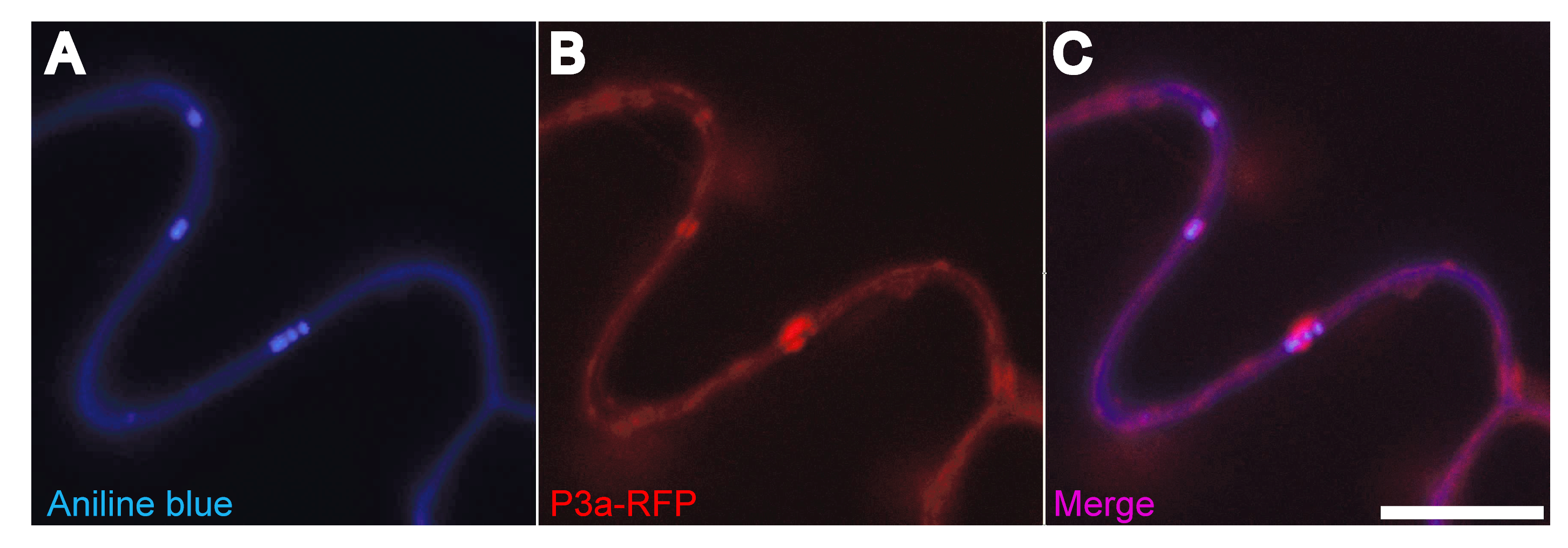

Supplement: S10 Fig — Callose was detected by aniline blue staining (A), in N. benthamiana leaves expressing 3a-RFP (B), and their fluorescent signals merged (C). Scale bar 10 μm. (TIF) [file ppat.1004868.s011.tif]
